# Inhibition of CDK12/CDK13-CYCLIN K rewires P-TEFb signaling to reverse HIV-1 latency and modulate cellular gene expression

**DOI:** 10.1038/s44321-026-00536-z

**Published:** 2026-10-06

**Authors:** Praveenkumar Murugavelu, Jagrity Rani, Heli Glebko, Ina Ambiel, Nadine Tibroni, Nili Tickotsky, Ferdinand Heyn, Philipp Schommers, Liron Levin, Alona Kuzmina, Oliver Till Fackler, Ran Taube

**Affiliations:** 1 The Shraga Segal Department of Microbiology, Immunology and Genetics, Faculty of Health Sciences, Ben-Gurion University of the Negev, Beer Sheva, Israel; 2 Department of Infectious Diseases, Integrative Virology, CIID, Heidelberg University, Medical Faculty Heidelberg, Heidelberg, 69120 Germany; 3 Bioinformatics Core Facility, llse Katz Institute for Nanoscale Science and Technology, Ben-Gurion University of the Negev, Beer-Sheva, Israel; 4 Department of Internal Medicine, Faculty of Medicine and University Hospital Cologne, University of Cologne, 50937 Cologne, Germany; 5 German Center for Infection Research (DZIF), Partner Site Bonn-Cologne, Bonn, Germany; 6 Center for Molecular Medicine Cologne (CMMC), Cologne, 50937 Germany; 7 SynthImmune Cluster of Excellence, Heidelberg University, Heidelberg, Germany; 8 German Center for Infection Research (DZIF), Partner Site Heidelberg, Heidelberg, Germany

**Keywords:** Microbiology, Virology & Host Pathogen Interaction

## Abstract

Persistent HIV-1 latency in long-lived CD4⁺ T-cell reservoirs remains a major obstacle towards viral eradication. Current antiretroviral therapy (cART) exhibits limited clinical efficacy, highlighting the need for new therapeutic interventions. Here, we identify the CDK12/CDK13-Cyclin K (CCNK) complex as a regulator of HIV-1 latency. Pharmacological inhibition of CDK12/CDK13-CCNK with the SR-4835 inhibitor robustly reversed HIV-1 latency in the absence of additional stimuli, both in T cell models and in primary CD4⁺ T cells from people living with HIV-1 receiving cART. Targeting of CDK12/CDK13-CCNK with SR-4835 further synergized HIV-1 latency reversal and enhanced viral reactivation when co-treated with established Latency Reversal Agents. Mechanistically, CDK12/CDK13-CCNK inhibition promoted the release of P-TEFb (CDK9/CYCLIN T) from its inhibitory 7SK snRNP complex, resulting in increased recruitment of RNA polymerase II, CDK9 activation, and remodeled chromatin that overall drove productive HIV-1 transcription. Beyond HIV-1 reactivation, our findings reveal a previously unrecognized compensatory interplay between transcriptional kinases that activate gene expression, thereby rewiring cellular transcriptional programs. Together, this study identifies CDK12/CDK13-CCNK complex as a therapeutic target for HIV-1 latency reversal and provides a mechanistic rationale for improving strategies aimed at achieving an HIV cure.

## Synopsis

The aim of this study was to determine the role of the cellular CDK12/CDK13-CYCLIN K complex in the regulation of HIV-1 latency. For this study, we applied the pharmacological inhibitor SR-4835, which targets CYCLIN K and inhibits the transcription activity of CDK12/CDK13.

- Pharmacological inhibition of CDK12/CDK13-CYCLIN K reactivates latent HIV-1 without any additional stimuli, both in latency T cell models and in primary CD4 T cells derived from HIV-1 patients under therapy.
- Treatment of cells with SR-4835 and inhibition of CDK12/CDK13-CCNK synergize HIV-1 latency reversal when co-treated with Latency Reversal Agents.
- Targeting CDK12/CDK13-CYCLIN K with SR-4835 releases P-TEFb (CDK9/Cyclin T) from the 7SK snRNP complex, thereby stimulating HIV-1 transcription by recruiting RNA Pol II and CDK9 along with reshaping the chromatin landscape around the viral promoter.
- Inhibition of CDK12/CDK13-CYCLIN K enhances a global gene expression program that is mediated by P-TEFb.

## Abstract (web summary)

The aim of this study was to determine the role of the cellular CDK12/CDK13-CYCLIN K complex in the regulation of HIV-1 latency. For this study, we applied the pharmacological inhibitor SR-4835, which targets CYCLIN K and inhibits the transcription activity of CDK12/CDK13.

## Graphical abstract

**Figure:**
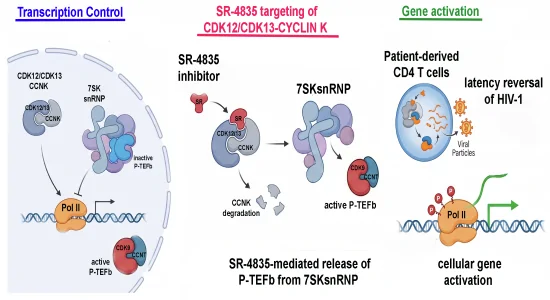

The paper explained **Problem**

Although antiviral therapy effectively suppresses HIV-1 infection, the virus persists primarily within long-lived immune CD4 T cells, rendering a permanent cure out of reach. As a result, new therapeutic interventions are required to efficiently battle HIV-1 infection and achieve complete viral eradication.

**Results**

To overcome this barrier, we investigated the molecular role of the cellular transcription regulator—the CDK12/CDK13-CCNK complex—in HIV-1 latency and show that pharmacological inhibition of CDK12/CDK13-CCNK with the compound SR-4835, successfully reactivates latent HIV-1, without any additional stimuli, both in latency T cell models, and in primary CD4 T cells derived from HIV-1 patients under therapy. Targeting of CDK12/CDK13-CCNK with SR-4835 further synergizes HIV-1 latency reversal and enhances viral reactivation when co-treated with clinically established Latency Reversal Agents (LRAs). At the cellular level, targeting CDK12/CDK13-CCNK complex releases the cellular transcription factor, P-TEFb (CDK9/Cyclin T) from its inhibitory 7SK snRNP complex, thereby enhancing RNA Polymerase II transcription and CDK9 recruitment, along with reshaping the chromatin landscape around the viral promoter and driving productive HIV-1 transcription.

**Impact**

Our findings uncover a previously unrecognized compensatory interplay between transcriptional kinases that rewire the cellular gene expression program, offering critical therapeutic implications for HIV-1 latency reversal, while shedding light on how key cellular kinases cooperate to reshape transcriptional networks.

## Introduction

The persistence of human immunodeficiency virus (HIV-1) remains a central obstacle for a complete cure against viral infection. While antiretroviral therapy (ART) effectively suppresses active viral replication, it fails to eliminate latently infected cells, which persist as reservoirs of infected cells that carry transcriptionally silent but replication-competent provirus. The reservoir of HIV-1 infected cells, consisting primarily of resting memory CD4+ T cells, persists for decades and therefore, virus replication can rapidly rebound upon ART interruption, urging the need for a lifelong therapy (Dahabieh et al, 2015; Dufour et al, 2021). A central mechanism for the establishment and maintenance of HIV-1 latency is the low levels of host and viral transcription factors (TFs) and the efficient block of RNA-Polymerase II (Pol II) transcription at the pause-release step, overall leading to suppression of productive viral gene elongation (Dahabieh et al, 2015; Dufour et al, 2021). Deep understanding of metazoan gene transcription control is therefore essential for revealing mechanisms of HIV-1 gene expression regulation and for the development of therapeutic interventions. Indeed, the cell transcription program is a multi-step process that is highly coordinated by TFs and their kinase partners, which jointly execute gene expression in a dynamic environment (Core and Adelman, 2019; Field and Adelman, 2020). Steps of the transcription cycle include initiation and recruitment of DNA-binding TFs to their promoters, followed by binding of pioneer proteins and chromatin-modifying complexes that remodel chromatin and expose core promoter elements at transcription start sites. Cellular kinases play significant roles in promoting the transcription program. TFIIH and Mediator associate with TF and RNA Polymerase II (Pol II), allowing Pol II to bind to the promoter and initiate mRNA synthesis (Allen and Taatjes, 2015; Chen et al, 2018; Core and Adelman, 2019; Muse et al, 2007; Schier and Taatjes, 2020; Scruggs and Adelman, 2015). CDK7 then phosphorylates serine residues at positions 5 and 7 of the C-terminal Domain (CTD) of Pol II (Eick and Geyer, 2013; Schier and Taatjes, 2020). While Ser5P attracts capping enzymes to disrupt interactions between Pol II and Mediator and enhance promoter clearance, Ser7P primes the CTD for subsequent phosphorylation and transcription elongation (Czudnochowski et al, 2012; Glover-Cutter et al, 2009; Wong et al, 2014). DRB sensitivity-inducing factor (DSIF/SPT4/5) and the negative elongation factor (NELF) also assemble onto PIC to induce Pol II pausing (Adelman and Lis, 2012; Luse et al, 2020; Vos et al, 2018). Only upon recruitment of the Positive Transcription Elongation Factor b (P-TEFb; CDK9; CYCLIN T) and Super Elongation Complex, the paused Pol II is released for elongation. Pol II pause-release is catalyzed by CDK9 that phosphorylates SPT5, NELF, as and the Ser2-CTD (Luo et al, 2012; Yamaguchi et al, 2013). As key TF, P-TEFb activity is tightly regulated in human cells. While the active complex is free to associate with Super Elongation Complex, the inactive complex resides within the 7SK snRNP and serves as a P-TEFb reservoir (C Quaresma et al, 2016; Nguyen et al, 2001; Yang et al, 2005; Yang et al, 2001). CDK12 and CDK13 also play pivotal roles in the regulation of HIV-1 gene expression. Both share a high degree of structural similarity and overlapping functions, as they mediate Ser2 phosphorylation and enhance transcription elongation (Barboric and Fujinaga, 2016; Bartkowiak et al, 2010; Blazek et al, 2011; Greenleaf, 2019; Price, 2000; Wei et al, 1998; Zhou et al, 2012). CDK12 also ensures proper expression of DNA damage response genes, cell cycle progression, and RNA processing (Emadi et al, 2020; Liang et al, 2020; Tellier et al, 2020). It plays key roles as a “genome integrity guardian”, maintaining genome stability and DNA repair - primarily of long genes. Genome-wide analyses show that CDK12 occupancy overlaps with actively transcribed genes and enhancer regions, reinforcing its role as a regulator of a subset of genes rather than exerting uniform control over transcription. Inactivation or loss of CDK12 have been linked to genomic instability, tumorigenesis, and altered cellular responses to DNA damage, making it a promising biomarker and therapeutic target for diverse human cancers (Choi et al, 2020; Paculova and Kohoutek, 2017; Wu et al, 2023). Inhibition of CDK12/CDK13 has been shown to induce G2/M arrest, occurring due to the accumulation of DNA damage (Dubbury et al, 2018). CDK13, on the other hand, is involved in splicing and mRNA processing (Blazek et al, 2011; Greenleaf, 2019; Morison et al, 2023). These unique regulatory functions of CDK12 in transcription and DNA repair underline its importance in both normal cellular physiology and disease contexts (Houles et al, 2023; Lei et al, 2022; Lui et al, 2018; Wang et al, 2023). Nevertheless, while the effects of CDK12/CDK13 on gene transcription have been thoroughly explored, the role of these targets as potential Latency Reversal Agents of HIV-1 have not been studied.

In this study, we aimed to further examine the role of CDK12/CDK13 in the regulation of HIV-1 gene expression and viral latency. To target CDK12/CDK13-CYCLIN K (CCNK), HIV-1-infected cells were treated with SR-4835, a CCNK degrader that exhibits optimal selectivity for CDK12/CDK13-CCNK, with low off-target effects. SR-4835 acts as a molecular glue that recruits CDK12/CDK13-CCNK to the CUL4-RBX1-DDB1 ubiquitin ligase complex, targeting CCNK for degradation (Bosken et al, 2014; Dieter et al, 2021; Kozicka et al, 2024; Quereda et al, 2019; Schmitz et al, 2024). SR-4835 is highly specific for CDK12 and CDK13, when compared to other currently applied CDK12/CDK13 inhibitors like CDK12 kinase inhibitor, THZ531 (Zhang et al, 2016). Molecular modeling studies using the X-ray structure of CDK12 predict that the binding of SR-4835 is ATP-competitive, with SR-4835 interacting via hydrogen bonding with the hinge kinase region. Depending on the approach for calculating the IC_50_ values of SR-4835 for CDK12 and CDK13, values are in the range of 60 nM for CDK12/CCNK and 366 nM for CDK13/CCNK. Other approaches determined IC_50_ values of in the range of 84.3 to 156 nM for CDK12/CCNK and about 400 nM for CDK13/CCNK. Dissociation constants (*K*_D_) of SR-4835 for CDK12-CCNK or CDK13-CCNK are reported in the range of 94 nM or 168 nM, respectively. Importantly, SR-4835 displays no inhibitory activity towards other kinases like CDK7, CDK8, and CDK9, and a moderate inhibition for CDK10, which is 10-fold lower (Bosken et al, 2014; Quereda et al, 2019; Schmitz et al, 2024). Overall, based on these analyses, SR-4835 targets CDK12/CDK13-CCNK with higher selectivity than any other known CDK12-targeting inhibitor. Moreover, it is more efficient toward CDK12 than CDK13 and exhibits higher affinities toward CDK12/CDK13-CCNK than other kinases (Bosken et al, 2014; Quereda et al, 2019; Schmitz et al, 2024). These unique properties of SR-4835 make it a potential drug for treating human cancers (Pilarova et al, 2020; Tadesse et al, 2021). Our current results show that treating HIV-1-infected cells with the SR-4835 CDK12/CDK13-CCNK inhibitor solely induces viral-specific and cellular gene activation. Importantly, synergistic reactivation of latent HIV-1 gene expression is detected upon inhibiting CDK12/CDK13-CCNK with SR-4835 and treating infected cells with selected standard Latency Reversing Agents (LRAs). Globally, treatment of cells with SR-4835 also induces cellular gene activation, which is mediated by P-TEFb. Overall, our findings suggest a novel interplay between cellular kinases that compensate each other and induce a gene activation program, mediated by P-TEFb. Such gene activation program rewires the cellular gene expression program and has clinical implications for HIV-1 latency reversal and cancer biology.

## Results

### Targeting CDK12/CDK13-CCNK with SR-4835 induces gene expression of HIV-1

**Figure 1:**
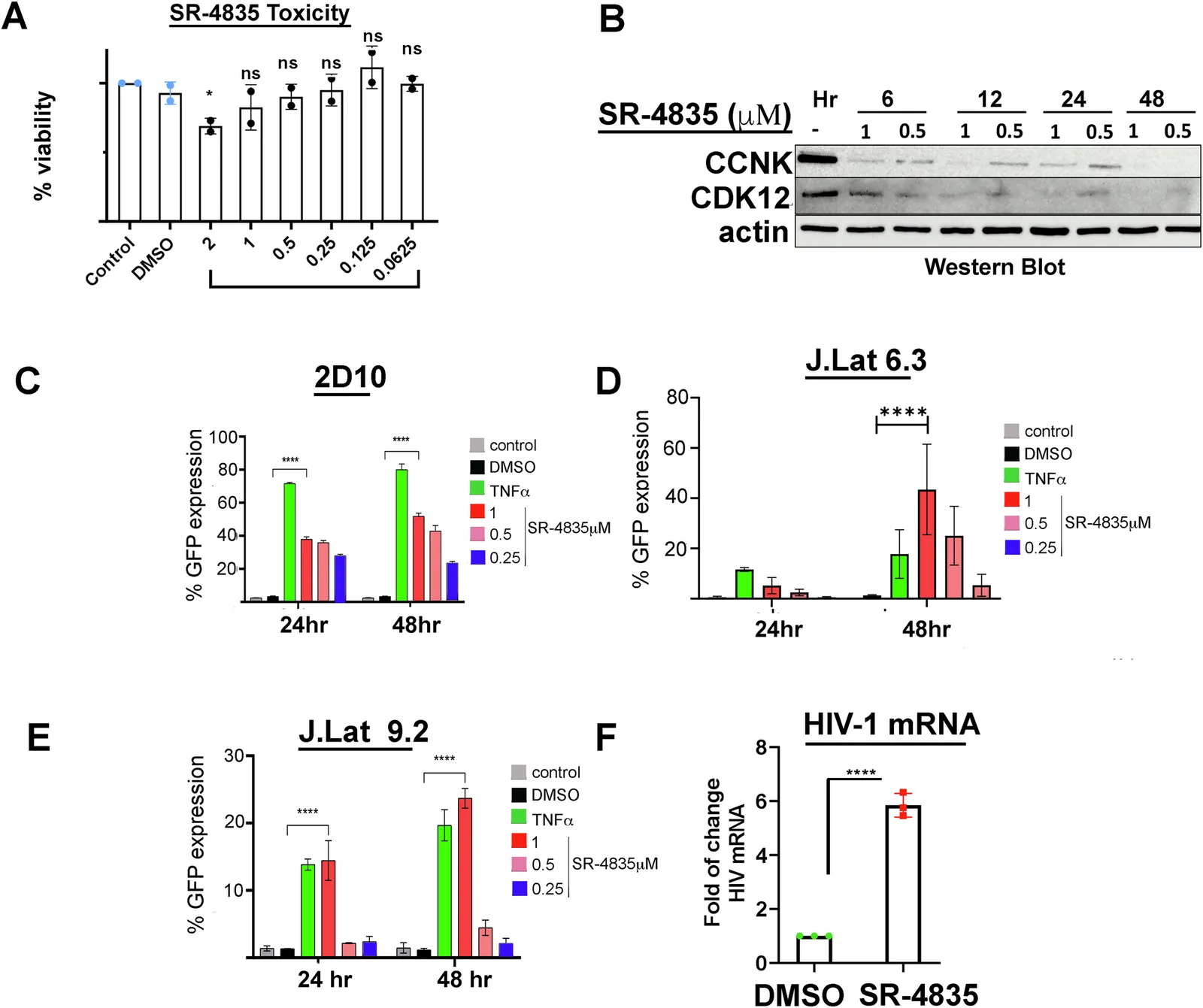
Targeting CDK12/CDK13-CCNK reactivates HIV-1 gene expression and reverses latency. (**A**) Measurements of cell viability. Cells were incubated with SR-4835 for 2 h and then washed. Cells were harvested at 48 h post treatment. XTT assays were performed on Jurkat T cells treated with increasing concentrations of SR-4835. Data represent mean ± SD from three biological replicates. Statistical significance was determined by a two-tailed test. *P = 0.0445 and ns: non-significant. (**B**) Levels of CDK12 and CCNK expression following SR-4835 treatment – Jurkat T cells were incubated with SR-4835 for the indicated time points (6;12;24;48 h), washed, and harvested before being subjected to Western Blot analysis with CDK12 and CCNK antibodies. (**C**–**E**) 2D10 cells (**C**); J-Lat 6.3 (**D**) and J-Lat9.2 (**E**) were treated with increasing concentrations of SR-4835 for 2 h before washing. TNFα was used as a positive control for T cell activation and HIV-1 reactivation. Cells were harvested and monitored by FACS after 24 and 48 h post-treatment. Data represent mean ± SD from three biological replicates. Statistical significance was calculated using two-way ANOVA followed by Tukey’s multiple comparisons test (****P = 0.0001). (**F**) HIV-1 mRNA levels were detected in J-Lat 9.2 cells at 24 h post SR-4835 reactivation by employing qPCR using primers located on the LTR viral promoter. Data are presented relative to GAPDH and represent mean ± SD from three independent experiments. Statistical significance was assessed using an unpaired two-tailed t-test. ****P = 0.0001.

To elucidate the role of CDK12/CDK13-CCNK in the regulation of gene transcription, we took advantage of the HIV-1 gene expression program, which is heavily regulated by cellular machinery. This regulation primarily occurs at steps of Pol II pause release and elongation of transcription, where CDK12/CDK13 play key roles together with P-TEFb, therefore serve as a model for studying gene transcription regulation. HIV-1-infected cells were treated with the highly specific CDK12/CDK13-CCNK inhibitor, SR-4835, which was applied to several HIV-1-infected Jurkat-derived CD4 T cells, which serve as models for studying HIV-1 gene expression. 2D10 Jurkat cells carry an integrated HIV-based lentiviral cassette, where the regulatory viral proteins Tat (H13L) and Rev are expressed in cis, and coupled to a destabilized GFP reporter gene under the control of the HIV-1 LTR. HIV-1 in 2D10 cells is easily reactivated upon stimulation with TNFα, standard LRAs or T-cell activation agents (Greenleaf, 2019; Pearson et al, 2008). Jurkat J-Lat 9.2 and J-Lat 6.3 Jurkat clones are infected with HIV-1 and serve as latency models. J-Lat clones carry a replication-defective full-length HIV-1 provirus, where GFP is inserted instead of the *nef* gene, enabling direct assessment of HIV-1 proviral transcriptional activation from the integrated LTR upon T cell stimulation (Jordan et al, 2001). Treatment of these cells with increasing concentrations of SR-4835 for 2 h exhibited relatively low toxicity (around 20–25%), enabling us to target CDK12/CDK13 and do not harm cell viability (Fig. 1A). We further confirmed by Western Blotting that indeed treating T cells with SR-4835 depleted CDK12/CCNK protein levels (Fig. 1B). Treating 2D10 cells with SR-4835 induced a clear and dose-dependent increase in HIV-1 GFP expression, with viral reactivation levels substantially rising compared to untreated controls cells, or stimulation with TNFα (Fig. 1C). Similarly, in J-Lat 6.3 (Fig. 1D) and J-Lat 9.2 (Fig. 1E) cells, where HIV-1 is relatively more difficult to be reactivated, SR-4835 treatment led to a significant increase in viral reactivation, as detected by increased levels of GFP expression. Finally, we also monitored HIV-1 RNA levels by RT-qPCR in J-Lat 9.2 cells, confirming an increase upon treating cells with SR-4835 (Fig. 1E,F). These results confirmed that targeting of CDK12/CCNK by SR-4835 effectively reverses HIV-1 latency and reactivates viral gene expression.

### Targeting CDK12/CDK13-CCNK with SR-4835 induces minimal cell apoptosis

**Figure EV1:**
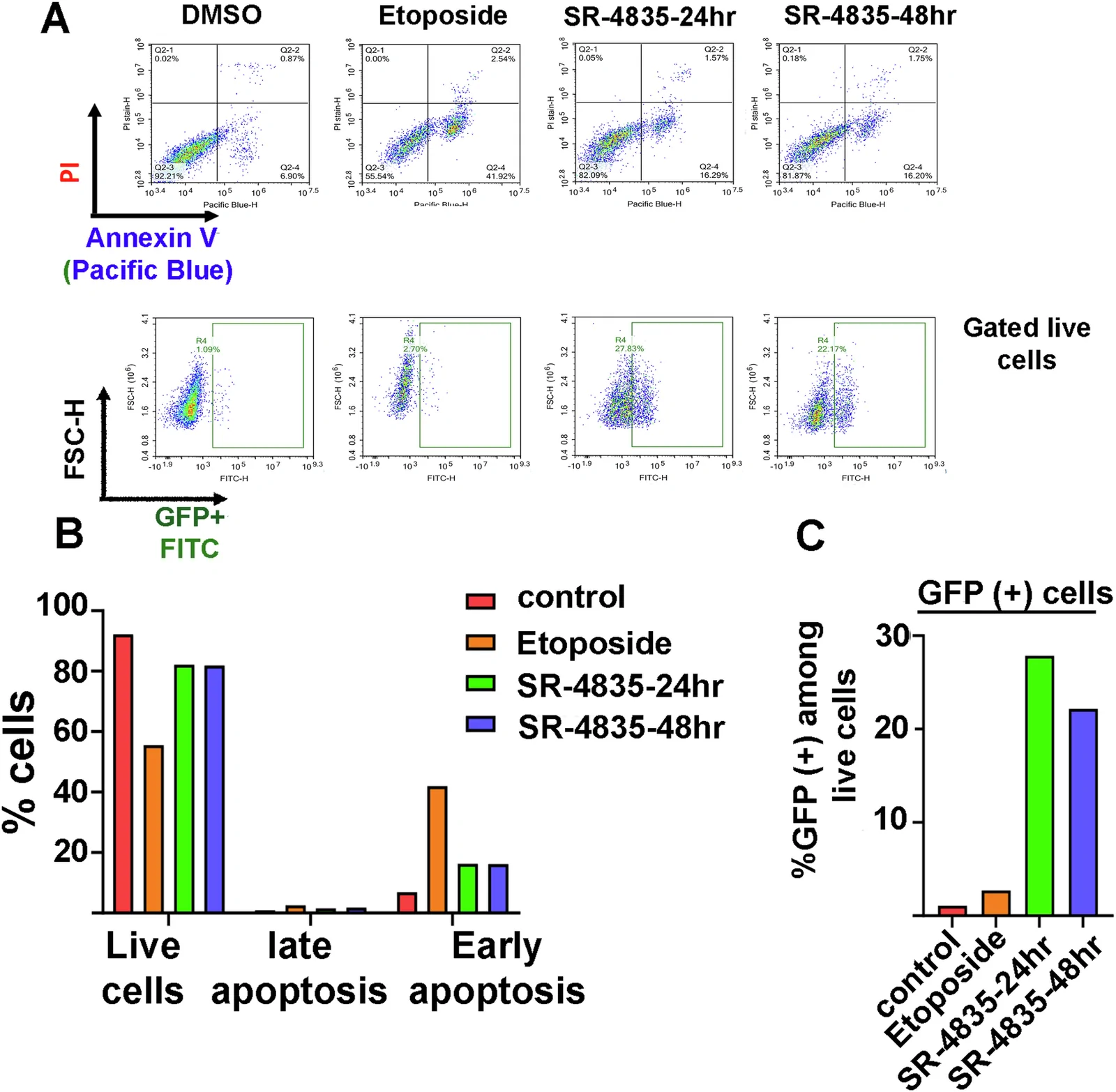
Targeting CDK12/CDK13-CCNK with SR-4835 induces minimal cell apoptosis. Cell apoptosis was monitored in 2D10 cells using Annexin V-Pacific Blue and Propidium Iodide (PI) staining. Cells were treated with SR-4835 for 2 h, and at the indicated time points were harvested and stained for Annexin V-pacific blue and PI before being analyzed by FACS. (**A**) FACS dot plots after 24 and 48 h post-treatment with SR-4835. Treated cells were analyzed by flow cytometry and gated only on live cells (Annexin V(-)/PI(-)), excluding apoptotic or necrotic perturbations in treated cells. Etoposide-treated cells (10 mM) served as a positive control for cell apoptosis. (**B**) Quotative analysis of FACS plots in each category; live cells (Annexin V-/ PI-); early apoptotic (Annexin V-/ PI+); Late apoptosis (Annexin V+/ PI+). (**C**) Quotative analysis of HIV-GFP expression following treatment of cells with SR-4835. Gated only on live cells.

Apoptosis and cellular stress may induce HIV-1 non-specific reactivation (Khan et al, 2015). Therefore, it was critical to accurately assess whether SR-4835 treatment directly reactivates latent HIV-1 or triggers stress and cell apoptosis. We monitored apoptosis levels by FACS upon treating 2D10 cells with SR-4835, verifying that HIV-1 reactivation indeed occurred specifically in viable cells, which did not undergo apoptosis. Treated cells were analyzed by flow cytometry and gated only to live cells [Annexin V(-)/PI(-)], excluding apoptotic or necrotic perturbations in SR-4835-treated cells (supplementary; Fig. EV1A). Our observations also showed that treatment of cells at higher concentrations of SR-4835 led to minimal levels of apoptosis. Of note, HIV-1 reactivation was monitored only in live gated cells (supplementary; Fig. EV1B), allowing assessment of HIV-1 gene activation independently of apoptosis and ensuring accurate measurements of HIV-1 transcriptional activation in viable cells.

### Synergistic activation of latent HIV-1 gene expression by combining CDK12/CDK13-CCNK targeting and Latency Reversal Agents

**Figure 2:**
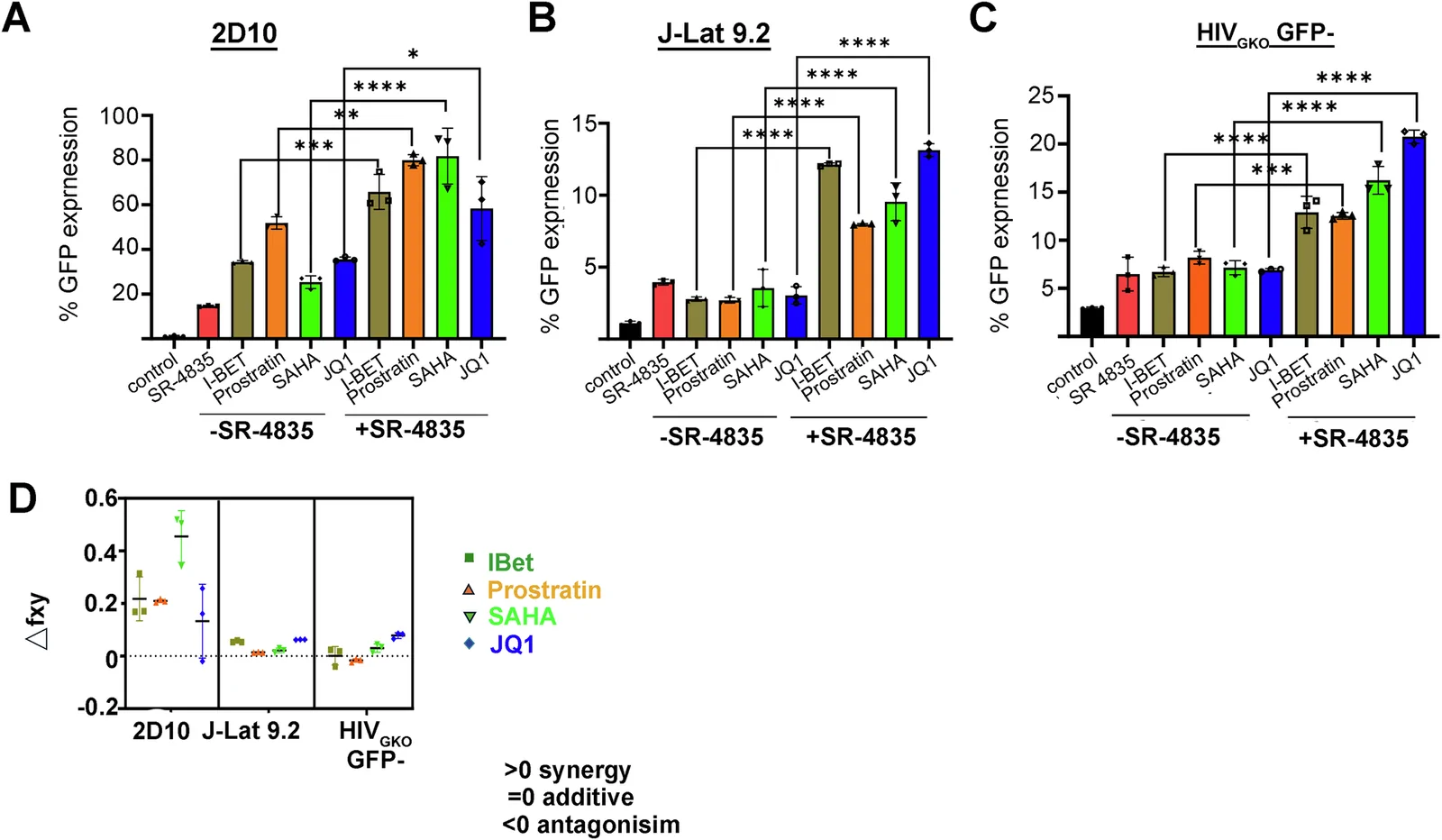
Targeting CDK12/CDK13-CCNK synergizes reactivation effects of latency-reversing agents. (**A**–**C**) 2D10 (**A**), J-Lat 9.2 cells (**B**), and GFP-negative sorted HIV-1_GKO_-transduced Jurkat T cells (**C**) were treated with SR-4835 (0.5 µM) for 2 h. Cells were washed and further grown with media alone, or media supplemented with the indicated LRA - I-BET (1 µM), Prostratin (1 µM), SAHA (1 µM), or JQ1 (1 µM), either alone (DMSO as control). 48 h post-treatment, HIV latency reversal was assessed by flow cytometry based on GFP expression. A combined treatment of LRA + SR-4835 was monitored to assess synergistic reactivation relative to LRA alone. Data represent mean ± SD from three biological replicates. Statistical significance was calculated by one-way ANOVA; (**A**) (2D10); *P* = 0.0004: iBET vs iBET+SR4835; *P* = 0.0016: Prostratin vs Prostratin+SR4835; *P* = 0.0001: SAHA vs SAHA + SR4835; *P* = 0.0132: JQ1 vs JQ1 + SR4835. (**B**) (J-Lat 9.2): *P* = 0.0001: iBET vs iBET+SR4835; *P* = 0.0001: Prostratin vs Prostratin+SR4835; *P* = 0.0001: SAHA vs SAHA + SR4835; *P* = 0.0001: JQ1 vs JQ1 + SR4835. (**C**) (HIV_GKO_ GFP-): *P* = 0.0001: iBET vs iBET+SR4835; *P* = 0.0010: Prostratin vs Prostratin+SR4835; *P* = 0.0001: SAHA vs SAHA + SR4835; *P* = 0.0001: JQ1 vs JQ1 + SR4835. (**D**) Bliss independence analysis of drug combinations showing synergy between SR-4835 and LRAs. Positive Bliss scores indicate synergistic effects- Δfaxy > 0; an additive effect Δfaxy = 0; and an antagonistic effect Δfaxy < 0.

We next tested synergistic effects of SR-4835-mediated reactivation of HIV-1 latency upon combining treatment of cells with currently applied LRAs. 2D10 Cells were treated with SR-4835 for 2 h, followed by treatment of cells with LRAs - Prostratin (1 μM), SAHA (1 μM), or JQ1 (1 μM) and I-BET, each reactivating HIV-1 latency via different pathways (Conrad et al, 2017; de Vries et al, 2025; Margolis et al, 2020; Margolis and Browne, 2025; Williams et al, 2004). We applied such a protocol on three experimental HIV-1 latency models: 2D10, J-Lat 9.2 Jurkat T cell line, and Jurkat T cells infected with HIV-1_GKO_ and sorted for those cells that only carry latent HIV-1 [GFP(-)/mKO2(+)]. HIV-1_GKO_ is a dual-reporter full-length HIV-1 lentivirus, where GFP is expressed under the control of the viral LTR promoter, while mKO2 expression is driven by the EF1α promoter (Battivelli et al, 2018). Cells were treated with SR-4835 alone or in combination with the indicated LRA, and HIV-1 reactivation was monitored by FACS. In all three tested latency cell models, treatment with SR-4835 also induced a statistically significant increase in HIV-GFP(+) expression, confirming the ability of SR-4835 to reverse HIV-1 latency and activate viral gene expression. Significantly, combined treatment of SR-4835 with LRAs led to an enhanced reactivation of latent HIV-1 gene expression (Fig. 2). In 2D10 cells, the combined treatment with SR-4835 and I-BET or SAHA led to a significant increase (4-fold). JQ1 also exhibited a synergistic latency reversal when combined with SR-4835 (Fig. 2A; 4**-**fold). Other LRAs, like Prostratin and SAHA, had no synergistic effects in 2D10 cells. As expected for J-Lat 9.2 cells, SR-4835 or the LRAs alone activated HIV-1 gene expression, but to a lesser extent than in 2D10 cells (Fig. 2B). Combined treatment with SR-4835 and JQ1 or I-BET led to the highest level of latency reversal of about 5-fold. Combined treatments of SR-4835 with SAHA or Prostratin also exhibited low synergism (Fig. 2B). GFP(-)/mKO2(+) HIV-1-infected Jurkat T cells also exhibited an increase of HIV-1 GFP expression upon treatment of cells with SR-4835 or with LRAs alone. Synergy of SR-4835 with JQ1 (4-fold), SAHA (3-fold), Prostratin (x3 fold) and I-BET (2.5-fold) was also documented (Fig. 2C). Finally, a Bliss independence analysis confirm synergism effect of SR-4835 with the indicated LRAs (Fig. 2D). These results suggest that SR-4835 has the potential to enhance LRA-mediated HIV-1 gene activation and should be exploited in combination with LRAs to synergize HIV-1 latency reversal.

### HIV-1 gene activation through targeting CDK12/CDK13-CCNK by SR-4835 is mediated by active CDK9

**Figure 3:**
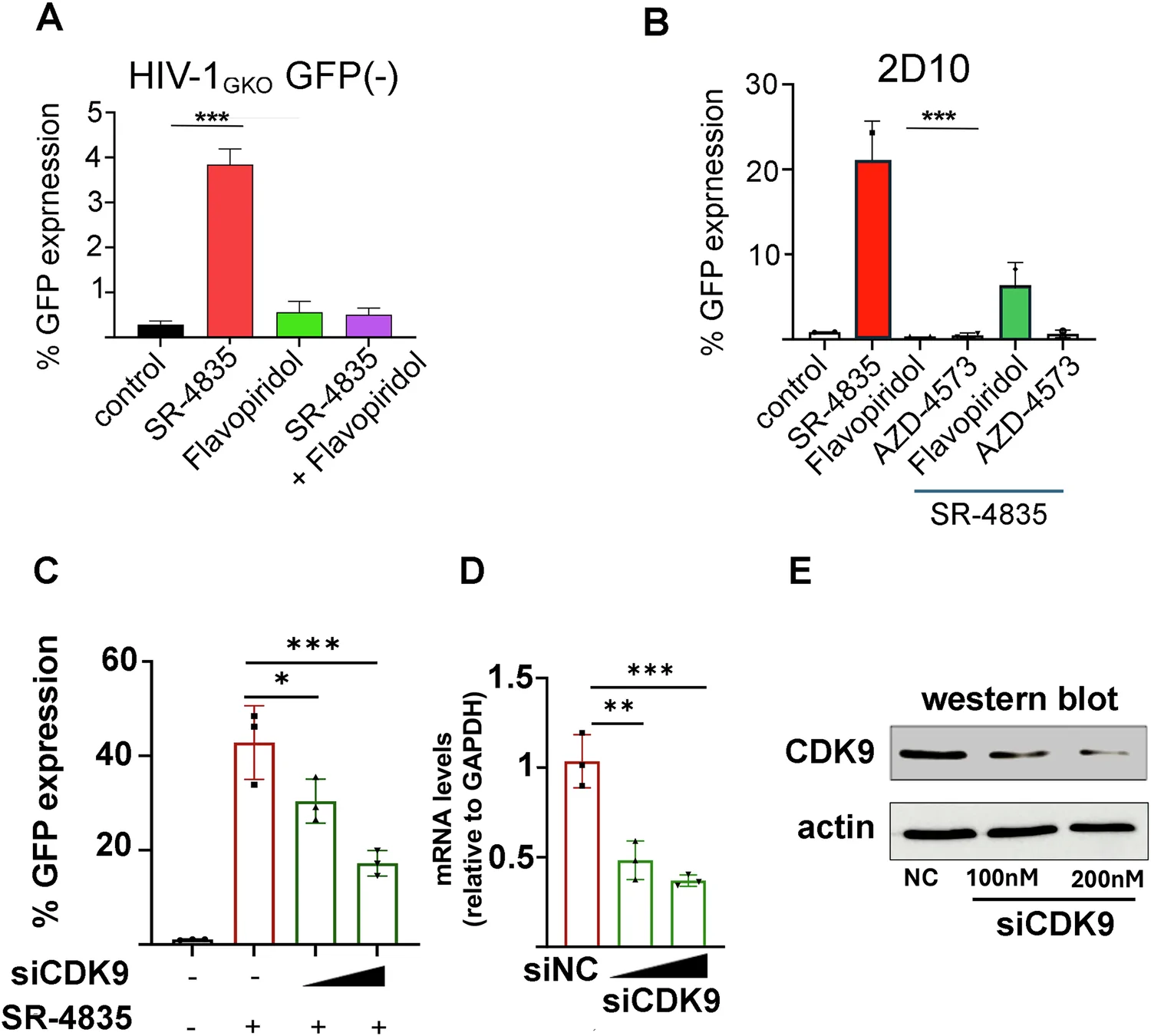
The kinase activity of CDK9 is required for HIV-1 gene activation upon targeting CDK12/CDK13-CCNK with SR-4835. (**A**, **B**) HIV-1_GKO_ infected Jurkat T cells that were sorted for cells where HIV-1 is latent [(GFP(-);mKO2(+)] (**A**), or 2D10 Jurkat T cells (**B**) were treated for 2 h with SR-4835. Following washing, cells were treated with the indicated CDK9 inhibitor Flavopiridol (100 nM) or AZD4573 (25 nM) for an additional 24 h. HIV-1-GFP gene expression was monitored by FACS 24 h post CDK9-drug treatment. Data represent mean ± SD from three independent experiments. Statistical significance was assessed using one-way ANOVA analysis Tukey’s multiple comparisons test ***P = 0.0002. (**C**) Knockdown of CDK9 abolished SR-4835-mediated HIV-1 activation. 2D10 Jurkat CD4 T cells were electroporated with siRNA targeting CDK9 (100 nM or 200 nM). 48 h post-electroporation, cells were treated with DMSO or with SR-4835 for 2 h. Cells were then washed and further grown for 24 h before monitoring HIV-1-GFP expression. Data represent mean ± SD from three independent experiments. Statistical significance was assessed using one-way ANOVA analysis – Tukey’s multiple comparisons test. *P = 0.0307: siNC vs siCDK9_100 nM & ***P = 0.0004: siNC vs siCDK9_200 nM. (**D**) qPCR analysis confirming CDK9 expression following electroporation with siRNA targeting CDK9. Cells were harvested 48 h post electroporation with a CDK9-specific siRNA or scramble siRNA and subjected to RT-qPCR with specific primers for CDK9. Data represent mean ± SD from three independent experiments. Statistical significance was assessed using one-way ANOVA analysis – Tukey’s multiple comparisons test. **P = 0.0018: siNC vs siCDK9_100 nM & ***P = 0.0007: siNC vs siCDK9_200 nM. (**E**) Western blot analysis monitoring CDK9 expression following siRNA electroporation. Cells were harvested 48 h post-electroporation with a CDK9-specific siRNA or scramble siRNA and cell lysate was subjected to Western blot with a CDK9 IgG. Actin levels were used to ensure equal protein load.

As P-TEFb/CDK9 is key for HIV-1 gene activation and Pol II pause-release, we sought to determine whether CDK9 kinase activity is required for the reactivation of HIV-1 gene expression and latency reversal induced by CDK12/CDK13-CCNK targeting by SR-4835. Cells carrying latent HIV-1 [GFP(-);mKO2(+)] were treated with SR-4835 (0.5 µM) for 2 h alone, washed, and then added with fresh media, or media supplemented with Flavopiridol (1 µM), a potent and selective inhibitor of CDK9 kinase activity. 24 h post-treatment HIV-1 latency reversal was monitored by FACS. Our analysis confirmed that expression of HIV-1-GFP was elevated upon targeting CDK12/CDK13-CCNK with SR-4835. However, SR-4835-mediated HIV-1 latency reversal was efficiently diminished (x8-fold) when cells were further treated with Flavopiridol. In the presence of Flavopiridol, HIV-1 gene reactivation was also diminished (Fig. 3A). The inability of Flavopiridol to reactivate HIV-1 gene expression strengthens the hypothesis that targeting of CDK12/CDK13-CCNK by SR-4835 activates HIV-1 gene expression through P-TEFb, suggesting that CDK9 kinase activity is essential in SR-4835 induced latency reversal of HIV-1 (Fig. 3). The requirement for CDK9 was further tested with a second highly specific CDK9 inhibitor - AZD-4573 (Fig. 3B). Our analysis confirmed the above results with Flavopiridol, showing that treating cells with SR-4835, followed by treatment of cells with AZD-4573, inhibited HIV-1 gene expression (Fig. 3B). Finally, to further demonstrate that CDK9 is indeed essential for HIV-1 gene reactivation following SR-4835 treatment of 2D10 cells, we introduced CDK9-specific siRNAs followed by treatment of cells with SR-4835 (Fig. 3C). Our analysis showed that indeed, reducing CDK9 protein levels inhibited HIV-1 gene expression by SR-4835 (Fig. 3C). Reduction of CDK9 expression levels was confirmed by RT-qPCR as well as by western blot (Fig. 3D,E).

### Targeting CDK12/CDK13-CCNK disrupts CDK9-HEXIM1 association and releases P-TEFb from 7SKsnRNP

**Figure 4:**
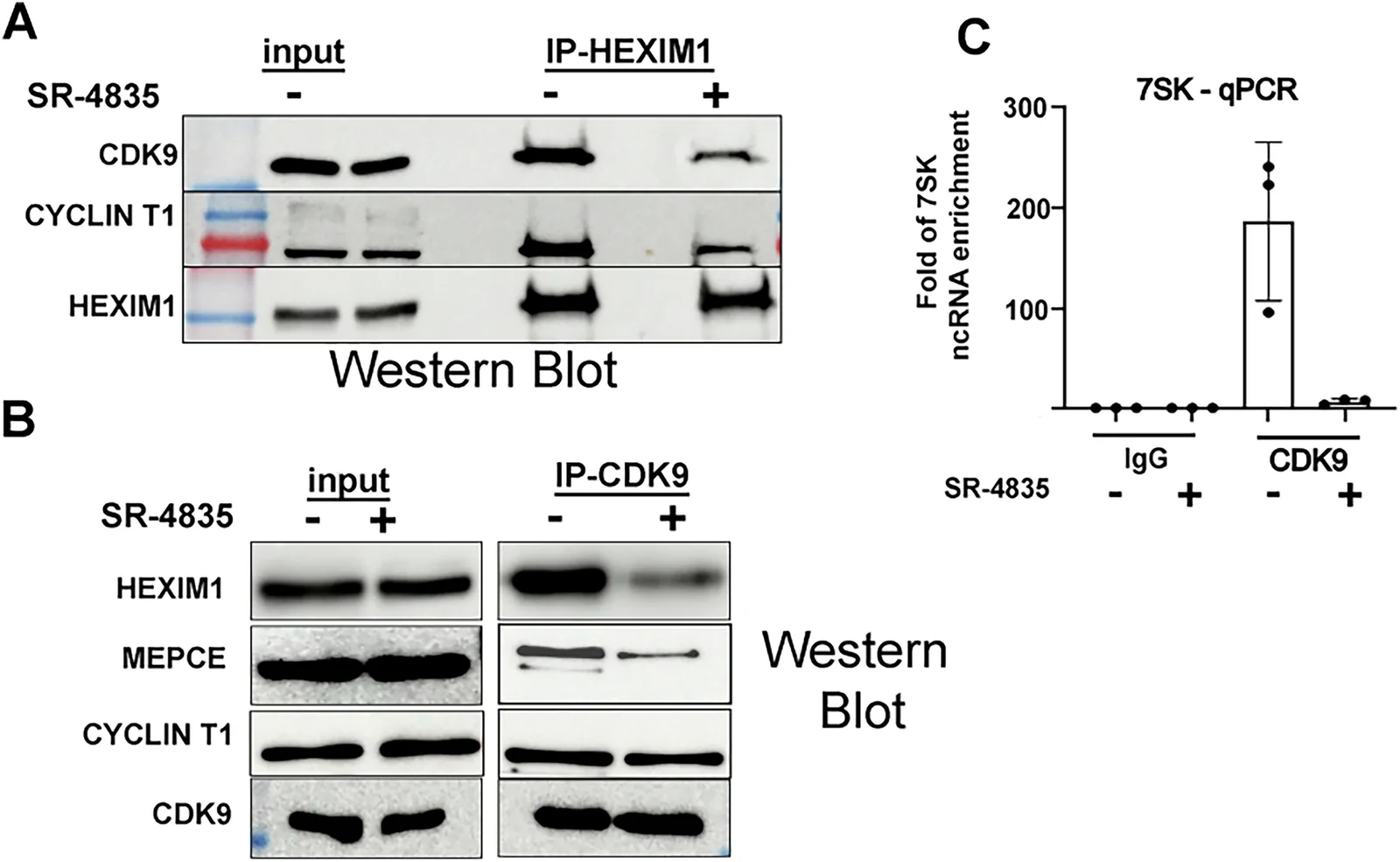
CDK12/CDK13-CCNK targeting by SR4835 disrupts the association of CDK9 and CYCLIN T1 with 7SKsnRNP. (**A**, **B**) 2D10 Jurkat T cells were treated with SR-4835 (0.5 µM) or with DMSO for 2 h, followed by washing. 24 h post-treatment, cells were lysed and immunoprecipitated (IP) with an anti-HEXIM1 IgG (**A**), or anti-CDK9 IgG (**B**). IP fractions were then analyzed by Western blotting for HEXIM1, CDK9, CYCLIN T1 or MEPCE proteins. (**C**) SR-4835 treatment frees P-TEFb from 7SKsnRNA. 2D10 cells were treated with SR-4835 for 6 h or treated with DMSO as a control. Cells were washed with PBS and resuspended in RNA-IP buffer. Cells were lysed and IP with a CDK9 IgG at cold temperature. IP fraction was pre-blocked, and protein G beads were added and incubated for an additional 2 h in cold. Beads were washed with washing buffer supplemented with RNase A inhibitor and protease inhibitor. The pellet was resuspended in lysis buffer, and RNA was extracted and subjected to RT-qPCR with the indicated primers targeting 7SKsnRNA. Input RNA was extracted and reverse transcribed the same way. Dilutions of input were used for the standard curve and calculations. Data are presented as mean ± SD from *n* = 3 independent biological replicates.

Upon establishing that targeting of CDK12/CDK13-CCNK by SR-4835 plays a key role in the reactivation of latent HIV-1 through CDK9/P-TEFb, we aimed to investigate whether this effect of SR-4835 is due to elevated levels of CDK9 expression or as a result of P-TEFb release from its cellular inhibitory 7SKsnRNP complex. In cells, a significant fraction of CDK9/CYCLIN T is sequestered in an inactive state within the 7SKsnRNP complex, consisting of HEXIM1/2, LARP7, MEPCE, and 7SKsnRNA, where HEXIM1/2 binds to CYCLIN T and inhibits the kinase activity of CDK9 (Michels et al, 2003). This interaction prevents CDK9-mediated phosphorylation of Pol II at Ser2 of the CTD, thereby maintaining transcription at a paused state (Ott et al, 2011). To explore the association of P-TEFb and 7SKsnRNP in cells treated with SR-4835, we performed immunoprecipitation (IP) with CDK9 or HEXIM1 antibodies, monitoring the release of P-TEFb from its 7SKsnRNP inhibitory complex by western blotting (Fig. 4). Our results show that in SR-4835 treated cells, following HEXIM1 IP, there was a marked decrease in CDK9 protein levels that were associated with HEXIM1. Similar results were obtained with CYCLIN T1 (Fig. 4A). Importantly, total protein levels of CDK9 and HEXIM1 in the input lysates remained unchanged following SR-4835 treatment, confirming that the effect on HIV-1 gene activation was due to the dissociation of CDK9/CYCLIN T1 from HEXIM1, rather than changes in expression levels. To strengthen this conclusion, we also performed an IP followed by a Western Blot experiment with CDK9 antibody, monitoring levels of HEXIM1, CYCLIN T1, and MEPCE following treatment with SR-4835. Our analysis demonstrated that upon treating cells with SR-4835, the association of HEXIM1 and MEPCE with CDK9 was substantially reduced. No effects of SR-4835 were detected on CDK9-CYCLIN T1 interactions (Fig. 4B). Finally, we performed RNA-IP experiments, in which cell lysate from control or SR-4835-treated cells was IP with CDK9 or control IgG antibodies, and the IP fraction was subjected to RT-qPCR with primers for 7SKsnRNA (Fig. 4C). Our analysis shows that indeed following treatment of cells with SR-4835, there was a substantial decrease in 7SKsnRNA enrichment in the CDK9 IP fraction relative to untreated cells, where levels of 7SK snRNA were low and comparable with those that were enriched in the control IgG IP fraction. Overall, these results suggest that targeting of CDK12/CDK13-CCNK by SR-4835 induces the release of CDK9 from the 7SKsnRNP towards activation of gene expression (Fig. 4C).

### Genetic depletion of CDK12, CDK13 or CCNK mimics SR-4835-mediated reactivation of HIV-1 gene expression

**Figure 5:**
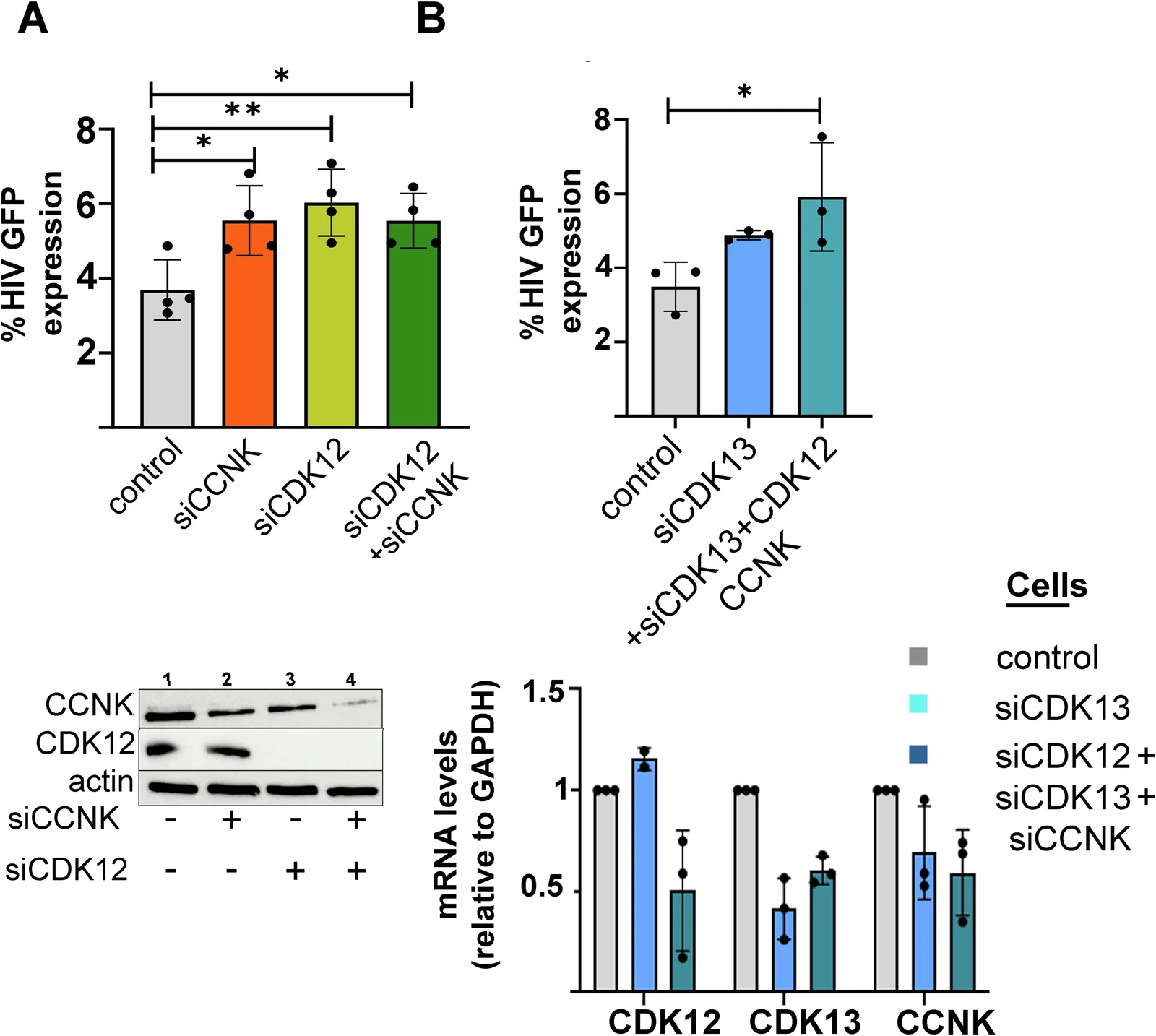
Genetic depletion of CDK12 CDK13 or CCNK mimics SR-4835 treatment and activates HIV-1 gene expression. (**A**) Depletion of CDK12 and CCNK expression mimics SR-4835 mediated HIV-1 gene reactivation. 2D10 cells were electroporated with siRNAs specifically targeting CCNK (siCCNK), CDK12 (siCDK12), or both (total concentration 100 nM). Non-targeting siRNA served as a control. 48 h post-treated cells were monitored by FACS for HIV-1 GFP expression. Lower panel presents western blot analysis confirming CCNK or CDK12 depletion of expression, where actin levels served as a loading control. One-way ANOVA analysis – Tukey’s multiple comparisons test. *P = 0.0340: siNC vs siCCNK; **P = 0.0051: siNC vs siCDK12; *P = 0.0222: siNC vs siCCNK+siCDK12. (**B**) Depletion of CDK13 and CCNK expression mimics SR-4835 HIV-1 gene activation. 2D10 cells were electroporated with siRNAs specifically targeting CDK13 or CDK12, CDK13 and CCNK (siCDK12/CDK13/CCNK; total concentration 100 nM). Non-targeting siRNA served as a control. 48 h post treatment cells were monitored by FACS for HIV-1-GFP expression. The lower panel presents RT-qPCR confirming CDK13, CDK12, and CCNK depletion. Data were analyzed by one-way ANOVA and Tukey’s multiple comparisons test. ns = 0.1935: siNC vs siCDK13 & *P = 0.0329: siNC vs siCDK12+siCCNK+siCDK13. Data are presented as mean ± SD (*n* = 3 independent experiments).

To confirm that targeting of CDK12/CDK13-CCNK with SR-4835 reverses HIV-1 latency, we employed a genetic approach and depleted either CDK12 or CCNK expression in 2D10 cells upon electroporation of siRNAs (Neon NXT system) (Fig. 5A). Efficient knockdown of CDK12, or CCNK expression was confirmed by Western Blotting or by monitoring mRNA levels by qPCR and compared to cells transfected with negative control non-specific siRNA (Fig. 5B). Our results indicate that that upon depletion of CDK12 or CCNK, HIV-1 gene expression was minimally induced (x1.5 fold), compared to non-targeted cells. Interestingly, combined knockdown of both CDK12 and CCNK expression did not increase these effects on HIV-1 gene expression, suggesting that loss of each component is sufficient to slightly reactivate HIV gene expression (Fig. 5A). Similar experiments were performed to examine effects of CDK13 depletion with specific siRNA (Fig. 5B). Depletion of CDK13 alone induced minimal HIV-1 gene expression (x1.4 fold), while depleting CDK12/CDK13 and CCNK together further activated HIV-1 gene expression (x2 fold relative to control; Fig. 5B).

**Figure EV2:**
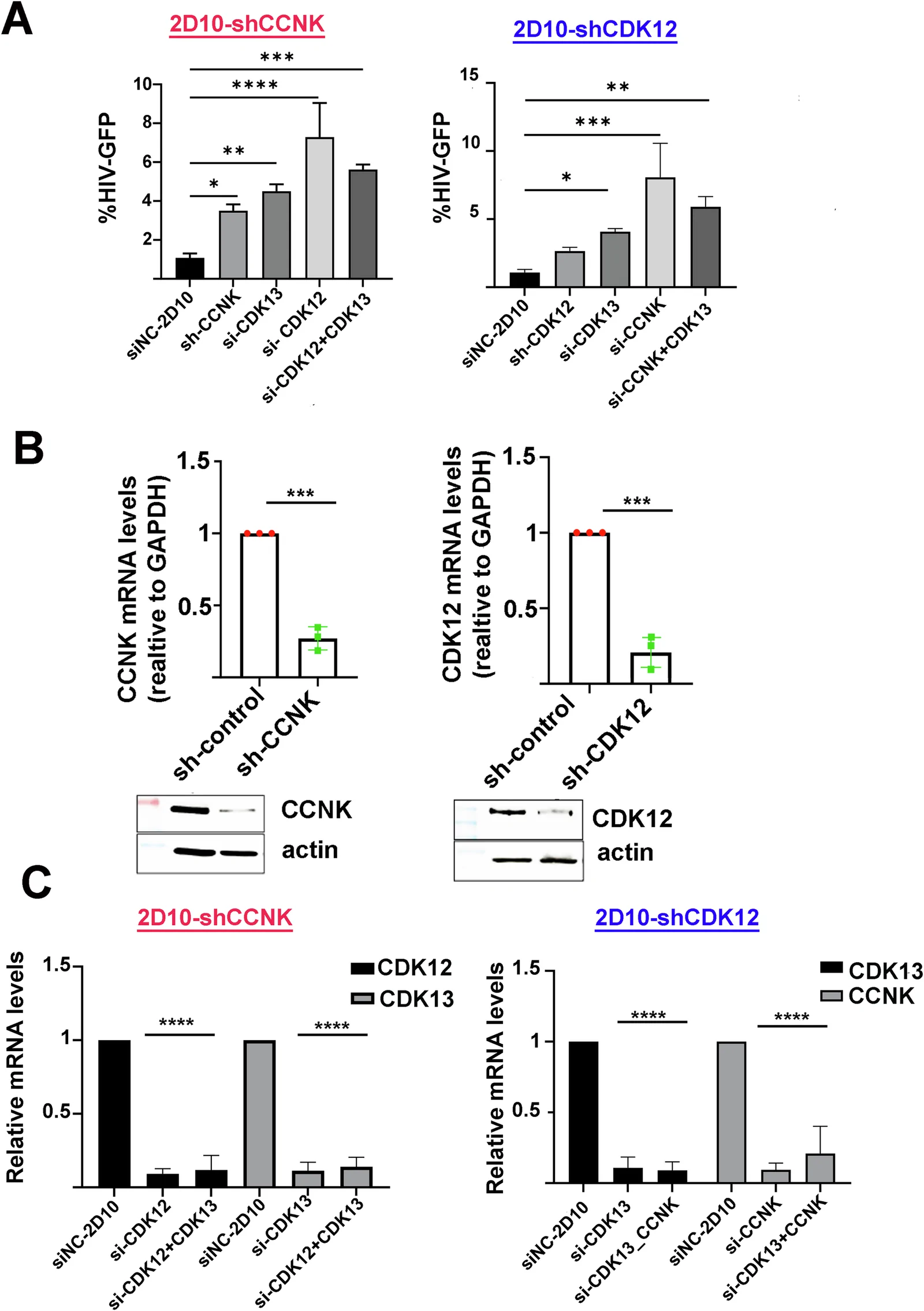
Genetic Targeting of CDK12/CDK13-CCNK mimics SR-4835-mediated HIV-1 activation. (**A**) 2D10 CD4 T cells were transduced with lentivirus expressing shRNA targeting CCNK or CDK12. Cells were selected with puromycin to achieve stable knockdown of CCNK or CDK12. Stable cells were then electroporated with the indicated siRNA (total concentration 100 nM). 48 h post electroporation, HIV-1-GFP expression was monitored by FACS to document HIV-1 gene activation. Data are presented as mean ± SD from *n* = 3 independent biological replicates. Statistical significance was determined using one-way ANOVA. Left Panel: *P* = 0.0172: siNC vs shCCNK, *P* = 0.0011: siNC vs siCDK13, *P* < 0.0001: siNC vs siCDK12 and *P* = 0.0002: siNC vs siCDK12+siCDK13. Right Panel: *P* = 0.0446: siNC vs siCDK13, *P* = 0.0001: siNC vs siCCNK and *P* = 0.0020: siNC vs siCDK13+siCCNK. (**B**) qPCR analysis and western blot confirm CCNK or CDK12 depletion. Relative mRNA expression of CDK12 and CCNK was quantified by RT-qPCR in stable shCCNK-2D10 or shCDK12-2D10 clonal cell lines. Transcript levels are normalized to the control 2D10 which was set to 1. Data are presented as mean ± SD from three independent biological replicates. Statistical significance was determined using two-way ANOVA. ****P = 0.0001. (**C**) qPCR confirm siRNA-specific knockdown of CCNK, CDK12, CDK13 or combination in clonal sh-CDK12 or sh-CCNK cells. Data are presented as mean ± SD from *n* = 3 independent biological replicates. Statistical significance was determined using One-way ANOVA. ****P = 0.0001.

We also monitored HIV-1 gene expression in 2D10 T cells where CDK12 or CCNK was stably depleted via lentivirus-mediated shRNA knockdown (Supplementary Fig. EV2). Stable depletion of CCNK expression led to a 4-fold activation of HIV-1 gene expression relative to control cells. Interestingly, additional depletion of CDK13 in these CCNK-depleted cells slightly induced HIV-1 gene expression (1.2-fold relative to CCNK depletion alone; or 5-fold relative to control cells; Supplementary Fig. EV2A). Furthermore, depletion of CDK12 expression in CCNK-depleted cells enhanced HIV-1 gene activation 2-fold (8-fold relative to control cells). Finally, introducing both CDK12 and CDK13 siRNAs into CCNK-depleted cells induced HIV-1 gene activation 1.5-fold relative to CCNK-depleted cells, representing a 5-fold increase relative to control cells (Supplementary Fig. EV2A).

The effects on HIV-1 gene expression in Jurkat 2D10 T cells were also monitored following stable depletion of CDK12 via shRNA (supplementary; Fig. EV2B). Depletion of CDK12 alone slightly enhanced HIV-1 gene expression by approximately 2-fold. Additional depletion of CCNK by siRNA in these CDK12-depleted cells activated HIV-1 gene expression ~8-fold relative to control cells (a 4-fold increase relative to CDK12-depleted cells). Meanwhile, introducing CDK13 siRNA slightly activated HIV-1 gene expression (1.5-fold relative to CDK12-depleted cells; or 4-fold relative to control cells). Introducing siRNAs for both CDK13 and CCNK into CDK12-depleted cells further induced HIV-1 gene activation 1.6-fold relative to CDK12-depleted cells, corresponding to a 5-fold increase relative to control cells (supplementary; Fig. EV2B). Together, these findings confirm that depending on the levels of depletion, the combined genetic inhibition of CDK12, CDK13, and CCNK slightly induces the reactivation of HIV-1 infection.

### Targeting CDK12/CDK13-CCNK with SR-4835 reactivates HIV-1 transcription from primary CD4 + T cells under cART and from cells derived from PLWH

**Figure EV3:**
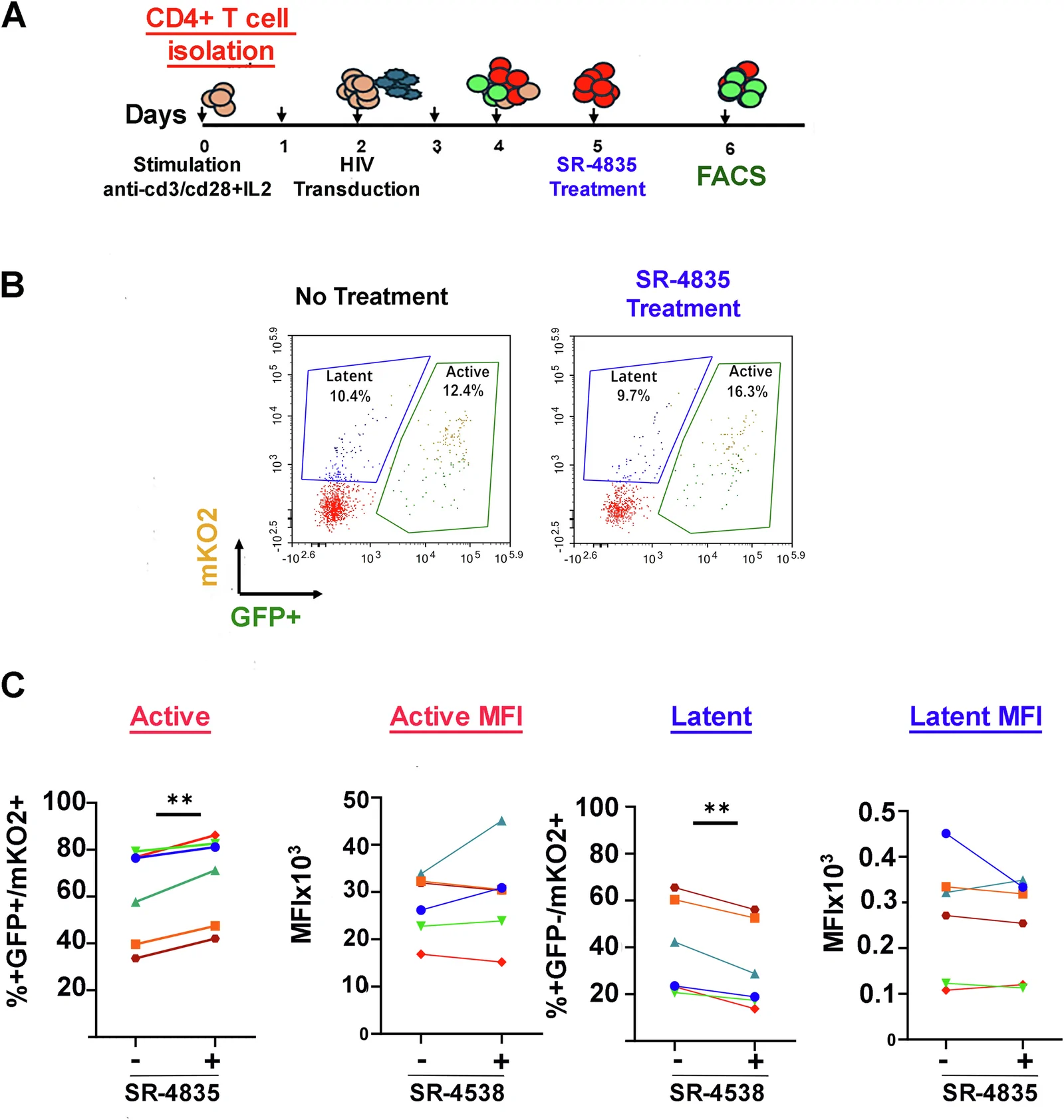
SR-4835 CDK12/CDK13-CCNK inhibitor reverses HIV-1 latency in primary CD4 + T cells. (**A**) Schematic showing experimental design for isolation, activation, HIV-1_GKO_ transduction and SR-4835 treatment of primary CD4 + T cells. (**B**) SR-4835 treatment increases the proportion of GFP⁺ cells, indicating reactivation of latent HIV-1. Representative FACS plots show identification and characterization of latent (GFP-/mKO2⁺) and active (GFP + /mKO2⁺) HIV-1 in primary cell populations in the presence or absence of SR-4835. (**C**) Quantification of HIV-1 reactivation in primary CD4 + T cells shown as the percentage of active (GFP⁺/mKO2⁺) cells or (latent (GFP-/mKO2⁺) HIV-1 within the infected population (MKO2+ cells), along with the corresponding GFP mean fluorescence intensity (MFI), reflecting the level of HIV-1 transcription per active cell. Primary CD4⁺ T cells from independent donors (*n* = 6) were analyzed across treatment conditions. Each dot represents an individual donor, with paired samples connected by lines. Data were normalized to the mKO2⁺ population, and statistical significance was determined using a paired Student’s t-test (**P = 0.003).

**Figure 6:**
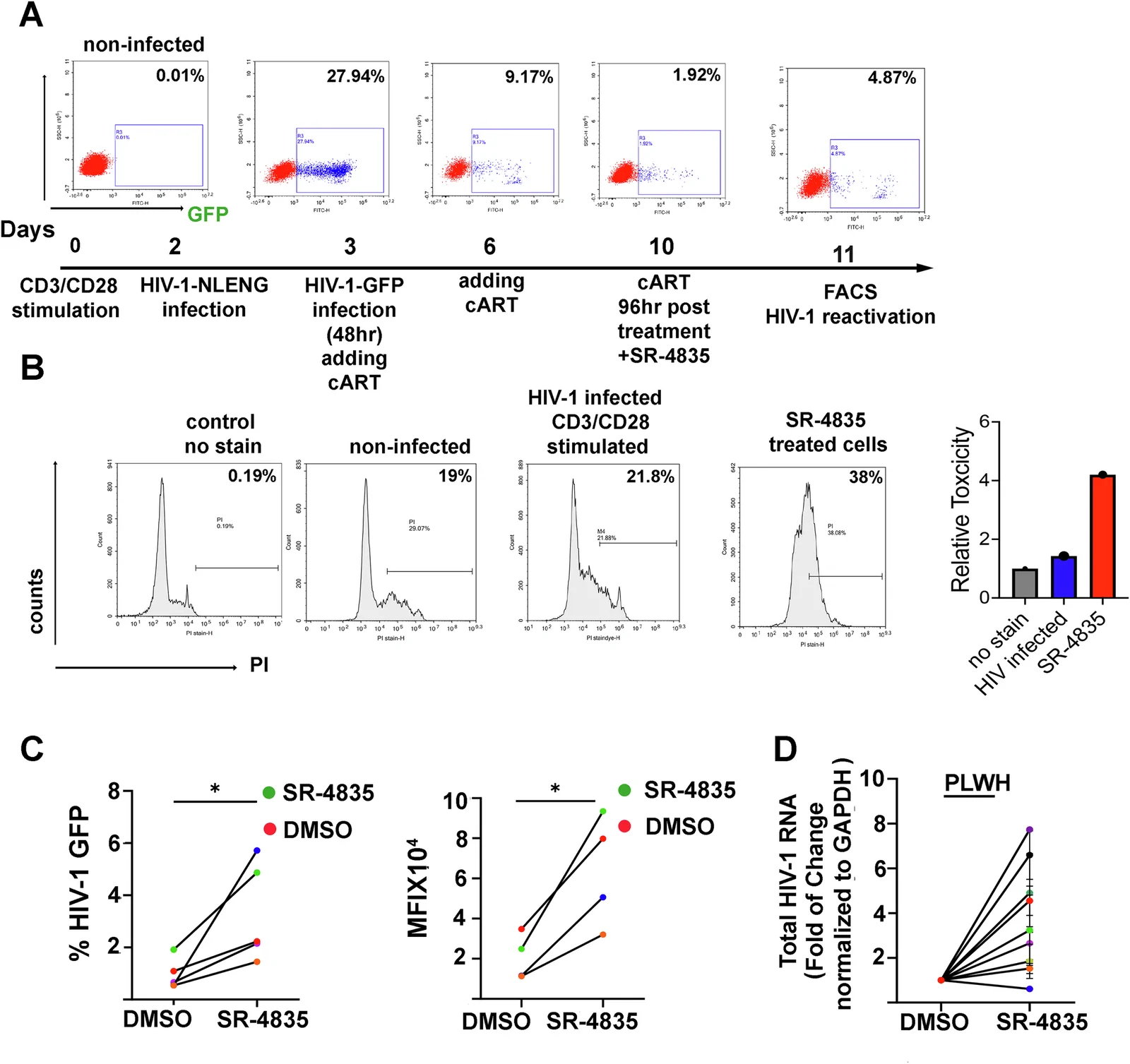
SR-4835 targeting of CDK12/CDK13-CCNK reverses HIV-1 latency in primary CD4 + T cells and in cells derived from PLWH under therapy. (**A**) Treatment of primary CD4 + T cells under cART with SR-4835 reverses HIV-1 latency. CD4 + T cells were isolated from PBMCs, stimulated, and transduced with HIV-1-NLENG, a replication-competent HIV-1 expressing GFP. Infected cells were then subjected to cART for the indicated time points, and at day 6 post-purification, cells were treated with SR-4835 for 2 h. Cells were washed and kept growing for an additional 7 days (Day 11), at which HIV-1 infection was monitored by FACS, documenting reactivation of HIV-1-GFP mediated by SR-4835. Presented are FACS dot blots at the indicated time points of the experimental design (below). (**B**) SR-4835 presents minimal toxicity on primary CD4 T cells. FACS dot blots and quantification of SR-4835 toxicity by PI staining. Also presented is a quantitative analysis of the FACS histograms. (**C**) SR-4835 induces HIV reactivation in primary CD4 T cells under cART. Percentage of HIV-GFP-positive cells in four donor samples under cART that were treated with DMSO (control) or SR-4835. Each line represents an independent donor. SR-4835 shows an increased HIV reactivation compared with DMSO (*P = 0.0218). Also shown are the mean fluorescence intensity (MFI) of the HIV-GFP signal (×10⁴) in DMSO and SR-4835-treated conditions (*P = 0.0424). Each colored line represents a paired donor. SR-4835 treatment significantly increases GFP intensity. (**D**) PBMCs isolated from PLWH under therapy (*n* = 9) were thawed and treated with either DMSO or SR-4835 for targeting CDK12/CDK13-CCNK for 2 h. Cells were then washed and kept growing at optimal growth conditions for additional 72 h. RNA was extracted from cells and subjected to qPCR analysis with the indicated primers for measuring full-length viral RNA normalized to GAPDH expression. Dots represent results obtained for cells from individual donors. Shown are the means +/− s.d. of triplicate measurements (Statistical analysis was performed by a paired t-test; *P* = 0.0078).

We next shifted our experiments to monitor the effects of SR-4835 on primary CD4 T cells, which serve as the major reservoir for latent HIV-1. Naïve CD4 T cells were isolated from six donors (both male and female) using the RosetteSep™ protocol. Isolated primary cells were stimulated and transduced with a single-round VSV-G pseudotype HIV-1_GKO_. This virus allows for the simultaneous measurement of both active (GFP + /mKO +) and latent (GFP-/mKO +) HIV-1. Following transduction, cells were treated with SR-4835 for 2 h, washed and grown for an additional 48 h before monitoring HIV-1 gene expression by FACS (supplementary; Fig. EV3A). Our results demonstrate that treating primary cells with SR-4835 shifted the equilibrium between active and latent HIV-1 towards the activated state (supplementary; Fig. EV3B). This shift was manifested both as an increase in the percentage of cells with reactivated latent HIV-1, and as elevated mean fluorescence intensity (MFI) values, which correspond to heightened transcriptional activity (supplementary; Fig. EV3C). In addition, we monitored the effects of SR-4835 on reactivation of primary CD4 T cells that were treated with antiretroviral therapy (cART), mimicking the scenario of people living with HIV-1 (PLWH) under therapy (Fig. 6). Isolated CD4+ cells were stimulated with CD3/CD28 beads, and cells were then transduced with the HIV-1-NLENG replication-competent virus. Following transduction, cells were treated with the following retroviral inhibitors: Raltegravir (300 nM), Efavirenz (300 nM), and Lenacapavir (100 nM), during which suppression of HIV-1 replication was confirmed by FACS over a week time frame. Cells were then treated with SR-4835 (0.5 µM) for 2 h, washed, and kept growing for an additional 24 h. HIV-1 reactivation by SR-4835 was assessed one day later by FACS (Day 11) (Fig. 6A). Our analysis revealed minimal reactivation of HIV-1 gene expression upon SR-4835 treatment (x2.5 fold), compared to the untreated control (Fig. 6C). Treatment of cells with SR-4835 showed relatively low toxicity, as indicated by PI staining (Fig. 6B).

Effects of SR-4835 on HIV-1 latency reversal were also tested in PBMCs derived from nine PLWH under antiretroviral treatment suppressing virus replication. Available information on those analyzed samples is presented in Table EV1. Patient-derived cells were treated with either DMSO or SR-4835 for 2 h and then kept under optimal growth conditions for an additional 72 h. Next, RNA was extracted from cell samples and analyzed by qPCR with primers detecting full-length viral RNA (see Methods for experimental details and primer sequences). Our analysis demonstrates that short-term treatment with SR-4835 was sufficient to reverse HIV-1 latency in PBMCs of most (8 out of 9) donors (Fig. 6D). Although reactivation was broadly observed, the magnitude of HIV-1 gene reactivation varied substantially between PBMCs from different donors. SR-4835-mediated CDK12/CDK13 inhibition over a short time frame can therefore induce HIV gene expression in PBMCs from PLWH but reactivation efficacies are heterogeneous.

**Figure EV4:**
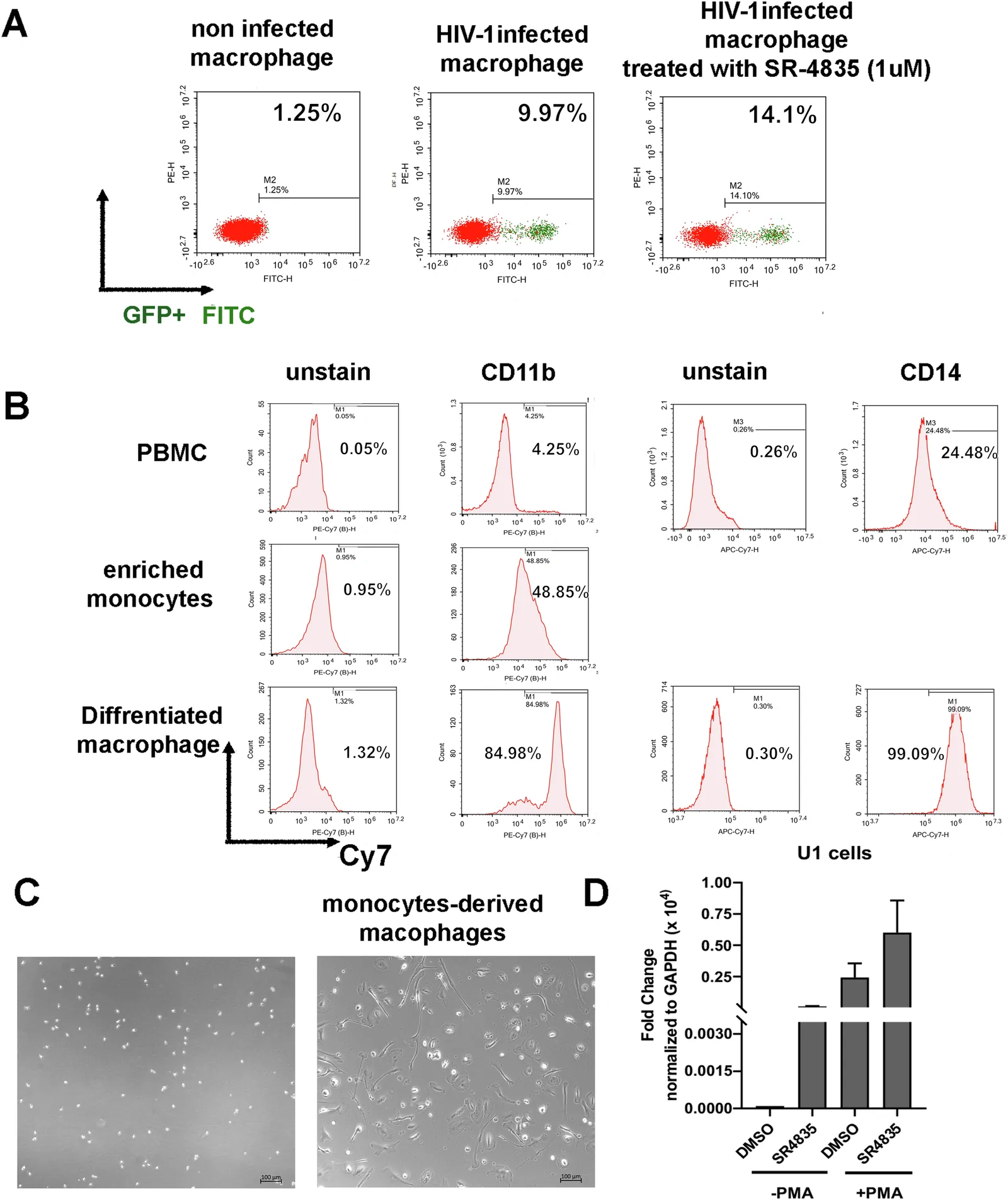
Effects of SR-4835-mediated inhibition of CDK12/CDK13 on human primary monocyte derived macrophages. (**A**) Monocytes were isolated from PBMCs using the “EasySep enrichment kit” (Stemcell Technologies). Cells were differentiated for 5 days with GM-CSF and then stained with aCD11b PE-Cy7 conjugated antibody (Bio-Legend) for characterization. 24 h post stimulation, cells were transduced with HIV-1 NLENG at low MOI. At day 6 post stimulation, cells were treated with 0.5 mM SR-4835 for 2 h and washed before being analyzed by FACS for HIV-1 GFP expression. (**B**) Cells were stained with CD11 b or CD14 by FACS for characterizing monocyte-derived macrophages. (**C**) Monocyte-derived macrophages exhibit a distinct phenotype. Monocytes were isolated from human PBMCs using “EasySep enrichment cocktail” (Stem Cells Technology) and stimulated with 50 ng/ml of GM-CSF (Miltenyi Biotec) for 8 days before imaging by light microscope. (**D**) U1 human monocytes cells that are infected with HIV-1 were cultured under standard conditions and treated with the indicated concentrations of SR-4835 (0.5 mM) in either the absence or presence of PMA/Ionomycin (200 nM/1 mM) or 48 h (*n* = 3 independent biological replicates. Following treatment, total RNA was extracted using an RNA isolation kit according to the manufacturer’s instructions. Equal amounts of RNA were reverse transcribed into cDNA using a reverse transcription kit. Quantitative PCR was performed using gene-specific primers targeting the HIV-1 unspliced transcripts. Relative HIV transcript levels were calculated using the 2^−ΔΔCt^ method and expressed as fold change relative to GAPDH and to the DMSO-treated control. Data are presented as mean ± SD.

We also tested the activation effects of SR-4835 on human monocyte-derived primary macrophages, which are also a main cell reservoir of latent HIV-1. Monocytes were isolated from PBMCs using “EasySep enrichment kit”. Cells were differentiated for 5 days with GM-CSF and then stained with CD11b or CD14 for characterization. 24 h post-stimulation, cells were infected with HIV-1 NLENG at low MOI, and at day 6 post-stimulation, cells were treated with 0.5 μM SR-4835 for 2 h and washed before being analyzed by FACS for HIV-1 GFP expression (supplementary; Fig. EV4). Our results show that HIV-1 gene expression was reactivated in macrophages upon treating cells with SR-4835, implying a general effect that is not cell-specific. However, the fact that the effects of SR-4835 in macrophages were subtle may point to other restrictions on HIV-1 gene expression that are specific to macrophages (supplementary; Fig. EV4B,C). Finally, we also tested the effects of SR-4835 on human monocytes U1 cells that are infected with HIV-1 and carry a transcriptionally repressed provirus due to a point mutation in Tat. Our analysis confirmed that indeed, treatment of U1 monocytes with SR-4835 reactivates HIV-1 gene expression (supplementary; Fig. EV4D).

### Targeting CDK12/CDK13-CCNK induces the phosphorylation of Pol II and the recruitment of CDK9 and histone activation marks to the HIV-1 promoter and to global gene promoters

**Figure 7:**
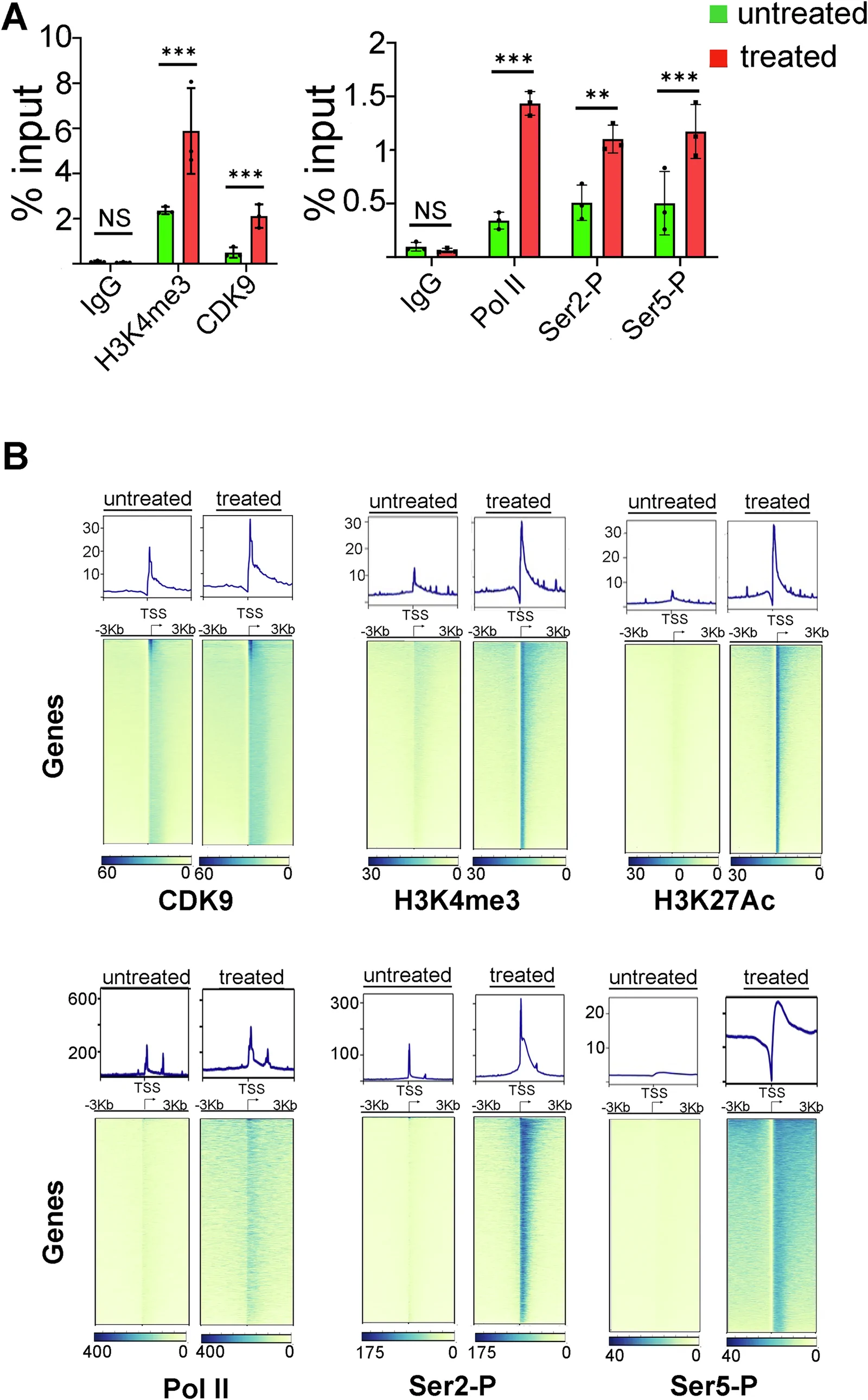
Targeting CDK12/CDK13-CCNK induces the phosphorylation of Pol II and the recruitment of CDK9 and histone activation marks to the HIV-1 promoter and to global gene promoters. (**A**) ChIP-qPCR analysis of Jurkat HIV-1-transduced T cells treated with SR-4835 for 2 h, washed, and harvested 24 h later, before being subjected to ChIP-qPCR. ChIP was performed on SR-4835-treated or untreated cells using the indicated antibodies. Data is presented as percentage of input relative to IgG as a negative control. Statistical significance is based on calculating mean ± SD from three independent experiments using Two-way ANOVA analysis. Left Panel: *P* = 0.0006: H3K4me3; *P* = 0.001: CDK9; Right Panel: *P* < 0.0001: RNA Pol II; *P* = 0.0017: RNA Pol II Ser 2; *P* = 0.0005: RNA Pol II Ser5. (**B**) CUT&RUN analysis shows global increases in gene promoters and gene bodies of CDK9 total RNA Pol II and well-specific CTD Ser2-P and Ser5-P levels following SR-4835 treatment. Experiments were performed as described in the Methods based on Epicypher protocols.

Our results show that CDK9 is released from 7SKsnRNP upon treatment of cells with SR-4835 and targeting CDK12/CDK13-CCNK, potentially inducing transcriptional activation from the HIV-1 promoter, which is mediated by P-TEFb. To further confirm these results, we performed chromatin immunoprecipitation (ChIP) qPCR assays, monitoring levels of histone activation marks, as well as the occupancy of CDK9 and Pol II on the HIV-1 promoter in Jurkat cells infected with HIV-1_GKO_. Cells were treated with SR-4835 for 2 h, and chromatin was isolated 24 h later and precipitated with antibodies targeting H3K4me3 (epigenetic mark for active promoters), Pol II (total), CDK9, as well as for Ser2P and Ser5P that, respectively, mark initiation or elongation of transcription. Our data revealed that upon treatment of cells with SR-4835, higher levels of H3K4me3 were detected on the HIV-1 promoter (x6-fold), indicating a transcriptionally active environment at the viral promoter that follows CDK12/CDK13-CCNK targeting (Fig. 7A). Similar results were obtained for CDK9 (x2.2-fold increase) upon SR-4835 treatment. These results are consistent with the above observations of released P-TEFb from 7SKsnRNP and recruitment of the free complex to the HIV-1 promoter to activate HIV-1 gene expression (Fig. 4). A similar increase upon SR-4835 treatment was observed in levels of total Pol II (x4.7-fold); Ser2-P (x2 fold); and Ser5-P (x2 fold) (Fig. 7A). These results confirm that targeting CDK12/CDK13-CCNK by SR-4835 induces HIV-1 gene expression, accompanied with CDK9 recruitment and elevated levels of Pol II phosphorylation at Ser2 and Ser5, thereby monitoring Pol II pause-release and elongation of HIV-1 gene expression.

To explore genome-wide effects of SR-4835, we conducted CUT&RUN analysis in SR-4835-treated Jurkat T cells. We observed elevated genome-wide CDK9 occupancy at TSS and gene bodies (Fig. 7B). Additional global CUT&RUN analysis documented elevated levels at TSS and gene bodies of H3K4me3 and H3K7ac histone activation marks, as well as Pol II, Ser2P and Ser5P, indicating enhanced transcriptional initiation and elongation activity.

### Targeting CDK12/CDK13-CCNK alters global gene expression program

**Figure EV5:**
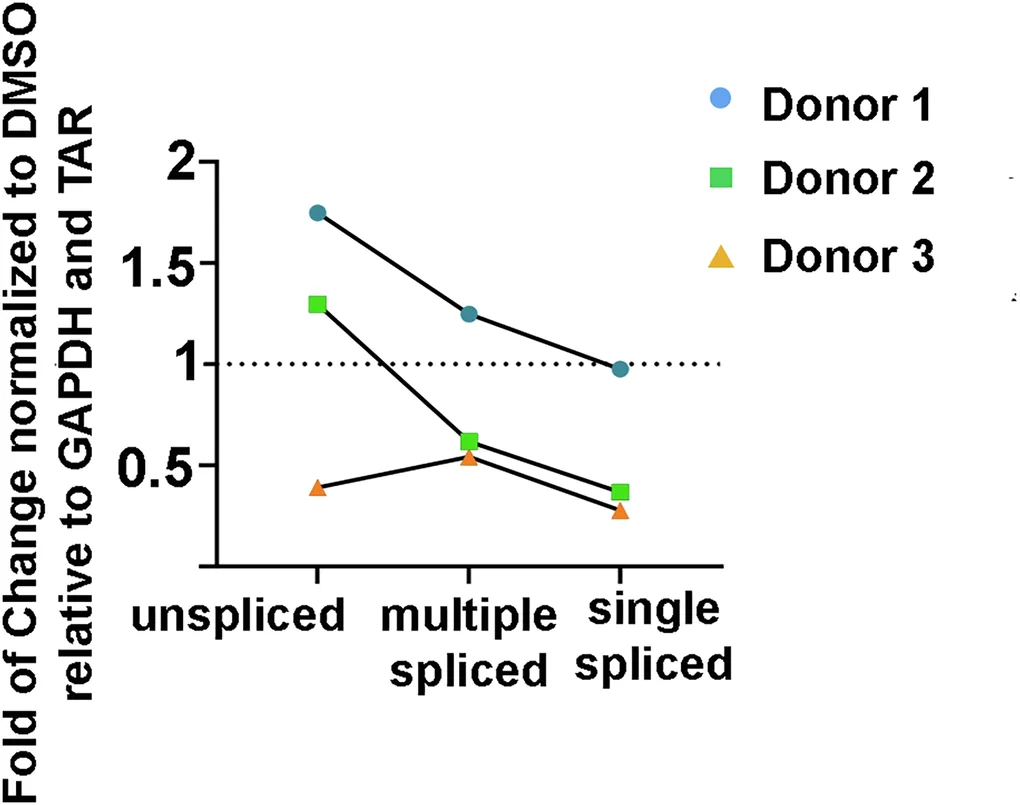
SR-4835 CDK12/CDK13-CCNK SR-4835 inhibitor has minimal effects on HIV-1 gene splicing. Primary CD4 T cells were isolated from naïve PBMCs from 3 donors and treated with SR-4835 (0.5 μM) for 2 h and washed. 48 h later, cells were harvested, and total RNA was isolated. The expression levels of HIV Gag (unspliced), Tat (multiply spliced), and Env transcripts (single spliced) were analyzed by RT qPCR using specific primers. Data are presented as fold of change of mRNA normalized to DMSO and relative to GAPDH and TAR, indicating changes in transcript splicing balance upon SR-4835 treatment. Data are shown as fold change from independent donors. The dashed line represents cells treated with DMSO as a control.

CDK12/CDK13-CCNK play pivotal roles in regulating the global metazoan gene expression program. To determine if SR-4835 treatment activates HIV-1 gene expression by affecting alternative splicing of viral mRNA, we performed a PCR-based splicing assay, monitoring the ratios between full-length unspliced viral RNA (vRNA; Gag), single-spliced vRNA (Env), and multiple spliced (Tat) HIV-1 viral RNA species (Cochrane, 2014). HIV-1 splicing is tightly regulated during latency, and previous results indicate that unspliced HIV-1 RNA species are more abundant than spliced HIV-1 viral RNA species (Pasternak and Berkhout, 2021). Furthermore, CDK13 is known to play a role in RNA splicing. Upon targeting CDK12/CDK13-CCNK with SR-4835, we observed an overall reduction in splicing, at least at two of three donors, pointing to limited effects of SR-4835 (supplementary; Fig. EV5).

**Figure 8:**
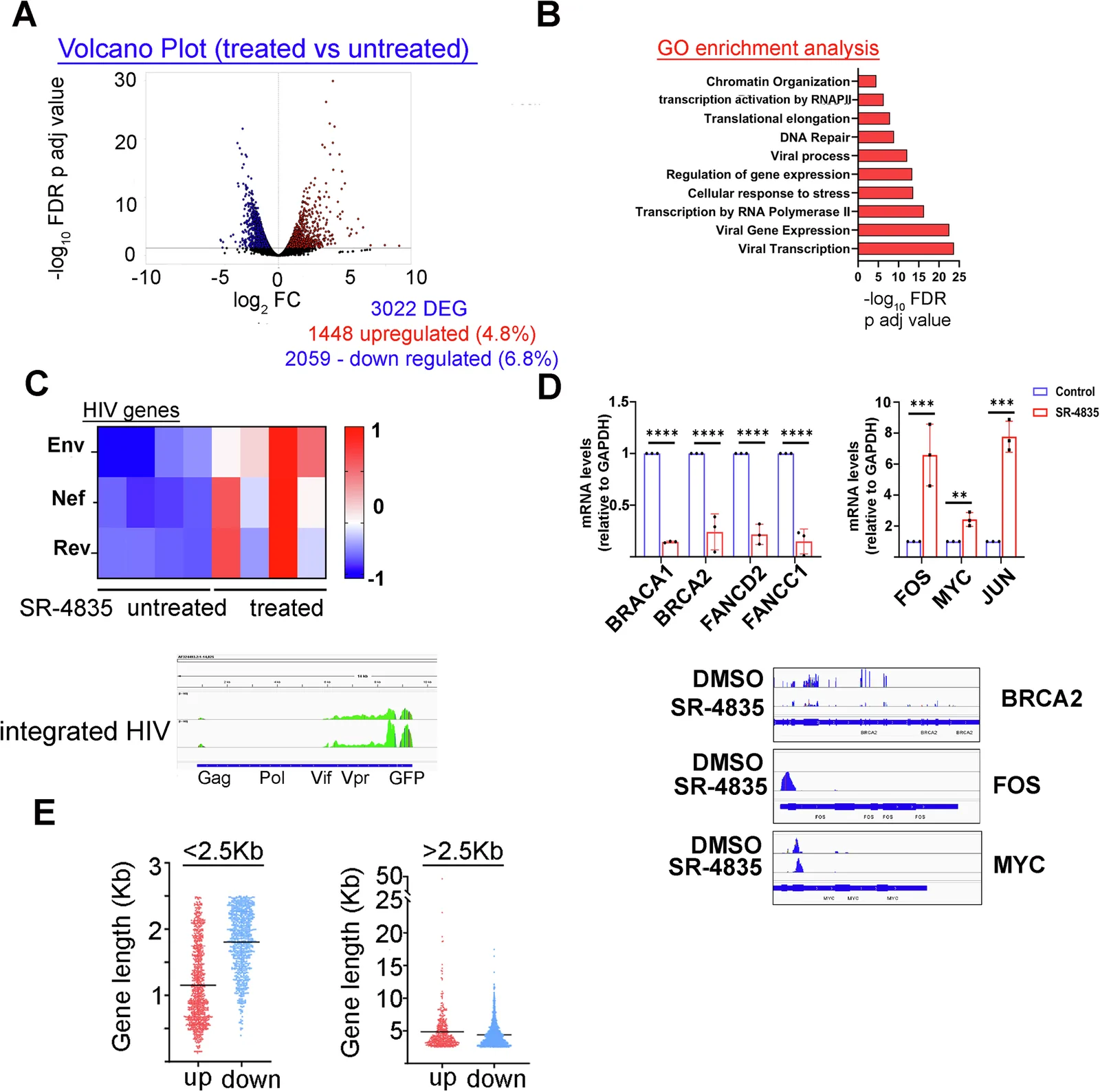
CDK12/CDK13-CCNK targeting induces global changes in gene expression. (**A**) A volcano plot showing differentially expressed genes between SR-4835-treated and untreated cells (*n* = 3 biological replicates per group). Red and blue dots represent significantly upregulated and downregulated genes (*n* = 3, Log_2_FC > 1, padj < 0.05), respectively. (**B**) Gene Ontology enrichment analysis highlighting biological processes and pathways that are significantly modulated following targeting of CDK12/CCNK by SR-4835. (**C**) Heatmap based on Z-score, showing differential expression of HIV-1 genes from the RNA-Seq transcriptomic dataset. GFP replaces the NEF downstream viral gene. (**D**) DNA damage repair-related genes are downregulated upon SR-4835 treatment, while P-TEFb-target genes show increased RNA levels in SR-4835-treated cells. Control cells were untreated. Corresponding IGV peaks are also described below. Data represent mean ± SD from three independent experiments. Statistical significance was assessed as two-way ANOVA analysis, Sidak’s multiple comparisons test. ****P = 0.0001 (BRCA1, BRCA2, FANCD2 and FANCi) and ***P = 0.001(FOS and JUN); **P = 0.0043 (MYC). (**E**) Analysis of gene size based on the RNA-Seq analysis in cells treated with SR-4835. Long transcripts are defined as >2.5 kb, while shorter ones are <2.5 kb.

To investigate the genome-wide transcriptome profile following SR-4835 inhibition, we performed RNA-Seq in Jurkat T cells infected with HIV-1. Our analysis identified 30,226 DEGs (adjusted *p* < 0.05) that were upregulated upon SR-4835 treatment relative to the control untreated cells. Among these DEGs, there was a relatively similar change between up- and down-regulated genes following SR-4835 treatment. We identified 1448 (4.8%) upregulated genes and 2059 (6.8%) down-regulated genes (Fig. 8A). Gene Ontology (GO) enrichment analysis identified significant enrichment of pathways associated with transcriptional regulation and activation, stress response and cell proliferation, while pathways related to DNA repair and replication were suppressed (Fig. 8B). To visualize the expression patterns of the integrated HIV-1, we generated a heatmap of significantly altered viral genes following SR-4835 treatment, confirming increased expression of downstream HIV-1 genes, specifically GFP, which is inserted downstream instead of the *nef* gene (Fig. 8C). Integrative Genomics Viewer (IGV) images confirmed the elevated reads that correspond to the HIV-GFP downstream loci. Cellularly, key proto-oncogenes such as *MYC*, *FOS* and *JUN* were also significantly upregulated upon CDK12/CCNK targeting, while DNA damages response (DDR) pathways including genes like *BRCA1*, *FANCD2*, and *FANCI* were downregulated (Curti et al, 2024; Pitolli et al, 2023; Wang et al, 2023) (Fig. 8D). These results confirm that CDK12/DK13-CCNK targeting suppresses DDR gene expression, while enhances transcriptional programs associated with cellular stress and cell proliferation. Interestingly, based on the analysis, we found that following treatment with SR-4835, shorter transcripts were significantly enriched in the upregulated genes relative to downregulated transcripts. In upregulated genes, those that were below 2.5 kb (<2.5 kb) consisted of 66.85% (968 genes), relative to 33.1% of the long transcripts (> 2.5 kb; 480 genes); while in the downregulated genes, there was no significance difference between the short or long transcripts 37.5% (774 genes) versus relative to long transcripts (62.5% = 1286) (Fig. 8E).

## Discussion

In this work we employed a pharmacological approach to specifically target CDK12/CDK13-CCNK and deplete their expression through degradation by treating cells with SR-4835. Our results confirm that treatment of cells with SR-4835 induces CCNK degradation and CDK12 depletion with minimal toxicity or apoptosis. Moreover, targeting CDK12/CDK13-CCNK expression by SR-4835 induces a dual and broad cellular gene expression program, defined by marked downregulation of gene sets, but surprisingly, induction of specific pathways and cellular genes. This gene activation program is manifested with an increase in genome-wide occupancy levels of Pol II, H3K4me3, and H3K27Ac activation histone marks around TSS as well as within gene bodies, which is accompanied with increased levels of CDK9, Ser5P, and Ser2P of the Pol II CTD. Our results coincide with a recent unpublished work by Wang et al, showing a similar transcriptomic profile upon targeting CDK12 (Wang et al, 2025). However, our study adds to this work by employing a higher-affinity and more specific inhibitor for CDK12/CDK13-CCNK, and demonstrating that for certain genes, primarily the short ones, there is an elevated activation accompanied by increased levels of histone activation markers and Pol II levels. We further show that upon CDK12/CDK13-CCNK targeting, this specific gene activation program is driven by P-TEFb. Indeed, molecular characterization of the equilibrium of P-TEFb within cells indicates that upon treating cells with SR-4835 and depletion of CDK12/CDK13-CCNK, there is a shift of P-TEFb into its free configuration, releasing it from the inhibitory 7SKsnRNP. To validate the requirement of CDK9 kinase activity for SR-4835-mediated gene activation, we applied two CDK9 inhibitors, Flavopiridol and AZD-4573, and confirmed the dependency of SR-4835-mediated gene activation on P-TEFb/CDK9. Transcriptomic cellular profiling of genes that are differentially expressed upon CDK12/CDK13-CCNK targeting, further demonstrates that there is a significant enrichment of cellular pathways that are associated with transcriptional activation, stress response and cell proliferation, while pathways related to DNA repair and replication, which are regulated by CDK12, are suppressed. Notably, key P-TEFb-regulated proto-oncogenes such as MYC, FOS, and JUN, as well as heat shock protein (HSP70) are significantly upregulated relative to control untreated cells, while multiple DNA damage response (DDR) genes, including BRCA1, FANCD2, and FANCI, are strongly downregulated (Curti et al, 2024; Pitolli et al, 2023; Wang et al, 2023). Interestingly, based on the RNA-Seq analysis, treatment with SR-4835 results in a significant enrichment of short transcripts relative to longer ones, coinciding with results from Wang et al (2025). These data highlight the key role of CDK12/CDK13 in regulating an early transcriptional elongation checkpoint, where CDK12 targeting induces a premature termination and RNA decay that is disproportionally apparent in long genes; while short genes show increased production of full-length transcripts. One of the notable short genes that were highly induced was MYC, known to induce potent pleiotropic effects and apoptotic cell death upon targeting CDK12/CDK13 and CCNK. MYC gene expression following CDK12/CCNK inactivation may contribute to the specific killing of malignant cells, turning CDK12 a potentially therapeutic target for treating human malignancies, where CDK12 is mutated or overexpressed, such as HER2+ malignancies. Nevertheless, the therapeutic benefits of CDK12/CDK13-CCNK targeting will require substantial clinical investigation, given that those tumors readily develop resistance to multiple lines of other targeted therapies. The fact that P-TEFb is activated upon CDK12/CDK13-CCNK targeting and reshapes a transcriptional response that promotes survival of stressed cells strengthens the therapeutic benefit of such a CDK12-CDK9 axis (Bugai et al, 2019). The benefit of such a CDK12/CDK13-P-TEFb axis for treating cancer has been reported by Wang et al, demonstrating a broad gene activation effect, which depends on P-TEFb, including those genes encoding for AP-1 and NF-kB pathways in BRAF-mutated melanoma cells (Houles et al, 2023; Houles et al, 2022) and genes driven by p53 and NF-κB (Wang et al, 2023). The novelty in our work is the understanding that targeting CDK12/CDK13-CCNK drives P-TEFb to substitute CDK12 in activating a specific gene transcription program. This conclusion is further validated for specific upregulated genes, where levels of Pol II and Ser2P are elevated upon targeting CDK12/CDK13-CCNK.

Our discovery that targeting CDK12/CDK13-CCNK reactivates HIV-1 gene expression and reverses latency has potential for clinical translation. Activation of HIV-1 gene expression and latency reversal upon treatment with SR-4835 was demonstrated in several CD4 T cell latency models, in primary human CD4 T cells, and, importantly, in PBMCs from PLWH under ART (Fig. 6D). The magnitude of this reactivation varied between cells from different donors, probably reflecting, e.g., different proviral loads, integration sites of the provirus, and epigenetic constraints. Within the limited set of samples, we were able to analyze herein, SR-4835 responsiveness did not seem to strictly depend on gender, ethnicity or CD4 count (supplementary; Table EV1). Identifying the parameters that govern the response to SR-4835 treatment will constitute an important goal of future studies with cells from larger PLWH cohorts.

In addition, we observed synergistic effects on HIV-1 gene reactivation and latency reversal upon co-treating infected cells with specific LRAs and targeting CDK12/CDK13-CCNK with SR-4835. This is significantly demonstrated for JQ1, but not exclusively, as in some latency cell models, other LRAs like Prostratin, I-BET or SAHA also displayed synergism with SR-4835 treatment. LRAs can be employed as part of a “Shock and Kill” strategy to target HIV-1 infected cells where the provirus is transcriptionally silenced. However, while in vitro LRAs are highly effective, they fail in the clinic (Bendoumou and Van Lint, 2025; Zerbato et al, 2019). We can speculate that an approach where targeting CDK12/CDK13-CCNK, which is already employed for treating human malignancies, can be integrated in current “Shock and Kill” regimens for reactivating latent HIV-1 within the infected reservoir—exposing the virus to efficient killing by the immune response and subsequent immune clearance. Interestingly, SR-4835-mediated HIV-1 reactivation was also observed in primary macrophage-derived monocytes that were infected with HIV-1. However, in this experimental setting, HIV-1 reactivation by SR-4835 was minimal, pointing to additional blocks in macrophages that are not dependent on P-TEFb, and may further inhibit viral reactivation. The efficiency of targeting CDK12/CDK13-CCNK was also confirmed genetically, as knockdown of the expression of CDK12, CDK13, and CCNK induced HIV-1 latency reversal; however, the extent of reactivation was to a lesser extent, presumably due to partial knockdown levels. As targeting CDK12/CDK13-CCNK drives the release of active P-TEFb, and HIV-1 is heavily dependent on P-TEFb-mediated Pol II pause release and elongation of transcription, our results provide a mechanistic pathway for such intervention within the clinic. Our data also set HIV-1 as an optimal system for studying the compensatory rewiring effects of CDK12/CDK13-CCNK and CDK9/CCNT upon targeting CDK12/CDK13-CCNK. Such effects are noted in several cell models for HIV latency, as well as in primary CD4 + T cells that carry a transcriptionally repressed provirus. Low doses of SR-4835 result in HIV-1 activation, accompanied by elevated levels of Pol II, histone activation marks, as well as CDK9, Ser5P, and Ser2P around the HIV-1 promoter. Treating cells with SR-4835 combined with inhibiting the kinase activity of CDK9 with Flavopiridol or AZD-4573 abolishes the ability of SR-4835 to reverse HIV-1 latency.

A question concerning the extent of reservoir activation upon treating cells with SR-4835 is whether all latent reservoirs are activated or only a subset of proviruses. Future work will employ imaging-based approaches to examine activation of integrated HIV-1 provirus at the single-cell level and will further strengthen our conclusions about the extent of viral activation upon targeting CDK12/CDK13.

Our study has a few limitations since it uses a drug that targets CCNK, a subunit of both CDK12 and CDK13. Despite early work on SR-4835 that shows higher affinity of the drug to CDK12 in comparison to CDK13 (Schmitz et al, 2024) is difficult to set apart the effects of SR-4835 on CDK12 or CDK13. Indeed, to date, development of compounds that can distinguish between these closely related kinases and exclusively target one of these kinases has not been reported. Moreover, SR-4835 may have some side effects due to low affinity for other cyclins. Nevertheless, our work uncovers a key and novel interplay between CDK12/CDK13 and CDK9, along with their cyclin partners, CCNK and CCNT. Upon targeting CDK12/CDK13-CCNK, P-TEFb induces gene activation of set of specific genes, predominantly short genes, including HIV-1, while longer genes exhibit elongation defects that ultimately lead to premature termination. Such an interplay ensures a precise and coordinated gene expression program, where genes that are essential for stress response are induced.

## Methods

**Table:** Reagents and tools table

Reagent/Resource	Reference or Source	Identifier or Catalog Number
**Experimental models**		
J-Lat Full Length Cells (Clone 9.2)	American Type Culture Collection (ATCC)	ARP-9848
J-Lat Full Length Cells (Clone 6.3)	American Type Culture Collection (ATCC)	ARP-9846
2D10	Provided by Dr. Jonathan Karn (Case Western Reserve University)	N/A
HEK293T	American Type Culture Collection (ATCC)	CVCL_0063
**Recombinant DNA**		
HIV GKO	Provided by Eric Verdin (Buck Institute for Research on Aging, USA)	N/A
HIV-1 NLENG GFP p6 modified	Prof. Dr. Oliver Keppler	N/A
**Antibodies**		
Anti-rabbit (WB 1:10,000)	Jackson Immuno-research	#168738
Anti-actin (WB 1:10,000)	Sigma	#030M4788
Anti-mouse (WB 1:10,000)	Jackson Immuno-research	#115-035-062
Cyclin T1 (WB 1:1000)	Abcam	ab27963
Cyclin K (WB 1:1000)	Cell Signaling	#19472S
CDK12 (WB 1:10,000)	Abcam	ab317746
Pol II-CTD (CUT&RUN 1:50)	Cell Signaling	#2629S
Pol II-CTD Ser2 (CUT&RUN 1:50)	Cell Signaling	#13499S
Pol II CTD Ser5 (CUT&RUN 1:50)	Cell Signaling	#13523S
CDK9 (WB 1:1000) (CUT&RUN 1:50)	Cell Signaling	#2316S
H3K27ac (CUT&RUN 1:100)	Cell Signaling	#8173S
MEPCE (WB 1:1000)	Abcam	ab185991
HEXIM1 (WB 1:1000)	Abcam	ab25388
Annexin V-Pacific Blue (1:20)	Bio-Legend	#640905
**Oligonucleotides and other sequence-based reagents**		
TriFECTa Kit DsiRNA Integrated DNA Technologies (IDT)	This Study	Table EV1
**Chemicals, Enzymes and other reagents**		
Gibco DMEM	Thermo Fisher Scientific	# 41965039
Gibco RPMI	Thermo Fisher Scientific	# 21875034
PBS	BioWest	# X0515
L-glutamine	BioWest	# X0550
Penicillin – Streptomycin	BioWest	# L0022
Fenozol	A&A Biotechnology	Product code: 203-50
cDNA Synthesis Kit	AzuraQuant	SKU: AZ-2504
LightCycler® 480 SYBR Green I Master	Roche Life Science	Ref: 04887352001
Human GM-CSF	Miltenyi Biotec	#130-093-864
Propidium Iodide	Sigma	#25535-16-4
RNase Inhibitor	New England Biolabs (NEB)	M0307S
Neon NxT Buffer Kit	Invitrogen	Ref N1096R
Acrylamide/Bis-Acrylamide	Bio-Labs	#000142233100
Triton X-100	Sigma	#X100-500mL
Tween 20	Sigma	# P1379
Chemiluminescent Substrate	Cyanagen	Lot PH02A-LB-9
2-Mercaptoethanol	Sigma	#M6250
SR4835	Cayman Chemical	# 2387704-62-1
Lenacapavir	MedChem Express	#2189684-44-2
Efavirenz		
Raltegravir	MedChem Express	#518048-05-0
I-BET 151	Selleck Chemicals	#1300031-49-5
Prostratin	Cayman Chemical	#60857-08-1
SAHA	Cayman Chemical	#149647-78-9
JQ1	Provided by Dr. James E. Bradner	Dana-Farber Cancer Institute
Flavopiridol		
AZD4573	MedChem Express	#2057509-72-3
Dynabeads™ Protein G	Thermo Fisher Scientific	# 10003D
CUT&RUN	EpiCypher	
ChIP DNA Clean & Concentrator	ZYMO Research	#D5206
NEBNext Ultra II DNA Library	New England Biolabs (NEB)	# E7645S
Human IL-2	Peprotech	#200-02-50UG
RosetteSep Human CD4+ Enrichment Cocktail	STEMCELL Technologies	#15022
Human T-Activator CD3/CD28	Thermo Fisher Scientific	#11131D
LSM™ lymphocyte separation medium	MP Biomedicals	SKU: 0850494-CF
EasySep™ Human Monocyte Enrichment Kit	STEMCELL Technologies	#19058
**Software**		
Integrated Genomics Viewer (IGV) version 2.16.1	Broad Institute	https://igv.org/
GraphPad Prism 8.0.2	Graphpad	www.graphpad.com
NovoExpress	Agilent Technologies	www.aceabio.com/novoexpress
**Other**		

siRNA duplex sequences:	
siCCNK_1	Forward – CACUUGGUUCAGCUAAUGUCUGAGA
siCCNK_1	Reverse – UCUCAGACAUUAGCUGAACCAAGUGUA
siCCNK_2	Forward – GAACAAGCCUACUUCAAUAAAUGAA
siCCNK_2	Reverse – UUCAUUUAUUGAAGAAGUAGGCUUGUUCUU
siCDK13_1	Forward – AGCUGAUAGCUUACGAGGAAUATT
siCDK13_1	Reverse – AAUAUUUCCUCGUAAGCUAUCAGCUUC
siCDK13_2	Forward – AGCAGCUAACCUACAGUUGAAGATA
siCDK13_2	Reverse – UAUCUUCAACUGUAGGUUAGCUGCUUA
siCDK9_1	Forward – CUUCAGCUUCUAAAACACGAGAATG
siCDK9_1	Reverse – CAUUCUCGUGUUUUAGAAGCUGAAGGA
siCDK9_2	Forward – GGGACUUGAUUGUCAAGUCAAGUCACUGGA
siCDK9_2	Reverse – UCCAGUGACUUGACAAUCAAGUCCCAA
siCDK12_1	Forward – GUAUUUGAAAGCAACAUCAAAUCTA
siCDK12_1	Reverse – UAGAUUUGAUGUUGCUUUCAAAUACUU
siCDK12_2	Forward – GGCGUCUACGAGAAGAAUUCUCUTT
siCDK12_2	Reverse – AAAGAGAAUUCUUCUCGUAGACGCCUU
siCDK12_3	Forward – AGGAUUGGAAUACUGUCACAAAAAG
siCDK12_3	Reverse – CUUUUUGUGACAGUAUUCCAAUCCUUC

### Cell lines and culture conditions

Jurkat, 2D10, J-Lat 9.2, and 6.3 cells and GFP(-)-sorted HIV-1 latent infected cells were grown in RPMI 1640 (GIBCO) supplemented with 10% fetal bovine serum, penicillin-streptomycin, and L-glutamine at 37 °C in a humidified 5% CO_2_ incubator.

### Antibodies

Antibodies that were used for western blotting or Cut and Run: Anti-rabbit (Jackson Immuno research, Lot# 168738); anti-actin (Sigma, A5441, Lot # 030M4788); Anti-mouse (Jackson Immuno-research #115-035-062); Cyclin T1 (Abcam, ab27963); Cyclin K (Cell Signaling #19472S); CDK12 (abcam-EPR29009-30 ab317746); Pol II-CTD (Cell Signaling #2629S); Pol II-CTD Ser2 (Cell Signaling #13499S); Pol II CTD Ser5 (Cell Signaling #13523S); CDK9 (Cell Signaling #2316S); H3K27ac (Cell Signaling #8173S). MEPCE (ab185991).

### Isolation of primary CD4 + T cells

Human CD4 + T lymphocytes were isolated from six different healthy donors (both male and female donors were tested). PBMCs were isolated over a Ficoll gradient (Millipore) and were maintained at 5 × 10^5^ cells/mL at 37 °C. CD4 + T cells were isolated on the same day by negative selection using the RosetteSep Human CD4 + T-Cell Enrichment Cocktail (Stem Cell Technologies), resulting in homogeneous populations of CD4 + T cells (Day 0). CD4 + T cells were cultured in complete RPMI medium containing recombinant human IL-2 at 25 U/mL (Roche) to a final concentration of 5 × 10^5^ cells/ml and then stimulated using anti-CD3/CD28 Dynabeads (Invitrogen). Stimulated cells were counted, centrifuged for 5 min at 1200 rpm, and resuspended in fresh RPMI and IL-2 at a final concentration of 5 × 10^5^ cell/mL before transduction with high-titer HIV-1_GKO_ lentivirus (Day 2). Transduced cells were cultured for an additional 72 h in complete RPMI medium containing recombinant human IL2 and dynabeads at a ratio of 25 µL human beads per 10 million cells, before treated with SR-4835 (Day 5). 24 h post treatment, FACS analysis assessed HIV-1--1GFP levels in non-treated or treated cells (Day 6).

For monitoring effects of targeting CDK12/CDK13-CCNK by SR-4835 in primary CD4 infected T cells under cART, isolated cells were stimulated and then transduced with HIV-1 NLENG, a replication-competent virus that expresses GFP (a generous gift from David Levy; NYU). At Day 3, cART - Raltegravir (300 nM), Efavirenz (300 nM), Lenacapavir (100 nM), was applied on transduced cells for 7 days, during which suppression of HIV-1 replication was monitored by FACS. At day 10 post-isolation, cells were then treated with SR-4835 (1 µM) for 2 h, washed and kept growing for an additional 24 h. HIV-1 reactivation by SR-4835 was assessed two days later by FACS.

### Analysis of HIV-1 latency reversal in samples derived from PLWH under therapy

Study participants’ information on the PLWH whose PBMC were analyzed is available in Table EV1. 2.5 × 10^4^ PBMCs per well were seeded in 96-well plates in complete RPMI medium. The following day, cells were treated with SR-4835 (1 µM) or DMSO control for 2 h, washed, and harvested after culture for another 72 h. Analysis of cell viability by flow cytometry (Zombie Dye exclusion) revealed no significant impact of the short-term treatment with SR-4835 on cell viability. RNA was isolated using the RNAeasy kit (Qiagen) followed by reverse transcription into cDNA (Applied Biosystems) and qPCR analysis using the p1 and p10 primers for the detection of total, unspliced HIV-1 transcripts. Results were normalized to GAPDH mRNA levels.

### Ethics statement

Blood and leukapheresis samples were obtained under research protocols that were approved by the local Institutional Review Boards and ethics committees (University of Cologne 21-1543). All participants gave written informed consent, according to CARE (case report) guidelines and in compliance with the Declaration of Helsinki principles. In addition to the principles set out in the WMA Declaration of Helsinki, the experiments involving human subjects also conformed the Department of Health and Human Services Belmont Report.

### Purification of monocyte-derived macrophages

Monocytes were isolated from human PBMCs, using the EasySep enrichment kit. Cells were differentiated by treatment for 5 days with GM-CSF (Biotec Miltenyl) and stained with anti-CD14 APC-Cy7 and anti-CD20 conjugated antibody. By days 5–8 in culture, more than 95% of monocytes differentiated into macrophages, displaying characteristic spindle-shaped and multinucleated morphology and remaining positive for the CD14 marker.

### SR-4835 treatment

Cells were plated in 24-well flat-bottom plates at a density of 1 × 10^5^ cells/ml supplemented RPMI 1640 media. Cells were treated with SR-4835 (at different concentrations) for only 2 h or with TNFα for 6 h. Cells were then washed with PBS and incubated for an additional 48 h. Analysis was performed by flow cytometry, measuring HIV-1 GFP (+) cells. For synergistic effects of LRS and SR-4835, cells were treated with 0.5 μM SR-4835 for 2 h, followed by washing and treatment with 1 μM LRAs (Prostratin, JQ1, SAHA).

### Bliss independence model for synergism

Bliss synergy analysis was performed to assess combinatorial effects of two drugs. According to the Bliss independence model, the expected combined effect (F_AB_) is calculated as F_AB_ = F_A_ + F_B_ – (F_A_ × F_B_), F_A_ is the fractional effect of drug A alone, F_B_ is the fractional effect of drug B alone, F_AB_ is the expected additive effect if the drugs act independently. The Bliss score is then calculated as Biss score Δf_axy_ = Observed effect − F_AB_, where Bliss scores indicate synergistic effect Δfaxy > 0; an additive effect Δfaxy = 0; and a antagonistic effects Δfaxy < 0.

### Cell viability assays

Cell viability was measured using the XTT cell proliferation assay kit (Biological Industries, Israel) according to the manufacturer’s instructions. Briefly, 1 × 10^5^ cells were seeded per well in a 24-well plate, treated with SR-4835, and incubated for 48 h. XTT reagent was added, and absorbance was measured at 450 nm after 3 to 4 h using a microplate reader. Viability was calculated relative to untreated controls.

### Cell apoptosis assays

Cell apoptosis was analyzed using Annexin V-Pacific Blue and Propidium Iodide (PI) staining. Cells were harvested, centrifuged at 1500 rpm for 5 min, washed once with cold Annexin V binding buffer, and resuspended in 100 µL of binding buffer. Annexin V-Pacific Blue IgG (5 µL) was added, and samples were incubated for 15 min at room temperature in the dark. Prior to acquisition, 100 µL of binding buffer and PI (2.5 µL) were added. Samples were analyzed immediately by flow cytometry.

### Protein immunoprecipitation

2D10 (5 × 10^6^ cells) will be treated with 0.5 µM SR-4835 for 2 h, followed by washing with PBS. After an additional 6 h, the cells were harvested and lysed in a lysis buffer supplemented with RNase and protease inhibitors. 10% of the total lysate was used as input control. The lysate was then incubated on ice for 10 min, after which antibodies against CDK9, HEXIM1, or a non-specific IgG control were added. Samples were incubated overnight at 4 °C with rotation. The following day, magnetic dynabeads (Protein A or G) were added to the samples and incubated for 2 h at 4 °C. Samples were then treated with a magnet to collect the IP complexes, and the beads were washed three times with a washing buffer. The immunoprecipitated complexes were resuspended in a 5x reducing sample buffer and boiled for 5 min to denature the proteins. Finally, Western Blot analysis was performed to assess the protein interactions and determine the effects of CDK12/CDK13 inhibition on the association of P-TEFb complex with its partners.

### RNA isolation and sequencing

Total RNA was isolated from the infected cells using Fenozol reagent according to the manufacturer’s instructions. Infected cells were then harvested and resuspended in Fenozol reagent. The samples were mixed thoroughly to ensure complete lysis of the cells. After lysis, chloroform was added to the samples and vortexed for 15 s. The samples were then incubated at room temperature for 2–3 min before centrifugation at 12,000 × *g* for 15 min at 4 °C. This step facilitated phase separation. The upper aqueous phase containing the RNA was carefully transferred to a new tube, and an equal volume of isopropanol was added to the aqueous phase. The mixture was incubated at −20 °C for at least 30 min to precipitate the RNA. The samples were then centrifuged at 12,000 × *g* for 10 min at 4 °C to pellet the RNA. The supernatant was discarded, and the RNA pellet washed with 70% ethanol. The RNA pellet was then briefly air-dried and resuspended in RNase-free water or a suitable buffer. The quality and quantity of isolated RNA was assessed using a spectrophotometer or an RNA quantification assay. cDNA synthesis was conducted and followed by RNA-Seq libraries construction using a library preparation kit.

### Chromatin immunoprecipitation (ChIP-qPCR)

Treated and untreated cells were cross-linked with 1% formaldehyde for 25 min, and then cross-linking was stopped with glycine (137.5 mM; 5 min). Cells were washed twice with PBS, then lysed for 10 min on ice in 130 μL sonication buffer (20 mM Tris [pH 7.8], 2 mM EDTA, 0.5% SDS, protease inhibitor cocktail (Roche). DNA was fragmented by sonication at the following settings: amplitude 40% for 30 cycles at 10 s on/10 s off, and samples were centrifuged (15 min, 14,000 rpm, 4 °C). The soluble chromatin fraction was collected and immunoprecipitated overnight at 4 °C on a rotating wheel in IP buffer (0.5% Triton X-100, 2 mM EDTA, 20 mM Tris [pH 7.8], 150 mM NaCl, and 10% glycerol) with one of the indicated antibodies according to manufacturer’s instructions. Anti-Rabbit IgG was used as a background control. The next day, the IP material was incubated with 20 μL of protein G beads (Thermo 10003D) for 2 h. DNA was eluted with freshly prepared elution solution (1% SDS and 0.1 M NaHCO3) and heated at 65 °C overnight to reverse-crosslink the samples. Precipitated DNA fragments were then extracted using a ChIP DNA clean and concentrator kit (ZYMO Research), and HIV-1 DNA levels were quantified by qPCR with the primers specifically located on the LTR region of the HIV-1 promoter. All signals were calculated as percent of input DNA.

### Cleavage under targets & release using nuclease - CUT and RUN

CUT&RUN was performed using a commercial kit from Epicypher. SR-4835 treated 2D10 cells were analyzed in triplicate. After 24 h, cells were washed and coupled to Concanavalin-A-coated beads, followed by overnight incubation in Digitonin (0.01%)-containing buffer with antibodies targeting non-specific IgG, H3K4me3, RNA Pol II (CTD, Ser2, Ser5) or CDK9. After incubation, cells were washed with a permeabilization buffer and incubated with pAG-MNase for 15 min at room temperature. Additional washes were performed, and CaCl₂ was added to initiate chromatin cleavage at 4 °C for 2 h. The released DNA was eluted, spiked with *E. coli* DNA, and used to construct barcoded libraries with the Epicypher library preparation kit. Finally, DNA was quantified by Qubit. Libraries were sequenced and subjected to bioinformatic analysis. Sequences were aligned to the combined human (Hg38), *E. coli*, and HIV-1 reference genomes and normalized with IgG data. Enriched peaks for H3K4me3, RNA pol II (CTD, ser2 and ser5) or CDK9 were identified using SEARCS. For visualization, bigwig files were scaled and normalized using the *E. coli* spike-in DNA and visualized by Integrative Genomics Viewer (IGV).

### Data analysis

The analysis was carried out using the NeatSeq-Flow platform. Raw sequencing data in FASTQ format were trimmed by Trim Galore (version 0.4.5) (length = 25, q- 25), and reads that were too short were discarded. FASTQC, version 0.12.0, was used for read quality control. BWA Mapper (version 0.7.12, default parameters t = 20, B = 5) was used to align paired-end reads to the reference human genome assembly hg38 and to the spike-in control (*Escherichia coli*) reference genome assembly. SAMtools was used to remove PCR duplicates and create coordinate-sorted BAM files. Normalization between samples and IgG was performed with a custom script that calculates read scaling factor based on spike-in DNA reads of each sample. The scaling factor was then used in the bamCoverage to normalize each sample according to its spike-in. Bigwig files were created by bamCoverage function from deepTools (version 1.5.91), and reads mapped to blacklisted areas designated by ENCODE were filtered. Reads that refer to off-chromosome locations were removed with BedClip (version 377) and sorted by Bedtools (version 2.30.0). Bigwig files were then created with bedGraphToBigWig by deepTools. Peaks were called by SEACR (version 1.3), the top 1% of enriched regions in target data were selected, and peaks 100 bp or less apart were merged. Peaks were viewed in Integrated Genomics Viewer (version 2.16.1) (and annotated with Homer (version 4.11. Peaks 500 bp upstream or downstream from the TSS were selected for further analyses. Matrices were generated with DeepTools createMatrix, and Heatmaps were plotted with DeepTools plotHeatmap. The high-throughput sequencing data generated in this study have been deposited in the NCBI Gene Expression Omnibus (GEO) database.

### RNA immunoprecipitation

10^7^ Jurkat cells were washed with PBS and resuspended in 800 μl of RNA-IP buffer (0.5% NP-40, 20 mM HEPES pH 7.8, 100 mM KCl, 0.2 mM EDTA supplemented with *RNAseA* inhibitor (NEB) and protease inhibitor (Sigma). Lysates were incubated 10 min on ice and centrifuged 5000 rpm for 5 min at 4 °C. 50 μl of cell lysate was collected for input and stored at −80 °C. Supernatant was collected and incubated overnight with CDK9 IgG (Abcam ab6544) in cold room on a rocker. Next day 20 μl of pre-blocked BSA protein A beads were added and incubated for an additional 2 h in cold room on a rocker. Beads were washed x4 times with washing buffer (0.1% NP-40, 20 mM HEPES pH 7.8, 100 mM KCl, 0.2 mM EDTA supplemented with *RNase A* inhibitor (NEB) and protease inhibitor (Sigma). The pellet was resuspended in 100 μl of lysis buffer and extracted using TRIreagent (Sigma). RNA was reverse transcribed using AzuraQuant^TM^ II cDNA synthesis (Azura Genomics), and qPCR was performed using the indicated primers for 7SK snRNA.

7SK-F: GAG GGC GAT CTG GCT GCG ACAT

7SK-R: ACA TGG AGC GGT GAG GGA GGAA

Input RNA was extracted and reverse transcribed the same way. Dilutions of input were used for the standard curve and calculations.

### Splicing effects of SR-4835

To analyze changes in unspliced (Gag), single spliced (Env), and multiple spliced (Tat) vRNA species, nested qPCR assays were performed to target the relevant viral RNA species, Gag, Tat, and Env. Briefly, primary CD4 T cells were treated with 0.5 μM SR-4835 for 2 h and washed before being harvested at 48 h. DMSO was used as a control. Total RNA was isolated, treated with DNase, and transcript levels were quantified by RT-qPCR and normalized to GAPDH and to TAR to assess relative changes in HIV-1 RNA. Data are represented as fold of change according to the following. Formula (2^-((GAG-GAPDH)-(TAR-GAPDH)_(treated)_-(GAG-GAPDH)-(TAR-GAPDH)_(untreated)_)).

List of primers:

TAR_qPCR_F: GTTAGACCAGATCTGAGCCT

TAR_qPCR_R: GTGGGTTCCCTAGTTAGCCA

Gag_qPCR_F: ACATCAAGCAGCCATGCAAAT

Gag_qPCR_R: TCTGGCCTGGTGCAATAGG

Tat_qPCR_F: ATGGAGCCAGTAGATCCTAGACTAGAG

Tat_qPCR_R: CGTCGCTGTCTCCGCTTCTTCCT

Env_qPCR_F: CACCATGCTCCTTGGGATATT

Env_qPCR_R: GAGTGGTGGTTGCTTCCTT

qRT_GAPDH_F: TCGACAGTCAGCCGCATC

qRT_GAPDH_R: ACGACCAAATCCGTTGACTC

p1: GATTGGCAGAACTACACACCAGGGC

p10: GCGGCTGTCAAACCTCCACTC

Additional siRNA sequences are indicated in the reagent table.

### Statistical analysis

Data are presented as mean ± SD from three independent biological replicates. Statistical analysis was performed using one-way or two-way ANOVA in GraphPad Prism 7. For LRA synergism, one-way ANOVA was performed; ****p < 0.0001 and ***p < 0.001. For the siRNA experiments, a one-way ANOVA was conducted; **P < 0.01 and *P < 0.05. For cell viability and primary cell transduction, student t test was performed. **P < 0.01 and *P < 0.05. Statistical analysis: For 2D10 Reactivation, ChIP-qPCR and P-TEFb and DDR qPCR experiments were conducted by two-way ****P < 0.0001, ***P < 0.001; **P < 0.01. Experiments were processed in a randomized order to control for batch effects. Data analysts were blinded to condition labels during analysis, and all measurements were performed using at least three independent biological replicates, each conducted in technical triplicate.

## Supplementary information

Table EV1

Peer Review File

Expanded View Figures

## Data Availability

ChIP-seq, RNA-seq, and Cut and Run produced in this study are available in the following databases: ChIP Seq: NCBI Gene Expression Omnibus GSE316746 and RNA Seq: GSE316689. Cut and Run analysis: GSE316989. Source data are available in BioStudies, accession number: S-BSST3315 [Link for reviewers: https://www.ebi.ac.uk/biostudies/studies/S-BSST3315?key=13ca805b-be7a-4bb1-8562-e02f56b015c6]. The source data of this paper are collected in the following database record: biostudies:S-SCDT-10_1038-S44321-026-00536-z.
