# Supplementary material for: Inhibition of CDK12/CDK13-CYCLIN K rewires P-TEFb signaling to reverse HIV-1 latency and modulate cellular gene expression
Source: EMBO Mol Med. 2026 Oct 6;18(11):4558–84. doi: 10.1038/s44321-026-00536-z (PMC13748670; doi:10.1038/s44321-026-00536-z)
Supplement: Supplementary file 1 [file 44321_2026_536_MOESM1_ESM.pdf]

Patient-ID	sex	Age	HIV Diagnosis	ART (Y/N)	Regimen	ART naive (Y/N)	ART start	Viralload (cps/ml)	CD4 /µl	CD4 %	Ethnicity	Country/ Region of birth	HIV Risk
HAR0017	M	54	15/02/2009	Y	Padviram	N	15/06/2009	<20	960	43.7	Caucasian	Germany	MSM
HAR0019	W	43	15/06/2008	Y	Delstrigo	N	15/09/2011	<20	1100	43.8	NA	NA	Heterosexual
HAR0027	M	46	15/06/2021	Y	Dovato	N	29/09/2021	<20	750	28.7	Caucasian	Germany	Heterosexual
HAR0028	M	39	15/06/2009	Y	Dovato	N	15/06/2009	<20	940	47.5	Caucasian	Germany	MSM
HAR0029	M	44	19/10/2022	Y	Biktarvy	N	21/11/2023	<20	390	33.2	Caucasian	Europe	MSM
HAR0072	W	52	15/06/1999	Y	Padviram	N	15/06/2009	<20	540	41.1	Black	Africa	Heterosexual
HAR0092	W	52	30/01/2020	Y	Rekambys	N	15/02/2020	<20	720	24.3	Caucasian	Germany	Heterosexual
HAR0103	W	54	03/09/2003	Y	Biktarvy	N	28/10/2004	<20	510	53.7	Caucasian	Germany	Heterosexual
HAR0122	W	25	15/01/2021	Y	Emtricitabin/	N	08/01/2021	<20	1330	52.5	Caucasian	Germany	Heterosexual

Table EV1 - Study Participants of PLWH samples
