# Supplementary material for: Inhibition of CDK12/CDK13-CYCLIN K rewires P-TEFb signaling to reverse HIV-1 latency and modulate cellular gene expression
Source: EMBO Mol Med. 2026 Oct 6;18(11):4558–84. doi: 10.1038/s44321-026-00536-z (PMC13748670; doi:10.1038/s44321-026-00536-z)
Supplement: Supplementary file 2 [file 44321_2026_536_MOESM2_ESM.pdf]

EMBO Molecular Medicine

Inhibition of CDK12/CDK13-CYCLIN K rewires P-TEFb signaling to reverse HIV-1 latency and modulate cellular gene expression

Praveenkumar Murugavelu, Jagrity Rani, Heli Glebko, Ina Ambiel, Nadine Tibroni, Nili Tickotsky, Ferdinand Heyn, Philipp Schommers, Liron Levin, Alona Kuzmina, Oliver Fackler, and Ran Taube

Corresponding author(s): Ran Taube (rantaube@bgu.ac.il)

Review Timeline:

Transfer Date:	7th Apr 26
Editorial Decision:	8th Apr 26
Revision Received:	16th Aug 26
Editorial Decision:	11th Sep 26
Revision Received:	14th Sep 26
Accepted:	22nd Sep 26

Editor: Zeljko Durdevic

Transaction Report: This manuscript was transferred to EMBO Molecular Medicine following peer review at Review Commons.

(Note: With the exception of the correction of typographical or spelling errors that could be a source of ambiguity, letters and reports are not edited. Depending on transfer agreements, referee reports obtained elsewhere may or may not be included in this compilation. Referee reports are anonymous unless the Referee chooses to sign their reports.)

Review #1

1. Evidence, reproducibility and clarity:

Evidence, reproducibility and clarity (Required)

The study Murugavelu et al. addresses an important problem in HIV research-the persistence of latent viral reservoirs despite effective antiretroviral therapy (ART). Targeting transcriptional regulators such as CDK12/Cyclin K and their interaction with P-TEFb and the 7SK snRNP complex is highly relevant because transcription elongation is a critical control point in HIV latency and reactivation. The proposed mechanism linking CDK12 inhibition to P-TEFb release and HIV transcription activation is potentially novel and could have implications for latency-reversal strategies such as the "shock-and-kill" approach.

Overall, the manuscript is interesting and provides valuable insights into transcriptional regulation in HIV latency. The experiments appear solid and the findings are potentially relevant for the development of new latency-reversal strategies.
However there are some points to clarify and to improve.
I am surprised that Jurkat cell lines do not appear to be affected by treatment with SR-4835, a small-molecule inhibitor of cyclin-dependent kinases CDK12 and CDK13 that regulate transcription elongation and DNA damage-response gene expression. Since this compound has shown biological activity in several cellular contexts. This compound is mainly used as a research compound in cancer biology and has shown potential therapeutic relevance in several tumor types. The authors could briefly clarify why Jurkat cells appear resistant or unaffected in their experiments.
The data obtained using J-Lat cells are very interesting. If feasible, it would further strengthen the study to test additional J-Lat clones, since latency models can differ between clones.

The authors may consider including line numbers in the manuscript to facilitate the identification of typos and minor corrections. For example, in the abstract the word "makers" should be corrected to "markers."

It would also be helpful to include a schematic model or cartoon summarizing the proposed mechanism, which would make the main findings easier to understand for readers.

Another question concerns the extent of reservoir activation. With SR-4835, are all latent

reservoirs activated or only a subset of proviruses? If possible, imaging-based approaches examining activation at the single-provirus level could further strengthen the conclusions.

In addition, the percentages shown in Figure 6 are difficult to read, and the figure could be improved for clarity. Finally, the data from primary cells are presented differently from those obtained with J-Lat cells, making direct comparison difficult. The authors may consider using the same analysis and data representation for both systems.

2. Significance:

Significance (Required)

Overall, these comments are intended to improve clarity and presentation and do not affect the main conclusions of the manuscript, which I found kind of strong.

3. How much time do you estimate the authors will need to complete the suggested revisions:

Estimated time to Complete Revisions (Required)

(Decision Recommendation)

Less than 1 month

4. *Review Commons* values the work of reviewers and encourages them to get credit for their work. Select 'Yes' below to register your reviewing activity at Web of Science Reviewer Recognition Service (formerly Publons); note that the content of your review will not be visible on Web of Science.

Web of Science Reviewer Recognition

Yes

Review #2

1. Evidence, reproducibility and clarity:

Evidence, reproducibility and clarity (Required)

The current manuscript by Murugavelu eta l., titled "Targeting CDK12/CYCLIN K induces HIV gene activation and latency reversal which is mediated by P-TEFb" describes use of SR-4835, an inhibitor of CDK12/CCNK that has selectivity when tested against a panel of 460

kinases. Here the authors argue that SR-4835 acts as a molecular glue that targets the interactions between the CDK12, CYCLIN K (CCNK) and CUL4-RBX1-DDB1 ubiquitin ligase complex, and facilitating targeted protein degradation of CCNK and overall depletion of CDK12. They show that SR-4835 induces viral gene activation and latency reversal along with global cellular gene activation, by targeting CDK12/CCNK which modifies the epigenetic landscape around transcription starting sites inducing the release of P-TEFb from 7SKsnRNP. SR-4835 was applied on two cell types including HIV infected 2D10 cells with no apparent toxicity or cell viability. They observed similar results in J-Lat 9.2 cells. There was also comparison to other LRAs including Prostratin, SAHA, or JQ1, and I-BET.

Using IP for CDK9, the authors demonstrate that in SR-4835 treated cells, there was a marked decrease in HEXIM1 that was associated with CDK9 and CYCLIN T1, compared to untreated control
Cells. The inhibition of CDK12/CCNK inhibition by SR-4835 reactivates HIV gene transcription in latently infected primary CD4+ cells. Higher enrichment was detected for H3K4me3 levels, indicating a transcriptionally an active environment at the viral promoter that follows CDK12/CCNK targeting. Additional analysis documented elevated levels at TSS and gene bodies of H3K4me3, H3K7ac histone activation marks, as well as Pol II, Ser2P and Ser5P, indicating enhanced transcriptional initiation and elongation activity.

2. Significance:

Significance (Required)

The current manuscript by Murugavelu eta l., titled "Targeting CDK12/CYCLIN K induces HIV gene activation and latency reversal which is mediated by P-TEFb" describes use of SR-4835, an inhibitor of CDK12/CCNK that has selectivity when tested against a panel of 460
kinases. Here the authors argue that SR-4835 acts as a molecular glue that targets the interactions between the CDK12, CYCLIN K (CCNK) and CUL4-RBX1-DDB1 ubiquitin ligase complex, and facilitating targeted protein degradation of CCNK and overall depletion of CDK12. They show that SR-4835 induces viral gene activation and latency reversal along with global cellular gene activation, by targeting CDK12/CCNK which modifies the epigenetic landscape around transcription starting sites inducing the release of P-TEFb from 7SKsnRNP. SR-4835 was applied on two cell types including HIV infected 2D10 cells with no apparent toxicity or cell viability. They observed similar results in J-Lat 9.2 cells. There was also comparison to other LRAs including Prostratin, SAHA, or JQ1, and I-BET.

Using IP for CDK9, the authors demonstrate that in SR-4835 treated cells, there was a marked decrease in HEXIM1 that was associated with CDK9 and CYCLIN T1, compared to

untreated control
Cells. The inhibition of CDK12/CCNK inhibition by SR-4835 reactivates HIV gene transcription in latently infected primary CD4+ cells. Higher enrichment was detected for H3K4me3 levels, indicating a transcriptionally an active environment at the viral promoter that follows CDK12/CCNK targeting. Additional analysis documented elevated levels at TSS and gene bodies of H3K4me3, H3K7ac histone activation marks, as well as Pol II, Ser2P and Ser5P, indicating enhanced transcriptional initiation and elongation activity. Overall, I found the manuscript convincing, however there are some concerns including:

1. I am not sure that the panel of 460 kinases where initially purified from HIV-1 infected cells. Kinase from infected cells behave rather differently compared to uninfected cells in in vitro screening assays.
2. Have the authors tried side by side comparison of T-cell infection vs. macrophage infected cells for reactivation of the virus?
3. The Tox studies in cancer T-cells vs. primary T-cells in not convincing, since the half-life of the drugs is different in these cells. The primary cell data needs at least 6 male and 6 female PBMC infections with appropriate statistics to be convincing!
4. The primary cell PBMC experiments (+/- SR-4835) needs to be in the context of latent infected cells after 1-2 weeks of cART. Tox studies need to be shown here.
5. Many of the LRAs cause not only increased gene expression, but also changes in splicing favoring potential dynamic alterations in HIV doubly or singly spliced messages. Does SR-4835 also favor specific set of viral mRNAs in infected primary cells?

3. How much time do you estimate the authors will need to complete the suggested revisions:

Estimated time to Complete Revisions (Required)

(Decision Recommendation)

Between 3 and 6 months

4. *Review Commons* values the work of reviewers and encourages them to get credit for their work. Select 'Yes' below to register your reviewing activity at [Web of Science Reviewer Recognition Service] (formerly Publons); note that the content of your review will not be visible on Web of Science.

Web of Science Reviewer Recognition

No

Review #3

1. Evidence, reproducibility and clarity:

Evidence, reproducibility and clarity (Required)

This manuscript presents an interesting and potentially important finding: a compensatory interaction between CDK12 and CDK9 that can be exploited for HIV latency reversal using the small-molecule CDK12 inhibitor SR-4835. The RNA-seq data (Fig. 8) convincingly demonstrate that CDK9 compensates for CDK12 inhibition at both viral and cellular genes. However, several of the ChIP, infection, drug treatment, and synergy experiments require additional controls and statistical rigor to fully support the authors' conclusions.

Major Comments

1. Figure 2: The claim of synergy between SR-4835 and other LRAs requires formal testing. Bliss independence, Loewe additivity, or a comparable synergy model should be applied to substantiate this conclusion.
2. Figure 3: The data do not conclusively demonstrate that SR-4835-mediated latency reversal is CDK9-dependent. Flavopiridol is not specific to CDK9 and also inhibits CDK1, CDK2, CDK4, and CDK6. A more selective CDK9 inhibitor (e.g., AZD4573) or genetic depletion of CDK9 would provide stronger evidence that CDK9 activity is required for the observed effect.
3. Figure 4B: Panel 4B should include CCNT1 protein levels, similar to panel A. In addition, other 7SK snRNP components should be examined to more comprehensively support the conclusion that SR-4835 disrupts the inhibitory complex.
4. Figure 5C: Statistical comparisons between control and siCDK12 conditions are needed to support the conclusion that CDK12 depletion mimics SR-4835 treatment. Importantly, SR-4835 also inhibits CDK13 at the concentrations used. A CDK13 knockdown control should therefore be included to clarify whether the phenotype is specific to CDK12.
5. Figure 6B: The reported statistical significance between the control-latent and control-active groups does not make sense. The difference does not appear substantial in the graph. If the significance is valid, it raises concern that the control condition produces an effect comparable to SR-4835, which would contradict the stated conclusion. In addition, mean fluorescence intensity (MFI) values should be provided to assess transcriptional activity on a per-cell basis.

6. Figure 7A: Statistical testing should be applied to all groups, not only H3K4me3. Without this, conclusions regarding CDK9, Pol II, and Ser2/Ser5 phosphorylation are not sufficiently supported. Additionally, the IgG control appears to show increased signal following SR-4835 treatment. Is this increase statistically significant? If so, how is it explained? This raises concern about potential technical artifacts that should be addressed.

Minor Comments

1. In the section titled "Gene activation through targeting CDK12/CCNK is mediated by active CDK9", the first line contains a typographical error: "P-TEFb/CD9" should be corrected.
2. In Figure 2B-C, the y-axis formatting artificially breaks the bars and exaggerates visual differences. This should be revised for accurate data presentation.
3. Figure 5C is not called out in the manuscript.

2. Significance:

Significance (Required)

Overall, the study addresses an important mechanistic question and proposes a novel CDK12-CDK9 compensatory axis with therapeutic implications. With additional controls and more rigorous statistical analysis, the conclusions would significantly contribute to the HIV cure research.

3. How much time do you estimate the authors will need to complete the suggested revisions:

Estimated time to Complete Revisions (Required)

(Decision Recommendation)

Between 1 and 3 months

4. *Review Commons* values the work of reviewers and encourages them to get credit for their work. Select 'Yes' below to register your reviewing activity at Web of Science Reviewer Recognition Service (formerly Publons); note that the content of your review will not be visible on Web of Science.

Web of Science Reviewer Recognition

No

Revision Plan

Manuscript number: RC-2026-03432
Corresponding author(s): Ran Taube

[The "revision plan" should delineate the revisions that authors intend to carry out in response to the points raised by the referees. It also provides the authors with the opportunity to explain their view of the paper and of the referee reports.

The document is important for the editors of affiliate journals when they make a first decision on the transferred manuscript. It will also be useful to readers of the reprint and help them to obtain a balanced view of the paper.

If you wish to submit a full revision, please use our "Full Revision" template. ***It is important to use the appropriate template to clearly inform the editors of your intentions.****]*

1. General Statements [optional]

This section is optional. Insert here any general statements you wish to make about the goal of the study or about the reviews.

2. Description of the planned revisions

Insert here a point-by-point reply that explains what revisions, additional experimentations and analyses are planned to address the points raised by the referees.

3. Description of the revisions that have already been incorporated in the transferred manuscript

Please insert a point-by-point reply describing the revisions that were already carried out and included in the transferred manuscript. If no revisions have been carried out yet, please leave this section empty.

4. Description of analyses that authors prefer not to carry out

Please include a point-by-point response explaining why some of the requested data or additional analyses might not be necessary or cannot be provided within the scope of a revision. This can be due to time or resource limitations or in case of disagreement about the necessity of such additional data given the scope of the study. Please leave empty if not applicable.

Revision Plan

To: Editors of Review Commons and following journals
Re: **Full revision** - **Manuscript number: RC-2026-03432**

We would like to first thank the editor and three reviewers for taking the time to read our manuscript and provide productive comments and concerns: **"Targeting CDK12/CDK13-CYCLIN K induces HIV gene activation and latency reversal which is mediated by P-TEFb"**. We have carefully considered and fully addressed all comments and concerns that were raised by the reviewers, and then composed a full revision document. Some changes and clarifications were already incorporated into the revised manuscript; and in parallel, additional experiments are being performed to finalize the revision step. We are j that with the reviewers' comments and planned experiments, our manuscript will be substantially improved and hopefully suitable for publication.

Below is our full revision pan with a point-by-point response to the referees comments

Reviewer #1

1. I am surprised that Jurkat cell lines do not appear to be affected by treatment with SR-4835, a small-molecule inhibitor of cyclin-dependent kinases CDK12 and CDK13 that regulate transcription elongation and DNA damage-response gene expression. Since this compound has shown biological activity in several cellular contexts. …..

We thank the reviewer for this important comment and would like to clarify that although SR-4835 has demonstrated biological activity of activating HIV gene expression and reverse latency in multiple cellular systems, our experiments did observe some toxicity effects in Jurkat cells, reaching to about 20% cell death across all experiments (Fig. 1A). Our results also indicate that SR-4835 led to minimal apoptosis levels 16% (see revised Fig S1). Moreover, the experimental scheme of treating cells with SR-4835 includes a short incubation time of the drug with the cells, 2hr, followed by washing the cells thus minimizing toxic effects. Importantly, as shown in the RNA Seq analysis, treatment of Jurkat cells with the SR-4835 results in substantial changes in gene expression of downstream targets of CDK12/CDK13 (Fig 8).

2. The authors could briefly clarify why Jurkat cells appear resistant or unaffected in their experiments.

We thank the reviewer for this comment. As mentioned above, Jurkat cells were not truly resistant to drug inhibitor treatment; SR-4835 treatment consistently induced some cytotoxicity, of about ~20-25% cell death across all experiments as well as minimal apoptosis. Moreover, the experimental scheme of treating cells with SR-4835 includes a short incubation time of the drug and cells, 2hr, followed by washing the cells Additionally, we observed reduced protein stability of CDK12 and CCNK, supporting the activity of the SR-4835 inhibitor in these cells (Fig 1A&B).

3. The data obtained using J-Lat cells are very interesting. If feasible, it would further strengthen the study to test additional J-Lat clones, since latency models can differ between clones.

We thank the reviewer for this valuable suggestion. We agree that different J-Lat clones can exhibit distinct latency characteristics due to differences in the proviral integration site and, as a result, perturbations in HIV transcriptional regulation. To address this point, we plan to extend our analysis to additional J-Lat clones and evaluate whether the observed effects of SR-4835 are consistent across multiple latency models.

4. The authors may consider including line numbers in the manuscript to facilitate the identification of typos and minor corrections. For example, in the abstract the word “makers” should be corrected to “markers”.

We carefully reviewed the manuscript and corrected all typos; line numbers were also included to facilitate easier reference.

5. Another question concerns the extent of reservoir activation. With SR-4835, are all latent reservoirs activated or only a subset of proviruses? If possible, imaging-based approaches examining activation at the single- provirus level could further strengthen the conclusions.

We thank the reviewer for this insightful comment. A single-cell imaging-based analysis may provide additional insights into the heterogeneity of proviral activation by SR-4835. We think that such analysis is beyond the scope of the current study. Nevertheless, for future work, we plan to initiate single-cell analysis to monitor the extent and cell heterogenicity of the reservoir following drug treatment.

6. The percentages shown in Figure 6 are difficult to read, and the figure could be improved for clarity.

As requested, we addressed this comment and redesigned the figure that describes the effects of SR-4538 on primary CD4 T cells. We have already adjusted the font size and color contrast of the percentage labels. Hopefully, this will make the data clearer and more visually accessible, allowing readers to interpret the results.

7. Finally, the data from primary cells are presented differently from those obtained with J-Lat cells, making direct comparison difficult. The authors may consider using the same analysis and data representation for both systems.

We thank the reviewer for this helpful suggestion. Indeed, the presentation of data from primary cells and J-Lat models makes direct comparison difficult. To address this, we have revised Figure 6 employing the same analysis and graphical format as shown for the J-Lat cells. Nevertheless, we think that the FACS blots are required as they show the actual raw data of this experiment.

Reviewer #2

1. I am not sure that the panel of 460 kinases where initially purified from HIV-1 infected cells. Kinase from infected cells behave rather differently compared to uninfected cells in in vitro screening assays.

We thank the reviewer for this comment. SR-4835 is indeed a highly selective inhibitor of CDK12 and CDK13 and functions via degrading CYCLIN K and not affecting the specific kinase activity of either CDK12 or CDK13. Previous kinase profiling studies have demonstrated that SR-4835 exhibits strong selectivity for these both kinases over a broad kinase panel (>450 kinases), with minimal off-target activity and low nanomolar IC_{50} and Kd values (Quereda etal 2019; Schmitz etal 2023). This point has been incorporated into the manuscript's intriduction to clarify this point.

2. The authors should try side by side comparison of T-cell infection vs. macrophage infected cells for reactivation of the virus?

We thank the reviewer for this interesting suggestion. In our work, all mechanistic experiments and reactivation assays were performed in CD4 positive T-cell-based latency models, which were chosen to reflect the major, and well-characterized latent reservoir of infected $CD4^+$ T cells. Although highly interesting, we did not establish a parallel latency model in primary macrophages or macrophage-like cells for this

manuscript. This is beyond the scope of this work and indeed a possible future direction to explore.

3. The Tox studies in cancer T-cells vs. primary T-cells are not convincing, since the half-life of the drugs is different in these cells. The primary cell data needs at least 6 male and 6 female PBMC infections with appropriate statistics to be convincing!

We have performed multiple experiment in HIV infected naïve primary CD4 T cells from both male and female donors. Statistical significances were incorporated into the revised manuscript -see revised Fig. 6. We also elaborated this point in the methods section in the revised manuscript. We are certain that this will convince the reviewer of the true relevance of the effects of SR4835 in primary cells.

4. The primary cell PBMC experiments (+/- SR-4835) needs to be in the context of latent infected cells after 1-2 weeks of cART. Tox studies need to be shown here.

We agree with this reviewer for this valid comment. Indeed, evaluating the potential activation effects of SR-4835 in the context of a clinical settings of HIV infected individuals is of high significant. However, for this to be implemented, we will need to issue a specific Helsinki authorization, which will take some time, especially with the current political environment in Israel. We nevertheless would like to peruse this direction in the form of suitable collaborations, but cannot guarantee fulfillment of this point.

5. Many of the LRAs cause not only increased gene expression, but also changes in splicing favoring potential dynamic alterations in HIV doubly or singly spliced messages. Does SR-4835 also favor specific set of viral mRNAs in infected primary cells?

We thank the reviewer for this insightful question. In our system, GFP expression serves as a readout of productive HIV transcription and reflects the presence of spliced viral mRNAs. While this indicates that SR-4835 activates HIV transcription, we did not specifically distinguish between singly and doubly spliced viral transcripts; however, we agree this is an important point and should be investigated. We think that at this step of the research such a point is beyond the scope of the current work, which introduces the

mechanistic explanation for the activation effects of SR-4835 on HIV gene expression and viral latency.

Reviewer #3

1. Figure 2: The claim of synergy between SR-4835 and other LRAs requires formal testing. Bliss independence, Loewe additivity, or a comparable synergy model should be applied to substantiate this conclusion.

We thank the reviewer for this important suggestion. We agree that formal synergy analysis is required to substantiate the claim of combinatorial effects between SR-4835 and other LRAs. In the revised manuscript, we already performed synergy assessment analysis using the Bliss independence model that helps to compare observed versus expected responses to derive synergy scores. (see Fig 2).

2. Figure 3: The data do not conclusively demonstrate that SR-4835-mediated latency reversal is CDK9-dependent. Flavopiridol is not specific to CDK9 and also inhibits CDK1, CDK2, CDK4, and CDK6. A more selective CDK9 inhibitor (e.g., AZD4573) or genetic depletion of CDK9 would provide stronger evidence that CDK9 activity is required for the observed effect.

We thank the reviewer for this important point. Flavopiridol is widely used as a functional inhibitor of CDK9 kinase activity, inhibiting pause-release of Pol II and transcriptional elongation, particularly in the context of HIV transcription, which is heavily dependent on CDK9/P-TEFb. Flavopiridol is a sub-10nM inhibitor of CDK9 in most biochemical assays, while for other CDKs its IC_{50} values are higher, i.e weaker inhibitory functions. In the context of HIV gene expression regulation that is solely dependent on CCT1 and CDK9, Ki values are ≈ 3 nM, and reported IC_{50} are at the 2–20nM in biochemical kinase assays, therefore Flavopiridol is considered the primary high-affinity targeting CDK9. Nevertheless, we acknowledge that the SR-4835 inhibitor may also inhibit the kinase activity of additional CDKs like CDK1, CDK2, CDK4, CDK6. Reported IC_{50} values for these kinases vary in the literature depending in the assay performed. For CDK2 (IC_{50} -100nM), CDK4 and CDK6 (IC_{50} 20-100nM), and CDK7 100-

300nM (Carson etal. Cancer Research 1996: Chen etal. Blood 2025). These CDKs that may be targeted by Flavopiridol are primarily associated with cell cycle regulation rather than control of transcriptional elongation, making them less likely to account for the observed effect observed in our work.
To further strengthen the conclusion that SR-4835-mediated inhibition promotes P-TEFb-mediated gene activation, we plan to include additional experiments that will confirm our conclusions. As proposed by the reviewer, we will use a more selective CDK9 inhibitor, AZD4573, and in addition peruse attempts to genetically perturb CDK9 expression by siRNA.

3. Figure 4B: Panel 4B should include CCNT1 protein levels, similar to panel A. In addition, other 7SK snRNP components should be examined to more comprehensively support the conclusion that SR-4835 disrupts the inhibitory complex.

We thank the reviewer for this thoughtful suggestion, and agree that monitoring the expression of additional components of the 7SK snRNP will further strengthen our concept of SR-4835-mediated disruption of the P-TEFb-7SK snRNP complex. While our current data already indicate that SR-4835 disrupts the interactions between P-TEFb and 7SK snRNP inhibitory complex, we plan to strengthen this conclusion and include CCNT1 protein levels in the western blot analysis (Figure 4B), similar to panel 4A. Regarding the additional 7SK snRNP components, we plan to monitor 7SK snRNA levels by RNA-IP analysis followed by qPCR, as well as monitoring MeCPE (another component of the 7SK snRNP complex).These additional analyses will provide a more comprehensive demonstration of SR-4835–mediated disruption of the inhibitory complex in the revised manuscript.

4. Figure 5C: Statistical comparisons between control and siCDK12 conditions are needed to support the conclusion that CDK12 depletion mimics SR-4835 treatment. Importantly, SR-4835 also inhibits CDK13 at the concentrations used. A CDK13 knockdown control should therefore be included to clarify whether the phenotype is specific to CDK12.

We thank the reviewer for this justified comment. We would like to clarify that our study specifically targets CYCLIN K (CCNK), the regulatory cyclin subunit of either CDK12 or CDK13. SR-4835 acts by degrading CCNK, disrupting of the CDK12/CDK13-CCNK complex and depleting the kinases; Thus, the observed inhibitory effect of SR-4835 reflects CCNK degradation rather than direct inhibition of CDK12 or CDK13 kinase activity. We accordingly clarified this point in the revised manuscript. Moreover, previous reports nicely show that SR-4835 exhibits higher affinity to CDK12 than CDK13.

Importantly, depending on the applied assay, reported IC_{50} values are at a range of 60nM for CDK12/CCNK and 366nM for CDK13/CCNK, and much higher for other kinases. Other approach determined IC_{50} values of at the range of 84.3 to 156 nM for CDK12/CCNK and 366 to 400 nM for CDK13/CCNK. SR-4835 displays no inhibitory activity toward CDK7, CDK8, and CDK9, while a moderate inhibition for CDK10 which is x10 fold lower (Schmitz et al. JBC 2023). Up to date, development of compounds that can discriminate between closely related kinases CDK12 and CDK13 and exclusively target one of these kinases has not been reported. We thoroughly highlighted this point in the revised manuscript.

Nevertheless, as suggested by the reviewer, we plan to use siRNA to transiently (and for a short time) to knockdown both CDK12 and thus strengthening our conclusions regarding effects of SR-4835 on CDK12/CDK13. We hope that these experiments will be fruitful.

5. Figure 6B: The reported statistical significance between the control - latent and control-active groups does not make sense. The difference does not appear substantial in the graph. If the significance is valid, it raises concern that the control condition produces an effect comparable to SR-4835, which would contradict the stated conclusion.

In addition, mean fluorescence intensity (MFI) values should be provided to assess transcriptional activity on a per-cell basis.

We thank the reviewer for raising the important point of statistical significance in Figure 6B. We agree that the reported statistical significance between the control latent and control active groups is incorrect and was due to labelling error. We have clarified the experimental design and already revised text and figure accordingly. Importantly, this does not affect the overall conclusion, as SR-4835 treatment results in a clear and reproducible activation of HIV gene expression compared to the control conditions. We also appreciate the suggestion to include mean fluorescence intensity (MFI) values. We agree that this would provide additional insight into transcriptional activity at the single-cell level, and we already included this data in the revised figure.

6. Figure 7A: Statistical testing should be applied to all groups, not only H3K4me3. Without this, conclusions regarding CDK9, Pol II, and Ser2/Ser5 phosphorylation are not sufficiently supported. Additionally, the IgG control appears to show increased signal following SR-4835 treatment. Is this increase statistically significant? If so, how is it explained? This raises concern about potential technical artifacts that should be addressed.

We thank the reviewer for this important suggestion. We agree that statistical analysis should be applied consistently across all datasets. Accordingly, we have now performed statistical analysis for all groups shown in Figure 7A, including CDK9, RNA Polymerase II, and Ser2/Ser5 phosphorylation. The updated statistical analyses have already incorporated into the revised manuscript and the corresponding figure. These analyses further support the conclusions presented in the manuscript. The IgG control signal following SR-4835 treatment shows a minor increase; however, this difference is not statistically significant.

In summary, we again would like to acknowledge the editor and reviewers for reading our manuscript and raise comments. We hope that by addressing all of the reviewers concerns our much-improved manuscript will be suitable for publication

Sincerely
Ran Taube, PhD

References:

1. Victor Quereda, Simon Bayle, Francesca Vena, Sylvia M. Frydman, Andrii Monastyrskyi, William R. Roush, Derek R. Duckett, Therapeutic Targeting of CDK12/CDK13 in Triple-Negative Breast Cancer, Cancer Cell, https://doi.org/10.1016/j.ccell.2019.09.004.

2. Chohan, T. A., Qayyum, A., Rehman, K., Tariq, M., & Akash, M. S. H. (2018). An insight into the emerging role of cyclin-dependent kinase inhibitors as potential therapeutic agents for the treatment of advanced cancers. *Biomedicine & pharmacotherapy = Biomedecine & pharmacotherapie*, *107*, 1326–1341. https://doi.org/10.1016/j.biopha.2018.08.116

3. Bonvini P, Zorzi E, Mussolin L, Monaco G, Pigazzi M, Basso G, Rosolen A. The effect of the cyclin-dependent kinase inhibitor flavopiridol on anaplastic large cell lymphoma cells and relationship with NPM-ALK kinase expression and activity. Haematologica. 2009 Jul;94(7):944-55. doi: 10.3324/haematol.2008.004861. Epub 2009 Jun 16. PMID: 19535344; PMCID: PMC2704305.

8th Apr 2026

Dear Prof. Taube,

Thank you for the submission of your research manuscript to our editorial offices. I have now had the opportunity to read the manuscript, the referee reports and your point-by-point response and to discuss it with the other members of our editorial team. We all agreed that the manuscript fits the scope of EMBO Molecular Medicine but also that the referees raised important concerns that should be addressed in major revision. In our opinion your revision plan addresses the referees' criticism adequately and therefore we would like to invite submission of the revised manuscript to our journal.

Further consideration of a revision that addresses reviewers' concerns in full will entail a second round of review. EMBO Molecular Medicine encourages a single round of revision only and therefore, acceptance or rejection of the manuscript will depend on the completeness of your responses included in the next, final version of the manuscript. For this reason, and to save you from any frustrations in the end, I would strongly advise against returning an incomplete revision.

We would welcome the submission of a revised version within three months for further consideration. Please let us know if you require longer to complete the revision.

I look forward to receiving your revised manuscript.

Yours sincerely,

Zeljko Durdevic

Zeljko Durdevic
Senior Editor
EMBO Molecular Medicine

*****

When preparing your revised manuscript, please refer to our guidelines: https://link.springer.com/journal/44321/submission-guidelines#cms-Revised-submissions. We perform an initial quality control of all revised manuscripts before re-review; failure to include requested items will delay the evaluation of your revision.

We require:

1) A .docx formatted version of the manuscript text (including legends for main figures, EV figures and tables). Please make sure that the changes are highlighted to be clearly visible.

2) Individual production quality figure files as .eps, .tif, .jpg (one file per figure). For guidance, download the 'Figure Guide PDF': https://media.springernature.com/original/springer-cms/rest/v1/content/27825798/data/v1.

3) A .docx formatted letter INCLUDING the reviewers' reports and your detailed point-by-point responses to their comments. As part of the EMBO Press transparent editorial process, the point-by-point response is part of the Review Process File (RPF), which will be published alongside your paper.

4) A complete author checklist, which you can download from our author guidelines. Please insert information in the checklist that is also reflected in the manuscript. The completed author checklist will also be part of the RPF.

5) Please note that all corresponding authors are required to supply an ORCID ID for their name upon submission of a revised manuscript.

6) It is mandatory to include a 'Data Availability' section after the Materials and Methods. Before submitting your revision, primary datasets produced in this study need to be deposited in an appropriate public database, and the accession numbers and

database listed under 'Data Availability'. Please remember to provide a reviewer password if the datasets are not yet public.

In case you have no data that requires deposition in a public database, please state so in this section. Note that the Data Availability Section is restricted to new primary data that are part of this study.

7) For data quantification: please specify the name of the statistical test used to generate error bars and P values, the number (n) of independent experiments (specify technical or biological replicates) underlying each data point and the test used to calculate p-values in each figure legend. The figure legends should contain a basic description of n, P and the test applied. Graphs must include a description of the bars and the error bars (s.d., s.e.m.).

8) At EMBO Press we ask authors to provide source data for the main manuscript figures. You will receive a separate email with instructions for providing source data with your revised manuscript, including how to upload and organize the files.

9) Our journal encourages inclusion of *data citations in the reference list* to directly cite datasets that were re-used and obtained from public databases. Data citations in the article text are distinct from normal bibliographical citations and should directly link to the database records from which the data can be accessed. In the main text, data citations are formatted as follows: "Data ref: Smith et al, 2001" or "Data ref: NCBI Sequence Read Archive PRJNA342805, 2017". In the Reference list, data citations must be labeled with "[DATASET]". A data reference must provide the database name, accession number/identifiers and a resolvable link to the landing page from which the data can be accessed at the end of the reference.

10) We replaced Supplementary Information with Expanded View (EV) Figures and Tables that are collapsible/expandable online. A maximum of 5 EV Figures can be typeset. EV Figures should be cited as 'Figure EV1, Figure EV2" etc... in the text and their respective legends should be included in the main text after the legends of regular figures.

- For the figures that you do NOT wish to display as Expanded View figures, they should be bundled together with their legends in a single PDF file called *Appendix*, which should start with a short Table of Content. Appendix figures should be referred to in the main text as: "Appendix Figure S1, Appendix Figure S2" etc.

- Additional Tables/Datasets should be labeled and referred to as Table EV1, Dataset EV1, etc. Legends have to be provided in a separate tab in case of .xls files. Alternatively, the legend can be supplied as a separate text file (README) and zipped together with the Table/Dataset file.

11) The paper explained: EMBO Molecular Medicine articles are accompanied by a summary of the articles to emphasize the major findings in the paper and their medical implications for the non-specialist reader. Please provide a draft summary of your article highlighting

- the medical issue you are addressing,

- the results obtained and

- their clinical impact.

This may be edited to ensure that readers understand the significance and context of the research. Please refer to any of our published articles for an example.

12) Author contributions: You will be asked to provide CRediT (Contributor Role Taxonomy) terms in the submission system. These replace a narrative author contribution section in the manuscript.

13) A Conflict of Interest statement should be provided in the main text.

14) Every published paper includes a 'Synopsis' to further enhance discoverability. Synopses are displayed on the journal webpage and are freely accessible to all readers. They include a short stand first (maximum of 300 characters, including space) as well as 2-5 one-sentences bullet points that summarizes the paper. Please write the bullet points to summarize the key NEW findings. They should be designed to be complementary to the abstract - i.e. not repeat the same text. We encourage inclusion of key acronyms and quantitative information (maximum of 30 words / bullet point). Please use the passive voice. Please attach these in a separate file or send them by email, we will incorporate them accordingly.

Please also suggest a striking image or visual abstract to illustrate your article as a PNG file 550 px wide x 300-600 px high.

15) Include a Reagents and Tools Table as part of the Methods section, which can be downloaded from our author guidelines.

EMBO Molecular Medicine has a "scooping protection" policy, whereby similar findings that are published by others during review or revision are not a criterion for rejection. Should you decide to submit a revised version, I do ask that you get in touch after three months if you have not completed it, to update us on the status.

4/8/2026

Manuscript number: RC-2026-03432
Corresponding author(s): Ran Taube

To: EMBO – Molecular Medicine
Re: **Full revision** - **Manuscript number:** EMM-2026-23779-T

Dear editor

We hereby submit a full revision of our revised manuscript entitled **"Targeting CDK12/CDK13-CYCLIN K induces HIV-1-specific and global gene activation mediated by P-TEFb"**. We want to thank you and the reviewers for taking the time to read our manuscript and for providing their constructive comments. We have carefully considered and fully addressed all concerns raised by the reviewers, including all suggested experiments. Accordingly, we modified our manuscript and incorporated the changes and clarifications that were raised.

Below is a point-to-point response to all referees' comments:

Reviewer #1

1. I am surprised that Jurkat cell lines do not appear to be affected by treatment with SR-4835, a small-molecule inhibitor of cyclin-dependent kinases CDK12 and CDK13 that regulate transcription elongation and DNA damage-response gene expression. Since this compound has shown biological activity in several cellular contexts. …..

We thank the reviewer for this comment and would like to clarify that although SR-4835 has demonstrated biological activity of activating HIV-1 gene expression and reverse of latency in multiple cellular systems, our experiments did observe some toxicity effects of about 20% cell death across all cell models (**Fig. 1A**). Our results also indicate that SR-4835 led to minimal apoptosis (**Fig S1**). Moreover, the experimental scheme of treating cells with SR-4835 includes only a short incubation time of the drug with the cells, 2hr, followed by a washing step, therefore minimizing toxic effects of SR-4835. Importantly, as shown in the RNA-Seq analysis, treatment of Jurkat cells with the SR-4835 results in substantial changes in gene expression of downstream targets of CDK12/13 (**Fig 8**).

2. The authors could briefly clarify why Jurkat cells appear resistant or unaffected in their experiments.

We thank the reviewer for this comment. As mentioned above, Jurkat cells were not truly resistant to the inhibitor treatment; SR-4835 treatment consistently induced some levels of cytotoxicity,

about ~20% cell death across all experiments, as well as minimal apoptosis effects. Additionally, we observed reduced protein stability of CDK12 and CCNK, supporting the activity of the SR-4835 inhibitor in these cells (**Fig 1A&B**).

3. The data obtained using J-Lat cells are very interesting. If feasible, it would further strengthen the study to test additional J-Lat clones, since latency models can differ between clones.

We thank the reviewer for this valuable suggestion. We agree that different J-Lat clones may exhibit distinct latency characteristics due to differences in the proviral integration site and, therefore, perturbations in HIV-1 transcription regulation. To address this point, we extended our analysis to an additional J-Lat 6.3 clone that carries full-length HIV-1 where GFP is inserted instead of the Nef gene. We evaluated the effects of SR-4835 on this specific latency cell models (**Fig. 1**). Our new analysis conform that the activation effects of SR-4835 are consistent across multiple latency models.

4. The authors may consider including line numbers in the manuscript to facilitate the identification of typos and minor corrections. For example, in the abstract the word “makers” should be corrected to “markers”.

We carefully reviewed the manuscript and corrected all typos; line numbers were also included to facilitate easier reference.

5. Another question concerns the extent of reservoir activation. With SR-4835, are all latent reservoirs activated or only a subset of proviruses? If possible, imaging-based approaches examining activation at the single- provirus level could further strengthen the conclusions.

We thank the reviewer for this insightful comment. A single-cell imaging-based analysis should provide additional insights into the heterogeneity of proviral activation by SR-4835. We think that such analysis is beyond the scope of the current study. Nevertheless, for future work, we plan to initiate single-cell analysis to monitor the extent and cell heterogenicity of the reservoir following drug treatment.

6. The percentages shown in Figure 6 are difficult to read, and the figure could be improved for clarity.

As requested, we addressed this comment and redesigned the figure that describes the effects of SR-4835 on primary CD4 T cells. Hopefully, this will make the data clearer and more visually accessible, allowing readers to interpret the results. The previous data are included in the revised manuscript in the supplementary section (**Fig. S3**).

7. Finally, the data from primary cells are presented differently from those obtained with J-Lat cells, making direct comparison difficult. The authors may consider using the same analysis and data representation for both systems.

We thank the reviewer for this helpful suggestion. Indeed, the presentation of data from primary cells and J-Lat models makes direct comparison difficult. To address this, we have revised Figure 6 (**now Fig. S3**), employing the same analysis and graphical format as shown for the J-Lat cells. Nevertheless, we think that the FACS blots are required as they show the actual raw data of this experiment. Importantly, a revised Fig. 6 includes analysis for the effects of SR-4835 in human CD4 primary cells under cART.

Reviewer #2

1. I am not sure that the panel of 460 kinases where initially purified from HIV-1 infected cells. Kinase from infected cells behave rather differently compared to uninfected cells in in vitro screening assays.

We thank the reviewer for this comment. SR-4835 is indeed a highly selective inhibitor for CDK12 and CDK13, and functions via degrading CYCLIN K. As reported its does not directly affect the kinase activity of either CDK12 or CDK13. Additional kinase profiling studies show that SR-4835 exhibits strong selectivity for these both kinases over a broad kinase panel (>450 kinases), with minimal off-target activity. Moreover, it functions at relatively low nanomolar IC_{50} and Kd values (Quereda etal 2019; Schmitz etal 2023). This point has been incorporated into the manuscript's introduction to clarify this point.

2. The authors should try a side-by-side comparison of T-cell infection vs. macrophage-infected cells for reactivation of the virus?

We thank the reviewer for this interesting suggestion. In our work, all mechanistic experiments and reactivation assays were performed in CD4-positive T-cell-based latency models, which were chosen to reflect the major and well-characterized HIV-1 latent reservoir of infected $CD4^+$ T cells. As suggested by the reviewer, we also tested the effects of SR-4835 inhibition of CDK12/CDK13 on human primary monocyte-derived macrophages that were infected with HIV-1. Our results confirm a minimal effect of SR-4835 on macrophages; however, lower than the effects seen with primary CD4 T cells. In the discussion, we addressed the possible mechanisms for such differences and hypothesized that restrictions on HIV-1 reactivation that are specific to macrophages may play a role in the relatively low effects of SR-4835 (**Fig. S4**). We also tested effects of SR-4835 treatment on U1 monocytes, which are infected with HIV-1 and serve as a model for HIV latency, as they carry an integrated defective provirus with a point mutation in the viral Tat protein. In agreement with our results, U1-treated monocytes exhibited significant reactivation of latent HIV-1 (**Fig. S4**).

3. The Tox studies in cancer T-cells vs. primary T-cells are not convincing, since the half-life of the drugs is different in these cells. The primary cell data needs at least 6 male and 6 female PBMC infections with appropriate statistics to be convincing!

As suggested by the reviewer, we have performed multiple experiments in HIV-1-infected naïve primary CD4 T cells from both male and female donors, up to 6 donors. Statistical significances were incorporated into the revised manuscript – please refer to the revised **Fig. S3**. Additional experiments were performed in CD4 primary cells that were treated with cART, before the administration of SR-4835 (**Fig. 6**). Our results show that SR-4835 has the potential to reactivate latent HIV-1 in primary CD4 T cells that are under therapy. We are certain that this will convince the reviewer of the relevance of the effects of SR-4835 on primary CD4 T cells. As requested, we also monitored the toxicity of SR-4835 on primary CD4 T cells and confirmed relatively low toxicity as shown in **Fig. 6C** of the revised manuscript.

4. The primary cell PBMC experiments (+/- SR-4835) needs to be in the context of latent infected cells after 1-2 weeks of cART. Tox studies need to be shown here.

We agree with the reviewer on this valid comment. Indeed, evaluating the potential reactivation effects of SR-4835 in HIV-1 infected individuals under therapy is of high clinical significant. We therefore designed an experiment, where naïve CD4+ primary T cells from two different donors were purified from PBMCs, stimulated with anti-CD3/CD28 beads, and transduced with replication-competent HIV-1 GFP at Day 3. Cells were then treated with cART, and viral replication was monitored over a period of 7 days to confirm suppression of HIV-1 replication. At day 10 post-isolation, cells were treated with SR-4835 or with control DMSO for 2 hours and 24 hours later (Day 11) monitored by FACS for HIV-GFP viral replication. Our results confirm that indeed cART treatment suppressed HIV gene expression in primary CD4 T cells, and the latent viral gene expression was activated upon treating cells with SR-4835 (**Fig. 6A+B**). This was evident in the four donors examined and both the percentage of HIV-1 GFP and MFI levels (**Fig. 6D)**. Also presented are new toxicity experiments, confirming low toxicity on primary CD4 T cells, although higher relative to T cell lines (**Fig. 6C**). In addition, the potential of SR-4835 as a latency reversal agent of latent HIV-1 was also documented in cells derived from people living with HIV-1 under therapy. upon treatment with SR-4835, HIV-1 gene activation was observed in most donors (n=9) and as expected, to a different extent (**Fig 6D**).

5. Many of the LRAs cause not only increased gene expression, but also changes in splicing, favoring potential dynamic alterations in HIV doubly or singly spliced messages. Does SR-4835 also favor a specific set of viral mRNAs in infected primary cells?

We thank the reviewer for this insightful question. As suggested, we monitored the levels of viral RNA in primary CD4 T cells that are either not spliced, single spliced and multiple spliced. Our data show only minimal effects of SR-4835 treatment on viral RNA splicing, implying that main contribution of SR-4835 is at the transcription activation levels. (**Fig S5**).

Reviewer #3

1. Figure 2: The claim of synergy between SR-4835 and other LRAs requires formal testing. Bliss independence, Loewe additivity, or a comparable synergy model should be applied to substantiate this conclusion.

We thank the reviewer for this important suggestion. We agree that formal synergy analysis is required to substantiate the claim of combinatorial effects between SR-4835 and other LRAs. In the revised manuscript, we performed synergy assessment analysis using the Bliss independence model that helps to compare observed versus expected responses to derive synergy scores (**Fig 2D**).

2. Figure 3: The data do not conclusively demonstrate that SR-4835-mediated latency reversal is CDK9-dependent. Flavopiridol is not specific to CDK9 and also inhibits CDK1, CDK2, CDK4, and CDK6. A more selective CDK9 inhibitor (e.g., c) or genetic depletion of CDK9 would provide stronger evidence that CDK9 activity is required for the observed effect.

We thank the reviewer for this important point. Flavopiridol is commonly used as a functional inhibitor of the kinase activity of CDK9, inhibiting pause-release of Pol II and transcriptional elongation, particularly in the context of HIV-1 transcription, which is heavily dependent on P-TEFb. Depending on the assay applied, Flavopiridol functions at sub-10 nM concentrations, while for other kinases IC_{50} values are much higher. For HIV-1 gene expression regulation that is solely dependent on CYCLIN T1 and CDK9, Ki values of Flavopiridol is ≈ 3 nM, and reported IC_{50} is at the 2–20nM, depending on the applied biochemical kinase assays; therefore, considered as the primary high-affinity inhibitor for targeting CDK9.

Nevertheless, we acknowledge that SR-4835 may also have side effects and inhibit the kinase activity of other CDKs like CDK1, CDK2, CDK4, CDK6. Reported IC_{50} values for these kinases vary in the literature depending on the assay performed. For CDK2 (IC_{50}-100nM), CDK4 and CDK6 (IC_{50} 20-100nM), and CDK7 100-300nM (Carson etal Cancer Research 1996: Chen etal. Blood 2025).

To further strengthen the conclusion that SR-4835-mediated inhibition promotes P-TEFb-mediated gene activation, we followed the reviewer’s suggestion and performed new experiments using AZD-4573, which is a highly selective CDK9 inhibitor (<4nM). Our results confirm the observed results with Flavopiridol. Indeed, treating CD4 T cells with AZD-4573 abolished HIV gene activation effects that are mediated by SR-4835 (Fig. 3B). In addition, as raised by the reviewer, we also employed a genetic approach and knocked down the expression of CDK9 by siRNA (**Fig 3C-E**). Knockdown of CDK9 abolished HIV-1 gene activation that is mediated by SR-4835. We therefore conclude that HIV-1 gene activation that is mediated by SR-4835 is dependent on P-TEFb kinase activity (**Fig. 3C-E**).

3. Figure 4B: Panel 4B should include CCNT1 protein levels, similar to panel A. In addition, other 7SK snRNP components should be examined to more comprehensively support the conclusion that SR-4835 disrupts the inhibitory complex.

We thank the reviewer for this thoughtful suggestion and agree that monitoring the expression of additional components of the 7SKsnRNP will strengthen our hypothesis that SR-4835-mediated gene activation of HIV-1 requires free active P-TEFb. To further confirm this hypothesis, we monitored levels of MEPCE (part of the 7SK snRNP that inhibits P-TEFb kinase activity), following treatment of cells with SR-4835 and immunoprecipitation (IP) with CDK9 IgG. Our analysis demonstrated that association of CDK9 and MEPCE was reduced upon treating cells with SR-4835, pointing to the release of P-TEFb from its inhibitory complex following SR-4835 treatment (**Fig. 4B**). Finally, to further support this conclusion, we monitored association of CDK9 and 7SK snRNA, which is another subunit of the 7SKsnRNP complex. RNA IP experiments used anti-CDK9 IgG to IP CDK9-acssociated partners under control or SR-4835 treatment. qPCR for 7SK snRNA showed that levels of 7SKsnRNA that were associated with CDK9 were reduced upon treating cells with SR-4835 (**Fig 4C**). These new experiments provide a comprehensive conclusion that SR-4835 disrupts the association of P-TEFb with its inhibitory complex and frees P-TEFb for gene activation.

4. Figure 5C: Statistical comparisons between control and siCDK12 conditions are needed to support the conclusion that CDK12 depletion mimics SR-4835 treatment. Importantly, SR-4835 also inhibits CDK13 at the concentrations used. A CDK13 knockdown control should therefore be included to clarify whether the phenotype is specific to CDK12.

We thank the reviewer for this justified comment. As requested, we added the specific statistical analyses to compare the effects on HIV-1 gene activation between control and siCDK12 or between control and siRNA CDK12+CCNK. Moreover, to demonstrate that the activation effects of SR-4835 on HIV-1 activation are dependent on P-TEFb, we genetically depleted CDK9 by siRNA and confirmed the dependency on CDK9 (**Fig 3C**).
In addition, we depleted CDK12, CDK13 and CCNK in CD4 T cells to obtain knockdown of these proteins. We then monitored HIV-1 gene expression and showed a minimal increase (**Fig. 5A +B**). Parallel knockdown of CDK12, CDK13 and CCNK led to an additional slight increase in HIV-1 gene activation (**Fig. 5B**).
In additional new experiments were generated stable cells where either CCNK or CDK12 expression was knocked down using shRNA. In CCNK stable knockdown cells, we then further knockdown CDK12, CDK13 or both with siRNA, and showed minimal increase in HIV-1 gene activation (**Fig. S2**). Furthermore, in CDK12 stable knockdown cells, CDK13 or CCNK depletion or depletion of both proteins also led to elevated levels of HIV-1 gene expression (**Fig S2B**). We thus can conclude that genetic depletion of CDK12, CDK13 or CCNK partially mimics SR-4835-mediated HIV-1 gene activation (Fig 5 and S2).

Of note, our study specifically targets CCNK, the regulatory cyclin subunit of either CDK12 or CDK13. SR-4835 acts by degrading CCNK, disrupting of the CDK12/CDK13-CCNK complex and

depleting the kinases expression (**Fig. 1B**). Thus, the observed inhibitory effects of SR-4835 reflect CCNK degradation rather than direct inhibition of CDK12 or CDK13 kinase activity. Up to date, development of compounds that may discriminate between the closely related kinases CDK12 and CDK13 has not been reported.

5. Figure 6B: The reported statistical significance between the control - latent and control-active groups does not make sense. The difference does not appear substantial in the graph. If the significance is valid, it raises concern that the control condition produces an effect comparable to SR-4835, which would contradict the stated conclusion.
In addition, mean fluorescence intensity (MFI) values should be provided to assess transcriptional activity on a per-cell basis.

We thank the reviewer for raising the important point of statistical significance in **Figure 6B**. We agree that the reported statistical significance between the control latent and control active groups is incorrect and was due to labelling error. We have clarified the experimental design and revised text and figure accordingly to show that the differences between the control latent and control active groups is significant (**supplementary Fig. S3**). This analysis includes the percentage of active HIV-1 ($GFP^+/mKO2^+$), or latent HIV-1 ($GFP\text{-}/mKO2^+$) within an infected population (MKO2+ cells), along with the corresponding GFP mean fluorescence intensity (MFI) from several donors. Overall SR-4835 treatment results in a clear and reproducible activation of HIV-1 gene expression compared to the control conditions.

6. Figure 7A: Statistical testing should be applied to all groups, not only H3K4me3. Without this, conclusions regarding CDK9, Pol II, and Ser2/Ser5 phosphorylation are not sufficiently supported. Additionally, the IgG control appears to show increased signal following SR-4835 treatment. Is this increase statistically significant? If so, how is it explained? This raises concern about potential technical artifacts that should be addressed.

We thank the reviewer for this important suggestion. We agree that statistical analysis should be applied consistently across all datasets. Accordingly, we have now performed statistical analysis between all groups shown in Figure 7A, including CDK9, RNA Polymerase II, and Ser2/Ser5 phosphorylation. The updated statistical analyses have already incorporated into the revised manuscript and the corresponding figure. These analyses further support the conclusions presented in the manuscript. The IgG control signal following SR-4835 treatment shows a minor increase; however, this difference is not statistically significant (**revised Fig. 7**).

Additional experiments were performed to genetically validate activation effects of targeting CDK12/CDK13-CCNK. We monitored HIV-1 gene expression in 2D10 T cells, upon stable depletion of expression of CDK12, or CCNK. In these cells, we documented minor increase in HIV-1 GFP expression. In each of these stable cells, we further depleted the expression of CDK12 and CDK13 or CDK12 and CDK13. Our results show an additional increase in HIV-1 gene activation (**Fig, S2**).

In summary, we again would like to acknowledge the editor and reviewers for reading our manuscript and raise their comments. We are certain that by addressing all of the reviewers' concerns and adding additional new experiments that confirm our conclusions, our work is improved our work, and we hope will be suitable for publication in EMBO Molecular Medicine.

Sincerely
Ran Taube, PhD

References:

1. Victor Quereda, Simon Bayle, Francesca Vena, Sylvia M. Frydman, Andrii Monastyrskyi, William R. Roush, Derek R. Duckett, Therapeutic Targeting of CDK12/CDK13 in Triple-Negative Breast Cancer, Cancer Cell, https://doi.org/10.1016/j.ccell.2019.09.004.

2. Chohan, T. A., Qayyum, A., Rehman, K., Tariq, M., & Akash, M. S. H. (2018). An insight into the emerging role of cyclin-dependent kinase inhibitors as potential therapeutic agents for the treatment of advanced cancers. *Biomedicine & pharmacotherapy = Biomedecine & pharmacotherapie*, *107*, 1326–1341. https://doi.org/10.1016/j.biopha.2018.08.116

3. Bonvini P, Zorzi E, Mussolin L, Monaco G, Pigazzi M, Basso G, Rosolen A. The effect of the cyclin-dependent kinase inhibitor flavopiridol on anaplastic large cell lymphoma cells and relationship with NPM-ALK kinase expression and activity. Haematologica. 2009 Jul;94(7):944-55. doi: 10.3324/haematol.2008.004861. Epub 2009 Jun 16. PMID: 19535344; PMCID: PMC2704305.

11th Sep 2026

Dear Prof. Taube,

Thank you for submitting your revised manuscript to EMBO Molecular Medicine. I am pleased to inform you that we will be able to accept your manuscript pending the following final amendments:

1) Authors: We note name discrepancy in the manuscript and in our submission system: Praveenkumar Murugavelu vs. Praveen Murugavelu. Please correct.
2) Title: Please consider revising the title to better highlight the main findings, e.g. "Targeting CDK12/CDK13 rewires P-TEFb signaling to reverse HIV-1 latency"
3) Abstract: I have gone through your text and revised it (see below). Please review it for accuracy and amend as you see fit:

Persistent HIV-1 latency in long-lived CD4$^+$ T-cell reservoirs remains the major obstacle to viral eradication despite effective antiretroviral therapy (ART). Current latency-reversing agents (LRAs) show limited efficacy, highlighting the need for new therapeutic targets. Here, we identify the CDK12/CDK13-Cyclin K complex as a previously unrecognized regulator of HIV-1 latency. Pharmacological inhibition of CDK12/CDK13 with SR-4835 robustly reactivated latent HIV-1 without additional stimuli, including in primary CD4$^+$ T cells from people living with HIV receiving ART, and synergized with established LRAs to further enhance viral reactivation. Mechanistically, CDK12/CDK13 inhibition promoted the release of P-TEFb from its inhibitory 7SK snRNP complex, resulting in increased RNA polymerase II recruitment, CDK9 activation, and chromatin remodeling that enabled productive HIV-1 transcription. Beyond viral reactivation, these findings reveal a previously unrecognized compensatory interplay between transcriptional kinases that rewires cellular transcriptional programs. Together, our study identifies the CDK12/CDK13-Cyclin K complex as a therapeutically actionable target for HIV-1 latency reversal and provides a mechanistic rationale for improving strategies aimed at achieving an HIV cure.

4) In the main manuscript file, please do the following:
- Please address all comments suggested by our data editors listed below:
o Data availability statement:
1. Please note that the specific URLs for datasets (GSE316746, GSE316689, GSE316989) are not provided in the data availability statement.
o Figure legends:
1. Please define the annotated p values */**/***/**** as well as provide the exact p-values for the same in the legend of figure S2A,C; as appropriate.
2. Please note that the exact p values are not provided in the legends of 1A, C-F; 2A-C; 3A-D; 5A,B; 6C; 7A; 8D; Figure S2B.
3. Please note that information related to n is missing in the legends of figures 4C; 8A; Figure S2A,C; S4D.
4. Please note that the error bars are not defined in the legends of figures 4C; Figure S2A,C; S4D.
5. Please note that scale bar and its definition are missing for figure S4C.
- The manuscript sections should be in the following order: Abstract / The Paper Explained / Introduction / Results / Discussion / Methods / Data Availability / Acknowledgements / Disclosure and Competing Interests Statement / References / Figure Legends / Expanded View Figure Legends.
- Add callouts for Fig 1F, Fig 2D, Fig 3E, Fig 6A.
- In Methods, provide the statement that in addition to the principles set out in the WMA Declaration of Helsinki the experiments involving human subjects also conformed the Department of Health and Human Services Belmont Report.
- Indicate in legends number and nature of replicates and exact p= values, not a range, along with the statistical test used. To keep the figures "clear" some authors found providing an Appendix table Sx with all exact p-values preferable. You are welcome to do this if you want to.
- In Methods, a statistical paragraph should reflect all information that you have filled in the Authors Checklist, especially regarding randomization, blinding, replication.
- Indicate in legends exact n and exact p values, not a range, along with the statistical test used. To keep the figures "clear" some authors found providing an Appendix table Sx with all exact p-values preferable. You are welcome to do this if you want to.
- Add "Disclosure and competing interests statement". We updated our journal's competing interests policy in January 2022 and request authors to consider both actual and perceived competing interests. Please review the policy https://www.embopress.org/competing-interests and update your competing interests if necessary.
- Author contributions: Please remove it from the manuscript and specify author contributions in our submission system. CRediT has replaced the traditional author contributions section because it offers a systematic machine-readable author contributions format that allows for more effective research assessment. You are encouraged to use the free text boxes beneath each contributing author's name to add specific details on the author's contribution. More information is available in our guide to authors:
https://www.embopress.org/page/journal/17574684/authorguide#authorshipguidelines
- Correct the reference citation in the text and reference list. In the text a reference should be cited by author and year of publication. Include a space between a word and the opening parenthesis of the reference that follows. In the reference list, citations should be listed in alphabetical order. Where there are more than 10 authors on a paper, 10 will be listed, followed by

"et al.". DOIs should only be used for preprints and datasets that have not been published yet. Please check "Author Guidelines" for more information.
https://www.embopress.org/page/journal/17574684/authorguide#referencesformat
- In data availability statement please remove paragraph about data analysis and place it in the Methods section under the subheading "Data analysis". Please use the following format to report the accession number of your data:

The datasets produced in this study are available in the following databases:
[data type]: [full name of the resource] [accession number/identifier] ([doi or URL or identifiers.org/DATABASE:ACCESSION])

Please check "Author Guidelines" for more information.
https://www.embopress.org/page/journal/17574684/authorguide#availabilityofpublishedmaterial
5) Funding: Make sure that information about all sources of funding are complete in both our submission system and in the manuscript. Currently, Germany's Excellence Strategy (EXC 3018/1 - 533587280 (SynthImmune) to O.T.F.); the DFG-Emmy Noether Program; (Project No.495793173 to P.S.), and the German Center for Infection Research (O.T.F., P.S.) are missing in our system. Please correct.
6) The Paper Explained: Please provide "The Paper Explained" and add it to the main manuscript text. Please check any of recently published EMBO Mol Med research articles for reference.
7) Synopsis:
- Synopsis image: Please provide a visual abstract as a high-resolution .jpeg file exactly 550 px-wide x 300 pixels high to illustrate your article.
- Synopsis text: Please provide a short standfirst (maximum of 300 characters, including space) as well as 2-5 one sentence bullet points that summarize the paper as a .doc file. Please write the bullet points to summarize the key NEW findings. They should be designed to be complementary to the abstract - i.e. not repeat the same text. We encourage inclusion of key acronyms and quantitative information (maximum of 30 words / bullet point). Please use the passive voice.
- Please check your synopsis text and image before submission with your revised manuscript. Please be aware that in the proof stage minor corrections only are allowed (e.g., typos).
8) As part of the EMBO Publications transparent editorial process (see our Editorial at http://embomolmed.embopress.org/content/2/9/329), EMBO Molecular Medicine will publish online a Review Process File (RPF) to accompany accepted manuscripts. This file will be published in conjunction with your paper and will include the anonymous referee reports, your point-by-point response and all pertinent correspondence relating to the manuscript. Let us know if you want to remove or not any figures from it prior to publication. Please note that the Authors checklist will be published at the end of the RPF.
9) Please provide a point-by-point letter INCLUDING my comments as well as the reviewer's reports and your detailed responses (as Word file).

I look forward to reading a new revised version of your manuscript as soon as possible.

Yours sincerely,

Zeljko Durdevic

Zeljko Durdevic
Senior Editor
EMBO Molecular Medicine

*** Instructions to submit your revised manuscript ***

*** PLEASE NOTE *** As part of the EMBO Publications transparent editorial process initiative (see our Editorial at https://www.embopress.org/doi/pdf/10.1002/emmm.201000094), EMBO Molecular Medicine will publish online a Review Process File to accompany accepted manuscripts.

In the event of acceptance, this file will be published in conjunction with your paper and will include the anonymous referee reports, your point-by-point response and all pertinent correspondence relating to the manuscript. If you do NOT want this file to be published, please inform the editorial office at contact@embomolmed.org.

*****

When preparing your revised manuscript, please refer to our guidelines: https://link.springer.com/journal/44321/submission-guidelines#cms-Revised-submissions. We perform an initial quality control of all revised manuscripts before re-review; failure to include requested items will delay the evaluation of your revision.

We require:

1) A .docx formatted version of the manuscript text (including legends for main figures, EV figures and tables). Please make sure that the changes are highlighted to be clearly visible.

2) Individual production quality figure files as .eps, .tif, .jpg (one file per figure). For guidance, download the 'Figure Guide PDF': https://media.springernature.com/original/springer-cms/rest/v1/content/27825798/data/v1.

3) A .docx formatted letter INCLUDING the reviewers' reports and your detailed point-by-point responses to their comments. As part of the EMBO Press transparent editorial process, the point-by-point response is part of the Review Process File (RPF), which will be published alongside your paper.

4) A complete author checklist, which you can download from our author guidelines. Please insert information in the checklist that is also reflected in the manuscript. The completed author checklist will also be part of the RPF.

5) Please note that all corresponding authors are required to supply an ORCID ID for their name upon submission of a revised manuscript.

6) It is mandatory to include a 'Data Availability' section after the Materials and Methods. Before submitting your revision, primary datasets produced in this study need to be deposited in an appropriate public database, and the accession numbers and database listed under 'Data Availability'. Please remember to provide a reviewer password if the datasets are not yet public.

In case you have no data that requires deposition in a public database, please state so in this section. Note that the Data Availability Section is restricted to new primary data that are part of this study.

7) For data quantification: please specify the name of the statistical test used to generate error bars and P values, the number (n) of independent experiments (specify technical or biological replicates) underlying each data point and the test used to calculate p-values in each figure legend. The figure legends should contain a basic description of n, P and the test applied. Graphs must include a description of the bars and the error bars (s.d., s.e.m.).

8) At EMBO Press we ask authors to provide source data for the main manuscript figures. You will receive a separate email with instructions for providing source data with your revised manuscript, including how to upload and organize the files.

9) Our journal encourages inclusion of *data citations in the reference list* to directly cite datasets that were re-used and obtained from public databases. Data citations in the article text are distinct from normal bibliographical citations and should directly link to the database records from which the data can be accessed. In the main text, data citations are formatted as follows: "Data ref: Smith et al, 2001" or "Data ref: NCBI Sequence Read Archive PRJNA342805, 2017". In the Reference list, data citations must be labeled with "[DATASET]". A data reference must provide the database name, accession number/identifiers and a resolvable link to the landing page from which the data can be accessed at the end of the reference.

10) We replaced Supplementary Information with Expanded View (EV) Figures and Tables that are collapsible/expandable online. A maximum of 5 EV Figures can be typeset. EV Figures should be cited as 'Figure EV1, Figure EV2" etc... in the text and their respective legends should be included in the main text after the legends of regular figures.

- For the figures that you do NOT wish to display as Expanded View figures, they should be bundled together with their legends in a single PDF file called *Appendix*, which should start with a short Table of Content. Appendix figures should be referred to in the main text as: "Appendix Figure S1, Appendix Figure S2" etc.

- Additional Tables/Datasets should be labeled and referred to as Table EV1, Dataset EV1, etc. Legends have to be provided in a separate tab in case of .xls files. Alternatively, the legend can be supplied as a separate text file (README) and zipped together with the Table/Dataset file.

11) The paper explained: EMBO Molecular Medicine articles are accompanied by a summary of the articles to emphasize the major findings in the paper and their medical implications for the non-specialist reader. Please provide a draft summary of your article highlighting

- the medical issue you are addressing,

- the results obtained and

- their clinical impact.

This may be edited to ensure that readers understand the significance and context of the research. Please refer to any of our published articles for an example.

12) Author contributions: You will be asked to provide CRediT (Contributor Role Taxonomy) terms in the submission system. These replace a narrative author contribution section in the manuscript.

13) A Conflict of Interest statement should be provided in the main text.

14) Every published paper includes a 'Synopsis' to further enhance discoverability. Synopses are displayed on the journal webpage and are freely accessible to all readers. They include a short stand first (maximum of 300 characters, including space) as well as 2-5 one-sentences bullet points that summarizes the paper. Please write the bullet points to summarize the key NEW findings. They should be designed to be complementary to the abstract - i.e. not repeat the same text. We encourage inclusion of key acronyms and quantitative information (maximum of 30 words / bullet point). Please use the passive voice. Please attach these in a separate file or send them by email, we will incorporate them accordingly.

Please also suggest a striking image or visual abstract to illustrate your article as a PNG file 550 px wide x 300 px high.

15) Include a Reagents and Tools Table as part of the Methods section, which can be downloaded from our author guidelines.

EMBO Molecular Medicine has a "scooping protection" policy, whereby similar findings that are published by others during review or revision are not a criterion for rejection. Should you decide to submit a revised version, I do ask that you get in touch after three months if you have not completed it, to update us on the status.

*Additional important information regarding Figures

Each figure should be given in a separate file and should have the following resolution:
Graphs 800-1,200 DPI
Photos 400-800 DPI
Colour (only CMYK) 300-400 DPI"

Figures are not edited by the production team. All lettering should be the same size and style; figure panels should be indicated by capital letters (A, B, C etc). Gridlines are not allowed except for log plots. Figures should be numbered in the order of their appearance in the text with Arabic numerals. Each Figure must have a separate legend and a caption is needed for each panel.

*Additional important information regarding figures and illustrations can be found at
https://media.springernature.com/original/springer-cms/rest/v1/content/27825798/data/v1

***** Reviewer's comments *****

Referee #1 (Remarks for Author):

The authors have responded to my comments.

Referee #2 (Remarks for Author):

The authors did a good job to add additional J-Lat clone, primary macrophage/U1 studies, primary-cell cART experiments, viral-splicing analysis, Bliss analysis, AZD-4573 and CDK9 knockdown, additional 7SK snRNP assays, and CDK13 genetic perturbation assays. We especially appreciate the CDK9-KO and AZD4573 experiments supporting P-TEFb as the downstream effector of SR-4835. The authors have addressed all our concerns and the paper is acceptable as is.

Referee #3 (Comments on Novelty/Model System for Author):

The revised manuscript has been improved and the authors replied to all my comments.

Referee #3 (Remarks for Author):

The authors have satisfactorily addressed my comments, and the revised manuscript is clear and substantially improved. The study provides important mechanistic insights into HIV-1 latency reversal and merits publication in EMBO Molecular Medicine. I recommend acceptance, potentially as a Short Report if space is limited.

All minor editorial requests have been addressed by the authors.

2nd Revision - Editorial Decision 22nd Sep 2026

22nd Sep 2026

Dear Prof. Taube,

We are pleased to inform you that your manuscript is accepted for publication and is now being sent to our publisher to be included in the next available issue of EMBO Molecular Medicine.

Your manuscript will be processed for publication by EMBO Press. It will be copy edited and you will receive page proofs prior to publication. Please note that you will be contacted by Springer Nature Author Services to complete licensing and payment information.

You may qualify for financial assistance for your publication charges - either via a Springer Nature fully open access agreement or an EMBO initiative. Check your eligibility: https://link.springer.com/journal/44321/how-to-publish-with-us

Should you be planning a Press Release on your article, please notify the production team via this form in order to coordinate publication and release dates: https://forms.cloud.microsoft/e/eH4A8JVP6g

If you have any questions, please do not hesitate to contact the Editorial Office. Thank you for your contribution to EMBO Molecular Medicine.

Yours sincerely,
Zeljko Durdevic

Zeljko Durdevic
Senior Editor
EMBO Molecular Medicine

>>> Please note that it is EMBO Molecular Medicine policy for the transcript of the editorial process (containing referee reports and your response letter) to be published as an online supplement to each paper. If you do NOT want this, you will need to inform the Editorial Office via email immediately. More information is available here: https://link.springer.com/partners/embo-press/editorial-policies#Peer%20review

EMBO Press Author Checklist

Corresponding Author Name: Ran Taube	
Journal Submitted to: EMBO Molecular Medicine	
Manuscript Number: EMM-2026-23779	

USEFUL LINKS FOR COMPLETING THIS FORM
The EMBO Journal - Author Guidelines
EMBO Reports - Author Guidelines
Molecular Systems Biology - Author Guidelines
EMBO Molecular Medicine - Author Guidelines

Reporting Checklist for Life Science Articles (updated January
This checklist is adapted from Materials Design Analysis Reporting (MDAR) Checklist for Authors. MDAR establishes a minimum set of requirements in transparent reporting in the life sciences (see Statement of Task: 10.31222/osf.io/9sm4x). Please follow the journal's guidelines in preparing your
Please note that a copy of this checklist will be published alongside your article.

Abridged guidelines for figures
1. Data
The data shown in figures should satisfy the following conditions:
- the data were obtained and processed according to the field's best practice and are presented to reflect the results of the experiments in an accurate and unbiased manner.
- ideally, figure panels should include only measurements that are directly comparable to each other and obtained with the same assay.
- plots include clearly labeled error bars for independent experiments and sample sizes. Unless justified, error bars should not be shown for technical
- if n<5, the individual data points from each experiment should be plotted. Any statistical test employed should be justified.
- Source Data should be included to report the data underlying figures according to the guidelines set out in the authorship guidelines on Data

2. Captions
Each figure caption should contain the following information, for each panel where they are relevant:
- a specification of the experimental system investigated (eg cell line, species name).
- the assay(s) and method(s) used to carry out the reported observations and measurements.
- an explicit mention of the biological and chemical entity(ies) that are being measured.
- an explicit mention of the biological and chemical entity(ies) that are altered/varied/perturbed in a controlled manner.
- the exact sample size (n) for each experimental group/condition, given as a number, not a range;
- a description of the sample collection allowing the reader to understand whether the samples represent technical or biological replicates (including how many animals, litters, cultures, etc.).
- a statement of how many times the experiment shown was independently replicated in the laboratory.
- definitions of statistical methods and measures:
  - common tests, such as t-test (please specify whether paired vs. unpaired), simple χ2 tests, Wilcoxon and Mann-Whitney tests, can be unambiguously identified by name only, but more complex techniques should be described in the methods section;
  - are tests one-sided or two-sided?
  - are there adjustments for multiple comparisons?
  - exact statistical test results, e.g., P values = x but not P values < x;
  - definition of 'center values' as median or average;
  - definition of error bars as s.d. or s.e.m.

Please complete ALL of the questions below.
Select "Not Applicable" only when the requested information is not relevant for your study.

Materials

Newly Created Materials	Information included in the manuscript?	In which section is the information available? (Reagents and Tools Table, Materials and Methods, Figures, Data Availability Section)
New materials and reagents need to be available; do any restrictions apply?	Yes	Materials and Methods and Reagent Table

Antibodies	Information included in the manuscript?	In which section is the information available? (Reagents and Tools Table, Materials and Methods, Figures, Data Availability Section)
For **antibodies** provide the following information:<br>- Commercial antibodies: RRID (if possible) or supplier name, catalogue number and or/clone number<br>- Non-commercial: RRID or citation	Yes	Materials and Methods and Reagent Table

DNA and RNA sequences	Information included in the manuscript?	In which section is the information available? (Reagents and Tools Table, Materials and Methods, Figures, Data Availability Section)
**Short novel DNA or RNA including primers, probes**: provide the sequences.	Yes	Materials and Methods and Reagent Table

Cell materials	Information included in the manuscript?	In which section is the information available? (Reagents and Tools Table, Materials and Methods, Figures, Data Availability Section)
**Cell lines:** Provide species information, strain. Provide accession number in repository **OR** supplier name, catalog number, clone number, and/**OR** RRID.	Yes	Materials and Methods and Reagent Table
**Primary cultures:** Provide species, strain, sex of origin, genetic modification status.	Yes	Materials and Methods and Reagent Table
Report if the cell lines were recently **authenticated** (e.g., by STR profiling) and tested for mycoplasma contamination.	Not Applicable	

Experimental animals	Information included in the manuscript?	In which section is the information available? (Reagents and Tools Table, Materials and Methods, Figures, Data Availability Section)
**Laboratory animals or Model organisms:** Provide species, strain, sex, age, genetic modification status. Provide accession number in repository **OR** supplier name, catalog number, clone number, **OR** RRID.	Not Applicable	
**Animal observed in or captured from the field:** Provide species, sex, and age where possible.	Not Applicable	
Please detail **housing and husbandry conditions**.	Not Applicable	

Plants and microbes	Information included in the manuscript?	In which section is the information available? (Reagents and Tools Table, Materials and Methods, Figures, Data Availability Section)
**Plants:** provide species and strain, ecotype and cultivar where relevant, unique accession number if available, and source (including location for collected wild specimens).	Not Applicable	
**Microbes:** provide species and strain, unique accession number if available, and source.	Not Applicable	

Human research participants	Information included in the manuscript?	In which section is the information available? (Reagents and Tools Table, Materials and Methods, Figures, Data Availability Section)
If collected and within the bounds of privacy constraints report on age, sex and gender or ethnicity for all study participants.	Yes	Materials and Methods and supplementary Table 1

Core facilities	Information included in the manuscript?	In which section is the information available? (Reagents and Tools Table, Materials and Methods, Figures, Data Availability Section)
If your work benefited from core facilities, was their service mentioned in the acknowledgments section?	Yes	Materials and Methods and Reagent Table

Design

Study protocol	Information included in the manuscript?	In which section is the information available? (Reagents and Tools Table, Materials and Methods, Figures, Data Availability Section)
If study protocol has been **pre-registered, provide DOI in the manuscript**. For clinical trials, provide the trial registration number **OR** cite DOI.	Not Applicable	
Report the **clinical trial registration number** (at ClinicalTrials.gov or equivalent), where applicable.	Yes	

Laboratory protocol	Information included in the manuscript?	In which section is the information available? (Reagents and Tools Table, Materials and Methods, Figures, Data Availability Section)
Provide DOI OR other citation details if **external detailed step-by-step protocols** are available.	Not Applicable	

Experimental study design and statistics	Information included in the manuscript?	In which section is the information available? (Reagents and Tools Table, Materials and Methods, Figures, Data Availability Section)
Include a statement about **sample size** estimate even if no statistical methods were used.	Yes	Materials and Methods and Reagent Table
Were any steps taken to minimize the effects of subjective bias when allocating animals/samples to treatment (e.g. **randomization procedure**)? If yes, have they been described?	Not Applicable	
Include a statement about **blinding** even if no blinding was done.	Not Applicable	
Describe **inclusion/exclusion criteria** if samples or animals were excluded from the analysis. Were the criteria pre-established?<br><br>If sample or data points were omitted from analysis, report if this was due to attrition or intentional exclusion and provide justification.	Not Applicable	
For every figure, are **statistical tests** justified as appropriate? Do the data meet the assumptions of the tests (e.g., normal distribution)? Describe any methods used to assess it. Is there an estimate of variation within each group of data? Is the variance similar between the groups that are being statistically compared?	Yes	Information is included to each Figure.

Sample definition and in-laboratory replication	Information included in the manuscript?	In which section is the information available? (Reagents and Tools Table, Materials and Methods, Figures, Data Availability Section)
In the figure legends: state number of times the experiment was **replicated** in laboratory.	Yes	Results and Figure Legends
In the figure legends: define whether data describe **technical or biological replicates**.	Yes	Results and Figure Legends

Ethics

Ethics	Information included in the manuscript?	In which section is the information available? (Reagents and Tools Table, Materials and Methods, Figures, Data Availability Section)
Studies involving **human participants**: State details of **authority granting ethics approval** (IRB or equivalent committee(s), provide reference number for approval.	Yes	Refer to the material and Methods specidying work with human participants
Studies involving **human participants**: Include a statement confirming that **informed consent** was obtained from all subjects and that the experiments conformed to the principles set out in the WMA Declaration of Helsinki and the Department of Health and Human Services Belmont Report.	Yes	Refer to the material and Methods specidying work with human participants
Studies involving **human participants:** For publication of **patient photos**, include a statement confirming that consent to publish was obtained.	Not Applicable	
Studies involving experimental **animals**: State details of **authority granting ethics approval** (IRB or equivalent committee(s), provide reference number for approval. Include a statement of compliance with ethical regulations.	Not Applicable	
Studies involving **specimen and field samples:** State if relevant **permits** obtained, provide details of authority approving study; if none were required, explain why.	Not Applicable	

Dual Use Research of Concern (DURC)	Information included in the manuscript?	In which section is the information available? (Reagents and Tools Table, Materials and Methods, Figures, Data Availability Section)
Could your study fall under dual use research restrictions? Please check biosecurity documents and list of **select agents and toxins** (CDC): https://www.selectagents.gov/sat/list.htm	Not Applicable	
If you used a select agent, is the security level of the lab appropriate and reported in the manuscript?	Not Applicable	
If a study is subject to dual use research of concern regulations, is the name of the **authority granting approval and reference number** for the regulatory approval provided in the manuscript?	Not Applicable	

Reporting

The MDAR framework recommends adoption of discipline-specific guidelines, established and endorsed through community initiatives. Journals have their own policy about requiring specific guidelines and recommendations to complement MDAR.

Adherence to community standards	Information included in the manuscript?	In which section is the information available? (Reagents and Tools Table, Materials and Methods, Figures, Data Availability Section)
State if relevant guidelines or checklists (e.g., **ICMJE, MIBBI, ARRIVE, PRISMA**) have been followed or provided.	Not Applicable	
For **tumor marker prognostic studies,** we recommend that you follow the **REMARK** reporting guidelines (see link list at top right). See author guidelines, under 'Reporting Guidelines'. Please confirm you have followed these guidelines.	Not Applicable	
For **phase II and III randomized controlled trials**, please refer to the **CONSORT** flow diagram (see link list at top right) and submit the CONSORT checklist (see link list at top right) with your submission. See author guidelines, under 'Reporting Guidelines'. Please confirm you have submitted this list.	Not Applicable	

Data Availability

Data availability	Information included in the manuscript?	In which section is the information available? (Reagents and Tools Table, Materials and Methods, Figures, Data Availability Section)
Have **primary datasets** been deposited according to the journal's guidelines (see 'Data Deposition' section) and the respective accession numbers provided in the Data Availability Section?	Yes	see materials and Methods and Results sections
Were **human clinical and genomic datasets** deposited in a public access-controlled repository in accordance to ethical obligations to the patients and to the applicable consent agreement?	Yes	see materials and Methods and Results sections
Are **computational models** that are central and integral to a study available without restrictions in a machine-readable form? Were the relevant accession numbers or links provided?	Not Applicable	
If publicly available data were reused, provide the respective **data citations in the reference list**.	Not Applicable	
