# Supplementary material for: Inhibition of CDK12/CDK13-CYCLIN K rewires P-TEFb signaling to reverse HIV-1 latency and modulate cellular gene expression
Source: EMBO Mol Med. 2026 Oct 6;18(11):4558–84. doi: 10.1038/s44321-026-00536-z (PMC13748670; doi:10.1038/s44321-026-00536-z)
Supplement: Supplementary file 3 [file 44321_2026_536_MOESM3_ESM.pdf]

Expanded View Figures

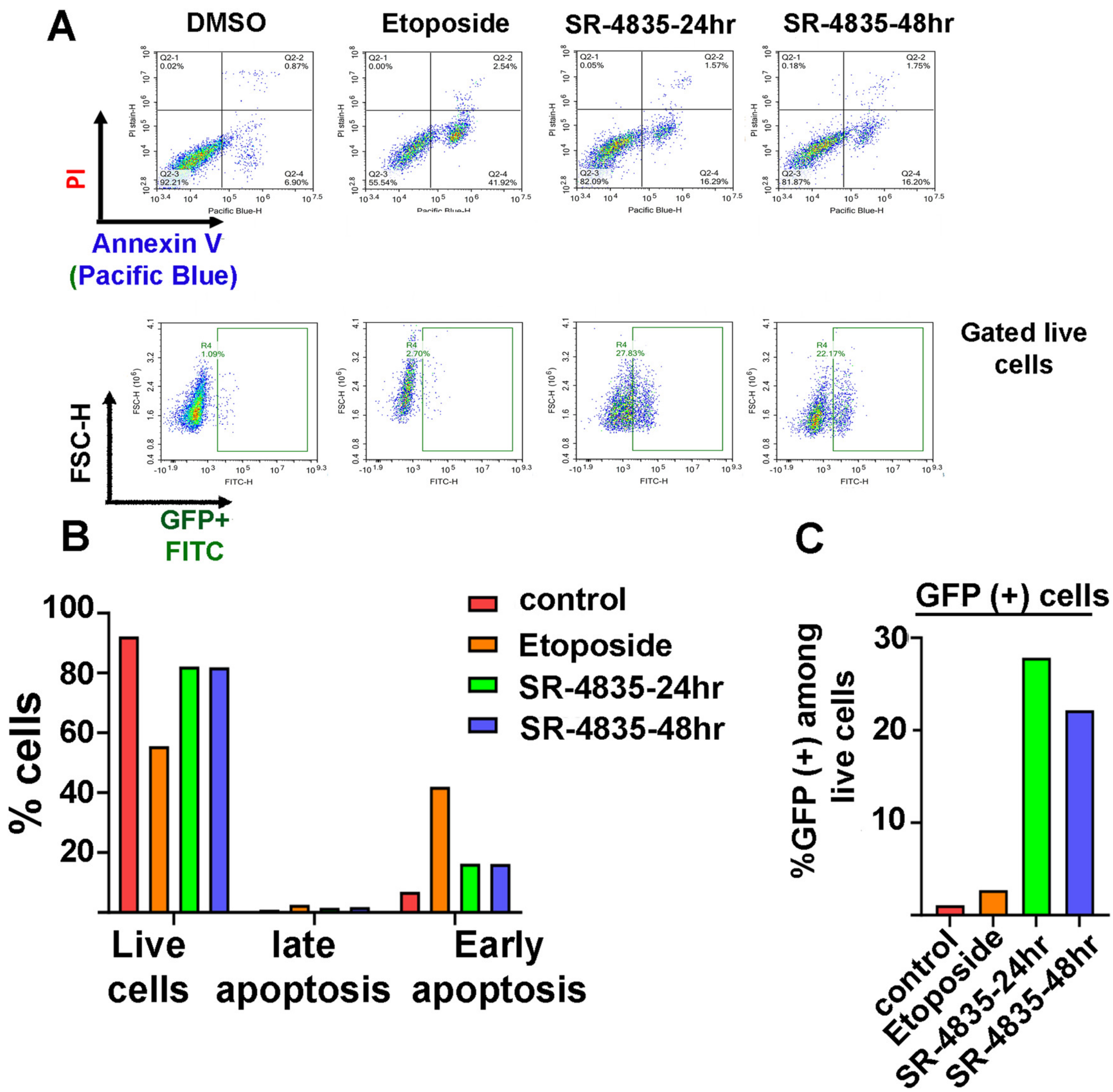

Figure EV1. Targeting CDK12/CDK13-CCNK with SR-4835 induces minimal cell apoptosis.

Cell apoptosis was monitored in 2D10 cells using Annexin V-Pacific Blue and Propidium Iodide (PI) staining. Cells were treated with SR-4835 for 2 h, and at the indicated time points were harvested and stained for Annexin V-pacific blue and PI before being analyzed by FACS. (**A**) FACS dot plots after 24 and 48 h post-treatment with SR-4835. Treated cells were analyzed by flow cytometry and gated only on live cells (Annexin V(-)/PI(-)), excluding apoptotic or necrotic perturbations in treated cells. Etoposide-treated cells (10 mM) served as a positive control for cell apoptosis. (**B**) Quotative analysis of FACS plots in each category; live cells (Annexin V-/ PI-); early apoptotic (Annexin V-/ PI+); Late apoptosis (Annexin V+/ PI+). (**C**) Quotative analysis of HIV-GFP expression following treatment of cells with SR-4835. Gated only on live cells.

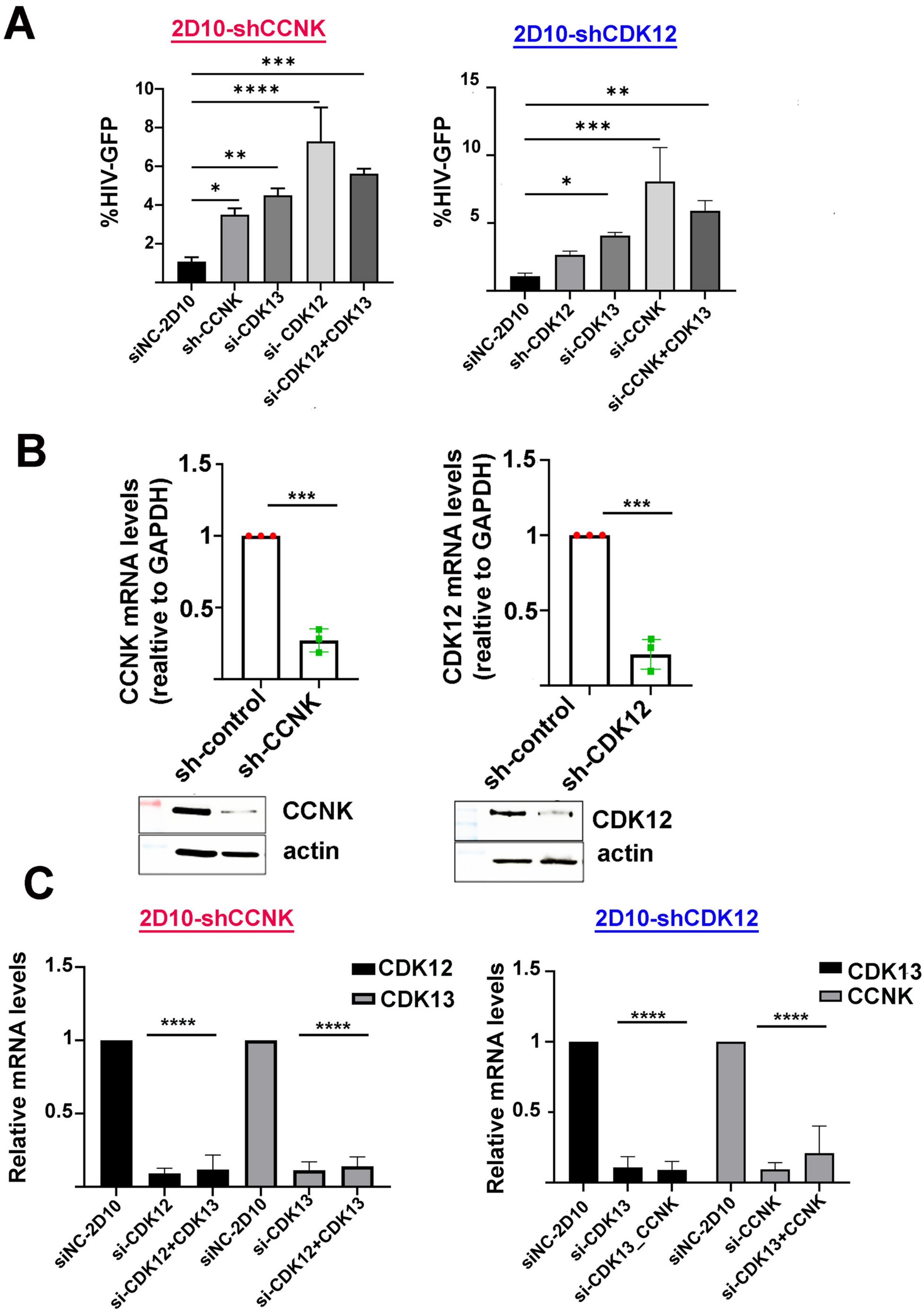
A
2D10-shCCNK
%HIV-GFP
10
8
6
4
2
*
**
****
***
siNC-2D10
sh-CCNK
si-CDK13
si- CDK12
si-CDK12+CDK13
2D10-shCDK12
%HIV-GFP
15
10
5
*
***
**
siNC-2D10
sh-CDK12
si-CDK13
si-CCNK
si-CCNK+CDK13
B
CCNK mRNA levels (realtive to GAPDH)
1.5
1
0.5
***
sh-control
sh-CCNK
CCNK
actin
CDK12 mRNA levels (realtive to GAPDH)
1.5
1
0.5
***
sh-control
sh-CDK12
CDK12
actin
C
2D10-shCCNK
Relative mRNA levels
1.5
1
0.5
CDK12
CDK13
****
****
siNC-2D10
si-CDK12
si-CDK12+CDK13
siNC-2D10
si-CDK13
si-CDK12+CDK13
2D10-shCDK12
Relative mRNA levels
1.5
1
0.5
CDK13
CCNK
****
****
siNC-2D10
si-CDK13
si-CDK13_CCNK
siNC-2D10
si-CCNK
si-CDK13+CCNK

 © The Author(s)

Figure EV2. Genetic Targeting of CDK12/CDK13-CCNK mimics SR-4835-mediated HIV-1 activation.

(**A**) 2D10 CD4 T cells were transduced with lentivirus expressing shRNA targeting CCNK or CDK12. Cells were selected with puromycin to achieve stable knockdown of CCNK or CDK12. Stable cells were then electroporated with the indicated siRNA (total concentration 100 nM). 48 h post electroporation, HIV-1-GFP expression was monitored by FACS to document HIV-1 gene activation. Data are presented as mean ± SD from $n = 3$ independent biological replicates. Statistical significance was determined using one-way ANOVA. Left Panel: $P = 0.0172$: siNC vs shCCNK, $P = 0.0011$: siNC vs siCDK13, $P < 0.0001$: siNC vs siCDK12 and $P = 0.0002$: siNC vs siCDK12+siCDK13. Right Panel: $P = 0.0446$: siNC vs siCDK13, $P = 0.0001$: siNC vs siCCNK and $P = 0.0020$: siNC vs siCDK13+siCCNK. (**B**) qPCR analysis and western blot confirm CCNK or CDK12 depletion. Relative mRNA expression of CDK12 and CCNK was quantified by RT-qPCR in stable shCCNK-2D10 or shCDK12-2D10 clonal cell lines. Transcript levels are normalized to the control 2D10 which was set to 1. Data are presented as mean ± SD from three independent biological replicates. Statistical significance was determined using two-way ANOVA. ****$P = 0.0001$. (**C**) qPCR confirm siRNA-specific knockdown of CCNK, CDK12, CDK13 or combination in clonal sh-CDK12 or sh-CCNK cells. Data are presented as mean ± SD from $n = 3$ independent biological replicates. Statistical significance was determined using One-way ANOVA. ****$P = 0.0001$.

© The Author(s)

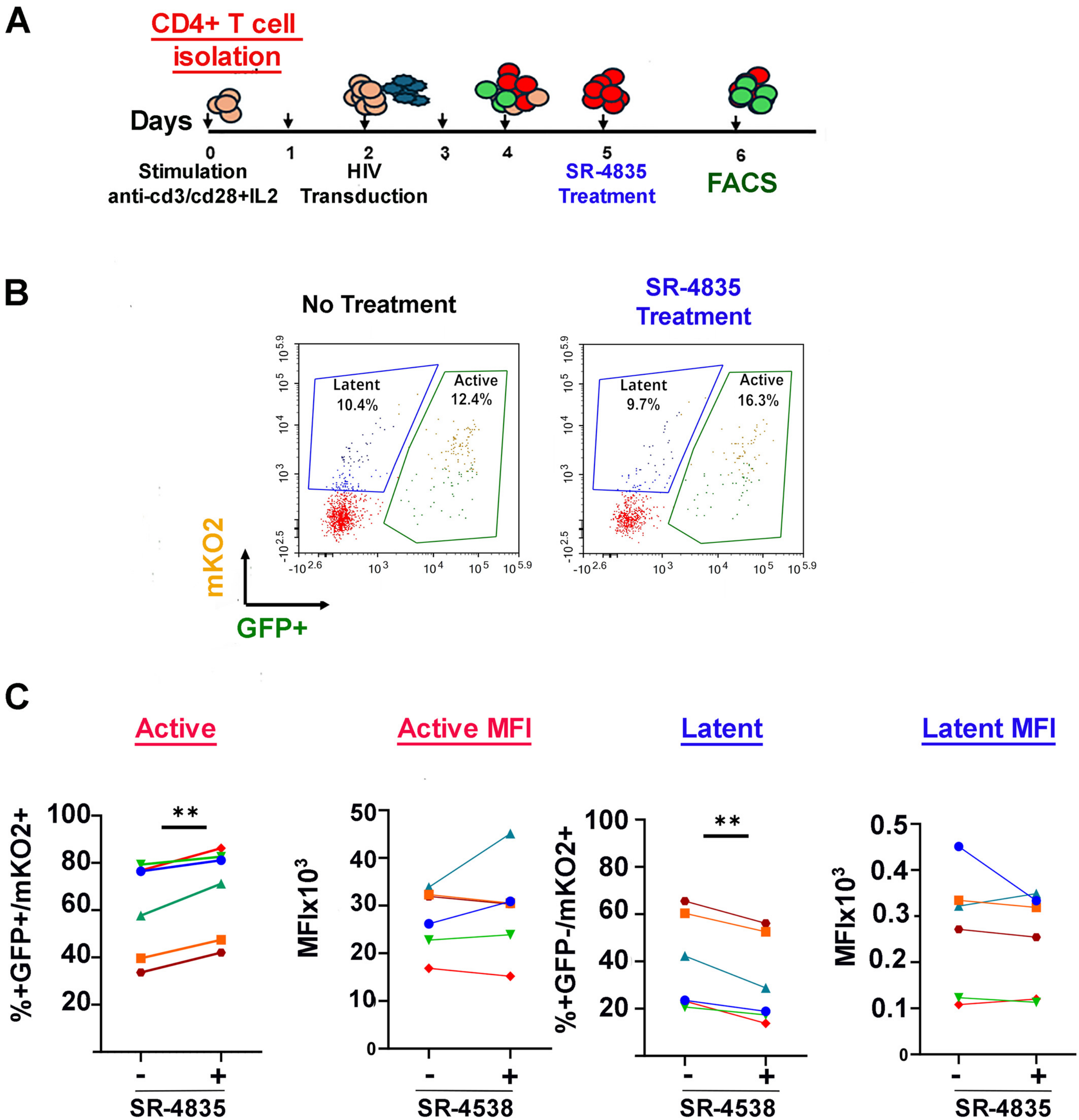

Figure EV3. SR-4835 CDK12/CDK13-CCNK inhibitor reverses HIV-1 latency in primary CD4 + T cells.

(**A**) Schematic showing experimental design for isolation, activation, HIV-1_{GKO} transduction and SR-4835 treatment of primary CD4 + T cells. (**B**) SR-4835 treatment increases the proportion of GFP⁺ cells, indicating reactivation of latent HIV-1. Representative FACS plots show identification and characterization of latent (GFP-/mKO2⁺) and active (GFP + /mKO2⁺) HIV-1 in primary cell populations in the presence or absence of SR-4835. (**C**) Quantification of HIV-1 reactivation in primary CD4 + T cells shown as the percentage of active (GFP⁺/mKO2⁺) cells or (latent (GFP-/mKO2⁺) HIV-1 within the infected population (MKO2+ cells), along with the corresponding GFP mean fluorescence intensity (MFI), reflecting the level of HIV-1 transcription per active cell. Primary CD4⁺ T cells from independent donors ($n = 6$) were analyzed across treatment conditions. Each dot represents an individual donor, with paired samples connected by lines. Data were normalized to the mKO2⁺ population, and statistical significance was determined using a paired Student's t-test (**$P = 0.003$).

© The Author(s)

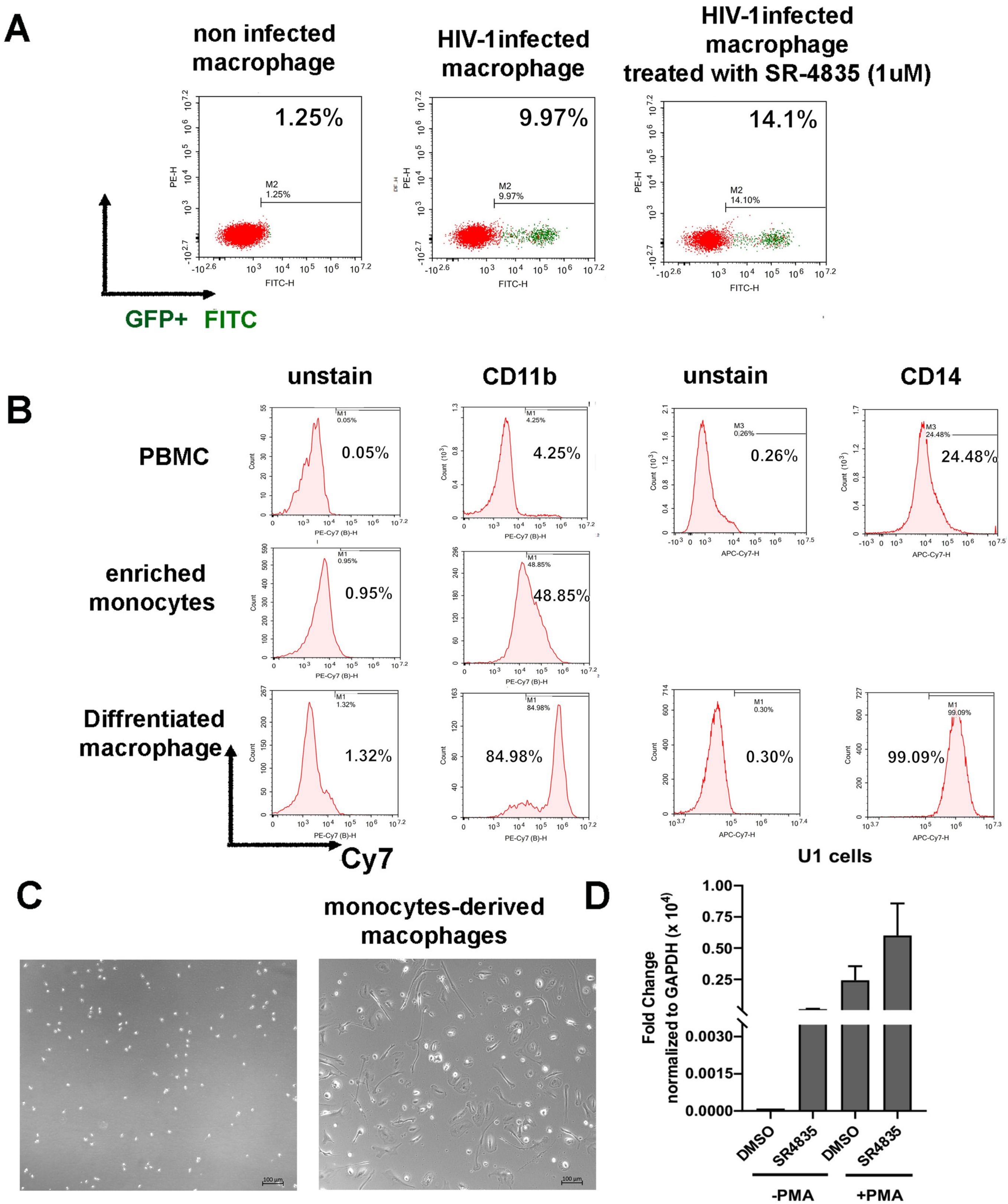
A
non infected macrophage
HIV-1infected macrophage
HIV-1infected macrophage treated with SR-4835 (1uM)
1.25%
9.97%
14.1%
M2 1.25%
M2 9.97%
M2 14.10%
PE-H
FITC-H
GFP+ FITC
B
unstain
CD11b
unstain
CD14
PBMC
enriched monocytes
Diffrentiated macrophage
0.05%
4.25%
0.26%
24.48%
0.95%
48.85%
1.32%
84.98%
0.30%
99.09%
Count
PE-Cy7 (B)-H
APC-Cy7-H
Cy7
C
monocytes-derived macophages
100 µm
D
U1 cells
Fold Change normalized to GAPDH (x 10^4)
1.00
0.75
0.50
0.25
0.0030
0.0015
0.0000
DMSO
SR4835
DMSO
SR4835
-PMA
+PMA

Figure EV4. Effects of SR-4835-mediated inhibition of CDK12/CDK13 on human primary monocyte derived macrophages.

(**A**) Monocytes were isolated from PBMCs using the "EasySep enrichment kit" (Stemcell Technologies). Cells were differentiated for 5 days with GM-CSF and then stained with aCD11b PE-Cy7 conjugated antibody (Bio-Legend) for characterization. 24 h post stimulation, cells were transduced with HIV-1 NLENG at low MOI. At day 6 post stimulation, cells were treated with 0.5 mM SR-4835 for 2 h and washed before being analyzed by FACS for HIV-1 GFP expression. (**B**) Cells were stained with CD11 b or CD14 by FACS for characterizing monocyte-derived macrophages. (**C**) Monocyte-derived macrophages exhibit a distinct phenotype. Monocytes were isolated from human PBMCs using "EasySep enrichment cocktail" (Stem Cells Technology) and stimulated with 50 ng/ml of GM-CSF (Miltenyi Biotec) for 8 days before imaging by light microscope. (**D**) U1 human monocytes cells that are infected with HIV-1 were cultured under standard conditions and treated with the indicated concentrations of SR-4835 (0.5 mM) in either the absence or presence of PMA/Ionomycin (200 nM/1 mM) or 48 h ($n = 3$ independent biological replicates. Following treatment, total RNA was extracted using an RNA isolation kit according to the manufacturer's instructions. Equal amounts of RNA were reverse transcribed into cDNA using a reverse transcription kit. Quantitative PCR was performed using gene-specific primers targeting the HIV-1 unspliced transcripts. Relative HIV transcript levels were calculated using the $2^{-\Delta\Delta Ct}$ method and expressed as fold change relative to GAPDH and to the DMSO-treated control. Data are presented as mean ± SD.

 © The Author(s)

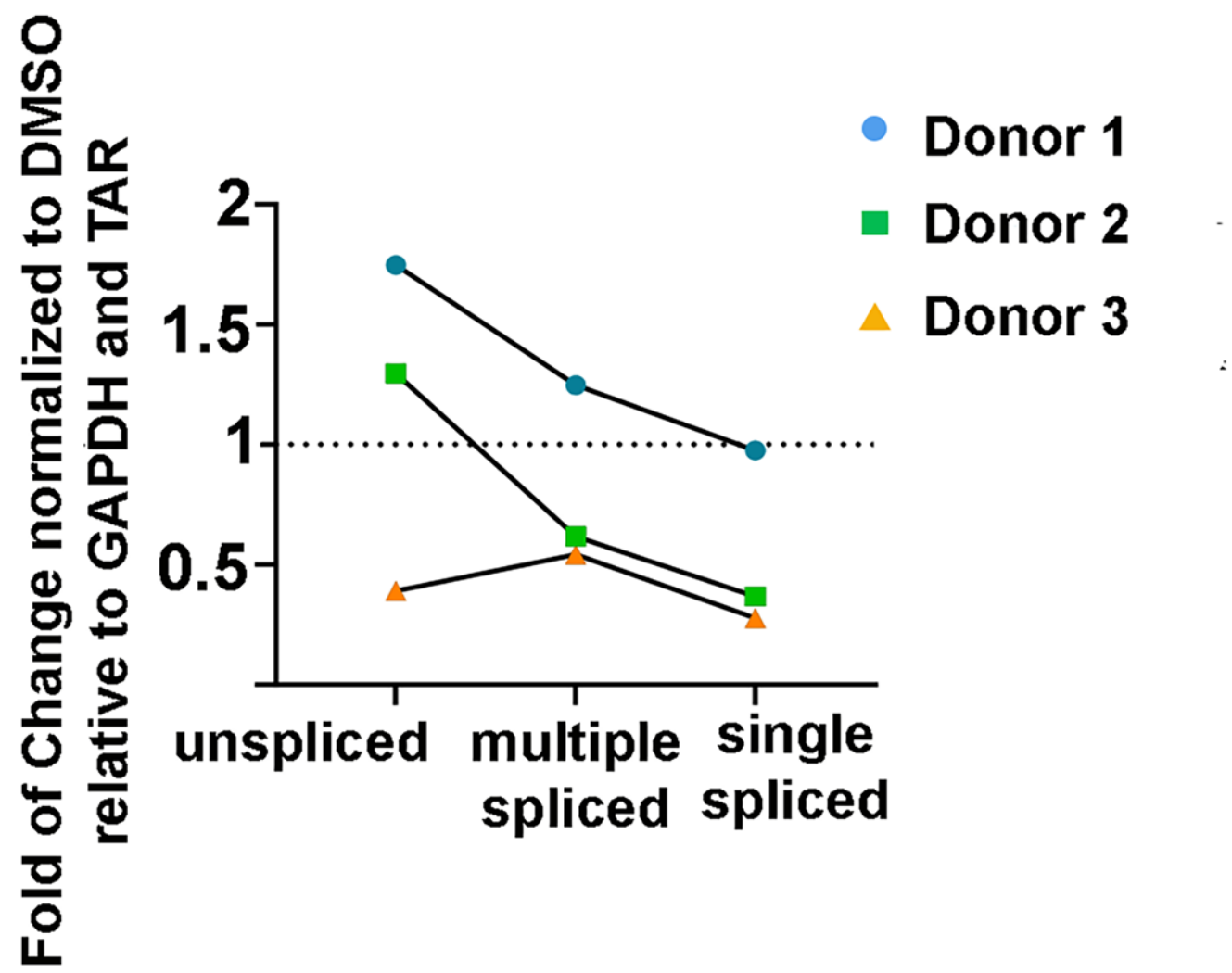

Figure EV5. SR-4835 CDK12/CDK13-CCNK SR-4835 inhibitor has minimal effects on HIV-1 gene splicing.

Primary CD4 T cells were isolated from naïve PBMCs from 3 donors and treated with SR-4835 (0.5 µM) for 2 h and washed. 48 h later, cells were harvested, and total RNA was isolated. The expression levels of HIV Gag (unspliced), Tat (multiply spliced), and Env transcripts (single spliced) were analyzed by RT qPCR using specific primers. Data are presented as fold of change of mRNA normalized to DMSO and relative to GAPDH and TAR, indicating changes in transcript splicing balance upon SR-4835 treatment. Data are shown as fold change from independent donors. The dashed line represents cells treated with DMSO as a control.
